# *C*5-alkyl and *C*5-aryl Substituted 5-Deazaflavin as Sensitizers for Photodehalogenation of Aryl Halides

**DOI:** 10.3390/molecules31091400

**Published:** 2026-04-23

**Authors:** Huimin Guo, Xing Guan, Heping Li, Weihua Guo

**Affiliations:** School of Chemistry, State Key Laboratory of Fine Chemicals, Dalian University of Technology, Dalian 116024, China

**Keywords:** aryl halides, deazaflavin, flavin, photodehalogenation, photosensitizer

## Abstract

Aryl halides are important intermediates for chemical synthesis. However, the negative reduction potential up to −2.7 V (vs. SCE) makes photoredox conversion of aryl halides by reductive dehalogenation to aryl radicals for chemical transformations difficult. Inspired by the outstanding photophysical properties of deazaflavin and triphenylamine, as well as results of theoretical calculations, we attached the diphenylamino group to C8 of deazaflavin, and the resulting compounds look fabricated by “fusing” deazaflavin and triphenylamine (**TPA**) together by sharing the benzene ring. We also introduced alkyl and aryl moieties to *C*5 and afforded a series of deazaflavin derivatives (dFLs), namely 10-butyl-8-(diphenylamino)-3,5-dimethylpyrimido[4,5b]quinoline-2,4(3*H*,10*H*)-dione (**TPAdFlMe**), 10-butyl-8-(diphenylamino)-3-methyl-5-(trifluoromethyl)pyrimido[4,5-*b*]quinoline-2,4(3*H*,10*H*)-dione(**TPAdFlTF**) and 10-butyl-8-(diphenylamino)-3-methyl-5-phenylpyrimido[4,5-*b*]quinoline-2,4(3*H*,10*H*)-dione (**TPAdFlPh**), and investigated their photophysical properties and performance as sensitizers in the photodehalogenation of aryl halides. We showed that the photophysical properties are significantly improved in these dFLs. The absorption bands of dFLs are redshifted and the absorbance is more than double that of riboflavin tetraacetate (**RFTA**). The singlet oxygen quantum yields of **TPAdFlMe**, **TPAdFlTF** and **TPAdFlPh** are 0.42, 0.25 and 0.39, respectively, and the corresponding redox potentials are −1.75, −0.75 and −1.71 V vs. Ag/Ag^+^, respectively, comparable to known deazaflavin-based sensitizers. Originating from these properties, **TPAdFlMe** and **TPAdFlPh** are capable of sensitizing the full photodehalogenation of 0.038 mmol p-iodoanisole, and the yields of the photodehalogenation of 0.038 mmol p-bromoanisole are 67 and 69%, respectively. They also demonstrate exceptional performance in the photodehalogenation of halides of polycyclic aromatics with yields in the range of 73% for 1-benzhydryl-3-bromobenzene to 100% for 1-bromonapthalene in 18 h runs. The performance of **TPAdFlMe** and **TPAdFlPh** in photodehalogenation are already comparable to recently reported deazaflavin-based sensitizers, and we propose the transformation would proceed though the consecutive photo-induced electron transfer (*con*PET) mechanism with consecutive excitation of charged deazaflavin-based radicals under light irradiation as the key step to generating the aryl radicals, and the vital role of sensitizer-based radicals is further confirmed by mechanistic investigations. We expect the findings will help to design novel flavin-based triplet sensitizers for photoredox catalytic organic transformations.

## 1. Introduction

**Scheme 1:**
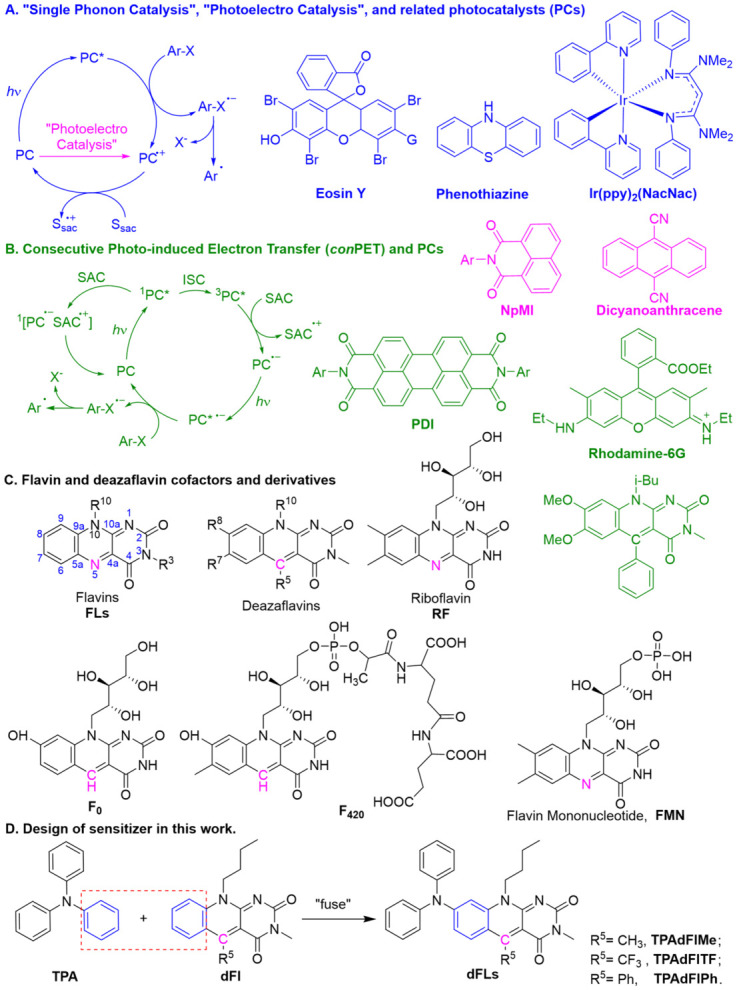
(**A**) Dehalogenation of aryl halides through “single-photon catalysis” (blue) and “photoelectro catalysis” (pink) pathways and typical PCs; (**B**) dehalogenation of aryl halides through consecutive photo-induced electron transfer and the reported PCs (green); (**C**) structures of the backbone of flavin and deazaflavin, and some naturally available flavin cofactors, namely **RF**, **F_0_**, **F_420_**, and **FMN**; (**D**) design of efficient deazaflavin-based sensitizers in this work. “*” stands for excited state, “·” stands for radical.

Synthetic chemists have long strived to develop chemical processes for the formation and scission of bonds, processes involving radical intermediates that are highly desired. Designed radical intermediates are highly active species with unpaired electrons, capable of reacting with a broad range of chemicals efficiently and selectively, enabling lots of chemical transformations that are difficult to achieve with conventional species and processes where electrons are paired, in an expedient fashion [1,2,3,4,5,6]. Owning to these advantages, the metal-free generation and utilization of radical intermediates at moderate conditions for chemical transformation is drawing considerable attention and becoming a new frontier in synthetic chemistry. Radical arylation is a typical reaction of this kind, involving the generation of aryl radicals and their further evolution by reactions with various substrates. Compared to conventional protocols for the generation of aryl radicals—which suffer from the usage of hazardous stoichiometric reagents, costly and toxic transition metals, harsh operating conditions and the use of strong bases, etc. [7]—the recently discovered photocatalytic protocols are more eco-benign, energy efficient and easy to realize, especially those utilizing visible light, and are of fundamental importance with significant practical value [5,6,7,8,9,10,11,12]. As not all precursors of aryl radicals or substrates can absorb the energy of light to generate radicals, photosensitizers (PSs) are commonly used in the photocatalytic protocols to harvest the energy of light radiation, and to transfer it into chemical energy through electron (ET) and/or energy transfer (EnT) with substrates, forming reactive intermediates (RIs), etc., to keep the chemical transformations efficient. As the decay of PS from triplet excited states (T*_n_*) to the ground state (S_0_) is spin-forbidden, triplet PSs are more efficient for EnT/ET with substrates, triggering cleavage of chemical bonds for the generation of desired radicals. Tremendous efforts are devoted to developing novel PSs for efficient chemical transformations (Scheme 1A,B). Though the redox potential of aryl halides can be up to −3 V vs. SCE (saturated calomel electrode), their direct photodehalogenation has been reported to proceed through *con*PET use, and aryl radicals were proposed as RIs (Scheme 1B) [13,14,15,16,17,18]. The semiquinone of dazaflavin as the radical species are formed by ET from the diisopropyl (ethyl) amine (DIPEA) donor to the PS at T_1_ [2,19,20,21,22,23,24,25]. The generated aryl radicals may be trapped chemically for further chemical transformations. Currently, the PSs available for the photodehalogenation of aryl halides via consecutive photo-induced electron transfer (*con*PET) are limited (Scheme 1A,B) [13,14,15,23,26,27,28,29]. Hence, photocatalysts for mild and efficient aryl radical generation from the wide range of aryl halides are of particular interest.

Flavin and its derivatives (FLs) are a family of compounds with a backbone of isoalloxazine that naturally exist as blue light receptors and cofactors in enzymes [30,31,32,33] and are the active sites of many biochemical processes in biological systems (Scheme 1C) [30,34,35,36]. Many FLs are efficient in sensitizing the oxidation of hydroxyl, amine, alkyl, alkoxy, ester, sulfide, hydrazine, phosphine moieties, etc., in organic compounds [37,38,39,40]. These transformations were proposed to initiate with the formation of RIs sensitized by FL-based PSs through ET, EnT or both [41,42,43]. The substitution of *N*5 of isoalloxazine with a C atom forms 5-deazaflavin that also acts as a coenzyme in organisms (Scheme 1C) [44,45]. Similar to FLs, deazaflavin also absorbs strongly in the visible light range, and fluoresces strongly at ~470 nm [15,46]. Deazaflavin derivatives are also eligible as photocatalysts, efficient in sensitizing the formation of ROSs [47,48,49] and cycloaddition of olefins [38,50,51], etc. The partially reduced deazaflavins are highly reactive [44,52,53,54], but are stable and function mainly as two-electron carriers when fully reduced [44,54,55,56,57,58], and can be synthesized or generated in situ [58,59,60]. The phenyl at *C*5 was found to be beneficial for lowering the redox potential of deazaflavin to ~−2.0 eV, making 5-phenyldeazaflavin superior reductants in photosynthesis [13,14,15,29]. Excited partially reduced deazaflavin derivatives are also capable of donating electrons for reductive the photodehalogenation of aryl halides via *con*PET (Scheme 1B) [13,14,15,29].

In principal, the efficiency of PS-sensitized transformations was determined by many factors, such as the light-harvesting ability, triplet quantum yields, stability of related intermediates, lifetime and the redox potential of the excited PSs, etc. [61,62,63]. Many attempts have been made to tailor the molecular structure of FLs and deazaflavin derivatives for application as sensitizers in photocatalysis [37,38,39]. Using [Ru(bpy)_3_]^2+^ as heavy atom-containing moieties attached to FL, we enhanced the ISC rates of FL and observed the phosphorescence emission from FL moieties [64,65]. We utilized the heavy atom effect of Br to boost the ISC from S_1_ in 7,8-dibromoflavin, more than doubling ^1^O_2_ quantum yield (Φ_Δ_) and achieving 2–5 times performance enhancement in the photooxidation of sulfides with respect to FL [66]. This PS also outperformed FL, 9-bromoflavin and 10-phenylflavin in sensitizing the photooxidative coupling of benzylic amines [67]. Amidation and alkylation at *N*5 and *N*10 were also found to be efficient at improving the performance of PSs with an alloxazine backbone [68,69]. We “fused” FL with naphthalimide, affording us NI-FL, with double the UV-Vis absorption and an elongated T_1_ lifetime that is capable of sensitizing the photooxidation of sulfide by ET and is 1–5 times more efficient than FL [70]. We recently attached -COOCH_3_ to C7, C8 and both FLs and found the resulting FL derivatives are efficient at sensitizing the oxidative coupling of various benzylic amines to imines [71]. These findings, and the reported photocatalytic organic transformations using FLs and deazaflavin derivatives, suggest the feasibility of improving the efficiency of PSs by tailoring the molecular structure rationally.

As inspired by the previous attempts to improve the efficiency of FL-based PSs by tailoring the molecular structure rationally, and the recent findings on deazaflavin derivative-sensitized the photodehalogenation of aryl halides as well, we designed several deazaflavin-based sensitizers (dFLs, Scheme 1D). Previously, 5-aryl and 7,8-methoxy substitution on benzene ring were proposed to have a positive effect on the photocatalytic activity/reduction potential of deazaflavins [13,14,15,16,17,18]. We attached diphenylamino to C8, so that the resulting deazaflavin derivatives look fabricated by “fusing” triphenylamine (TPA) that is commonly used as a donor in photovoltaic applications and strongly absorbs deazaflavin by sharing the benzene ring. We expect such a design would benefit the absorption of the resulting dFLs in the visible light range and their performance as reductants when converted to excited semiquinone radicals. We also introduced alkyl and aryl moieties to *C*5. These led to a series of dFLs afforded in this work, namely **TPAdFlMe**, **TPAdFlTF** and **TPAdFlPh** (Scheme 1D). We expected these would impact the reduction potential, photophysical properties and performance in photodehalogenation of aryl halides of these dFLs. Their photophysical properties and performance as sensitizers in the photodehalogenation of various aryl halides of these dFLs were investigated, combining experimental and theoretical efforts. We expect the findings will benefit the development of PSs for future photosynthesis.

## 2. Results and Discussions

**Scheme 2:**
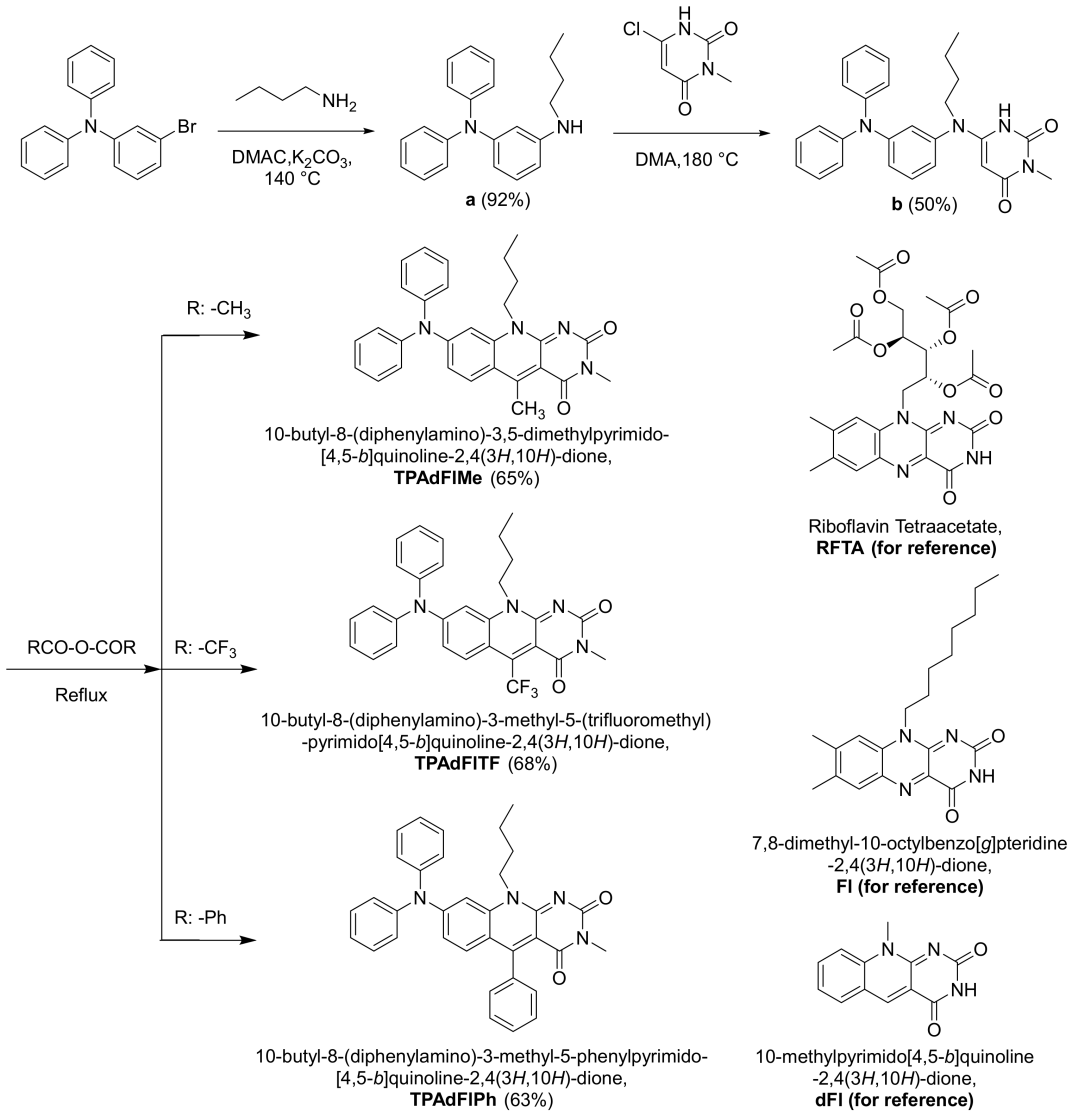
The structures and synthesis of **TPAdFLMe**, **TPAdFlTF**, and **TPAdFlPh**, and the structures of reference compounds for comparison, namely **RFTA**, **Fl** and **dFl**.

**Figure 1:**
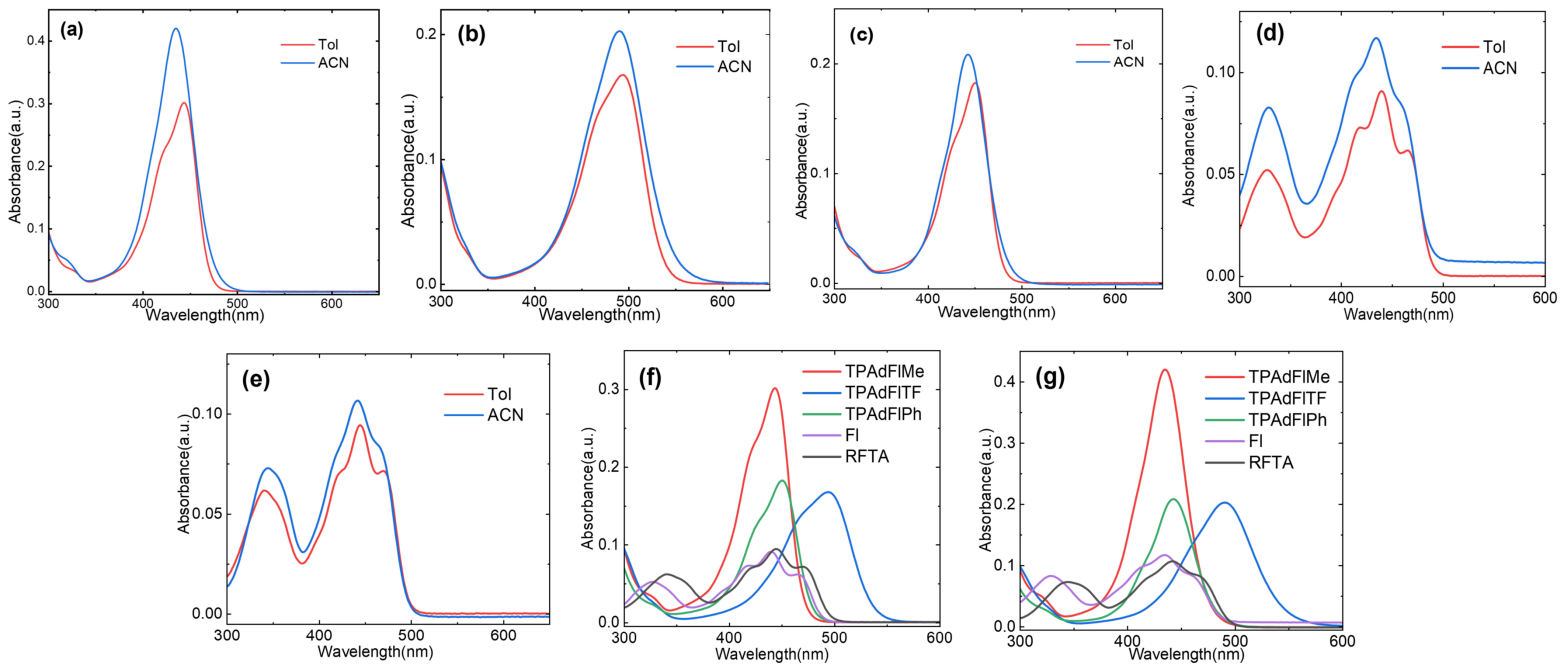
The experimental absorption spectra of **TPAdFlMe** (**a**), **TPAdFlTF** (**b**), **TPAdFlPh** (**c**), **Fl** (**d**) and **RFTA** (**e**) in Tol (red) and ACN (blue), and comparison of the experimental absorption spectra of all dFLs in Tol (**f**) and in ACN (**g**). (1 × 10^−5^ M, 25 °C).

The designed dFLs, namely **TPAdFlMe**, **TPAdFlTF** and **TPAdFlPh**, were afforded (Scheme 2 and Figures S3-1–S3-13 and S4). The absorption properties of these dFLs were investigated in toluene (Tol) and acetonitrile (ACN) first (Figure 1 and Table 1). There is only one absorption band in the range of 300 nm on the absorption spectra of dFLs (Figure 1a–c), while there are two absorption bands on the spectra of **Fl** and **RFTA** (Figure 1d,e). The absorption of these dFLs are stronger in ACN than in Tol. Furthermore, the absorption of **TPAdFlMe** is the strongest among these dFLs independent of the solvent used. The measured first absorption maxima (λ_abs_) of **TPAdFlMe**, **TPAdFlTF**, **TPAdFlPh**, **Fl** and **RFTA** in Tol are at 444, 490, 450, 439 and 447 nm, respectively, and are blue-shifted to 435, 490, 443, 434 and 442 nm, respectively. In this sense, the absorption properties of these dFLs are weakly solvent-dependent.

**Figure 2:**
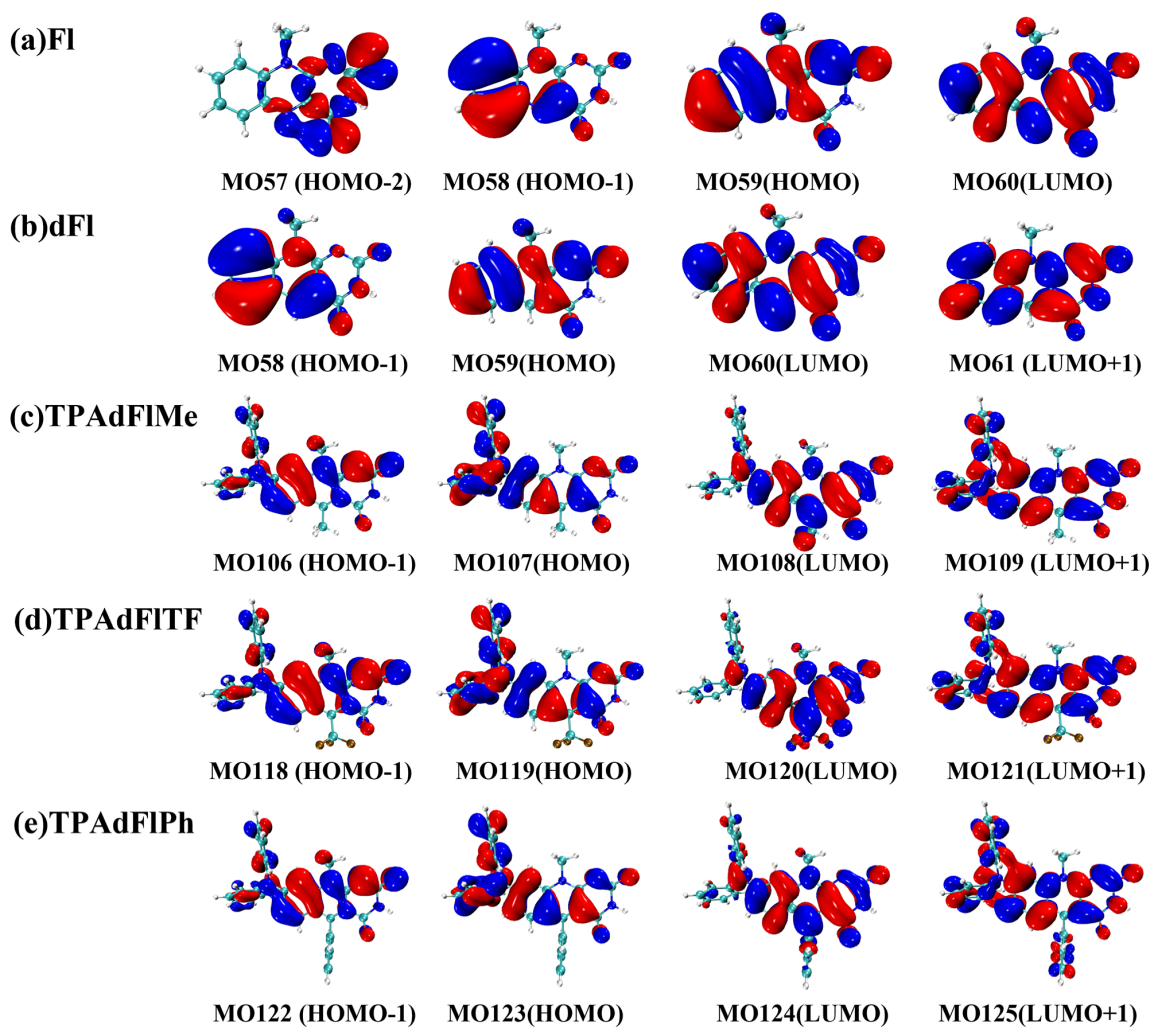
Isosurface plot of molecular orbitals contributing to low-energy electronic transitions corresponding to **Fl** (**a**), **dFL** (**b**), **TPAdFlMe** (**c**), **TPAdFlTF** (**d**) and **TPAdFlPh** (**e**). (C, O, N, and H atoms are in cyan, red, blue, and white, respectively. Isovalue: ±0.02 au). Isosurface plots of other frontier MOs can be found in Figures S6-1–S6-5.

According to results of TD-DFT-based calculations, λ_abs_ can be assigned to the S_0_→S_1_ transition of corresponding dFLs. For **Fl** in Tol, one absorption band with shoulders appears at 439 nm and another one is at 328 nm. These would shift to 435 and 329 nm, respectively, in ACN. The first two TD-DFT-calculated absorption maxima of **Fl** are at 410 and 331 nm, respectively. These two bands can be assigned to S_0_→S_1_ and S_0_→S_4_ transitions of π→π* and n→π* character, respectively. The S_0_→S_1_ transition is contributed by MO59 and MO60, which are the highest occupied molecular orbital (HOMO) and lowest unoccupied orbital (LUMO), respectively, while the S_0_→S_4_ transition is contributed by MO58 and MO60 (Table 1 and Table S6-1 and Figure 1, Figure 2 and Figure S6-1) [64,66,70]. The current results are consistent with those reported previously, suggesting the current theoretical approaches proposed are adequate to investigate the photophysics of dFLs (Figure 1) [72,73,74,75,76,77,78,79]. Benchmark calculations on vertical excitation energies of FL were also performed with range-separated CAM-B3LYP and ωB97XD, as well as the PBE hybrid (PBE0) functional, yielding essentially the same picture (Table S6-1 and Figure S6-1). The results of PBE0 functional are nearly the same with those of B3LYP functional. However, the vertical excitation energy obtained with CAM-B3LYP and ωB97XD functional are significantly different for predictive calculations [75,80,81,82]. With CAM-B3LYP functional, the S_0_→S_1_ and S_0_→S_2_ vertical excitation energies calculated are 3.43 (362 nm) and 4.29 eV (288 nm), respectively, and these are consistent with those from ωB97XD functional. As these are significantly different from theoretical and experimental results reported previously, CAM-B3LYP and ωB97XD functional are less eligible for predictive investigations [70,72,73,74,75,76,77,78,79]. This also agrees with the theoretical benchmark investigation [83]. Therefore, further calculations were performed with B3LYP functional.

The first two absorption bands of **dFL** are blue-shifted with respect to **Fl**, and can be assigned to S_0_→S_1_ and S_0_→S_3_ transitions of π→π* and n→π* character, involving MO58, MO59 and MO60 (Table 1 and Table S5-2, and Figure 2b and Figure S5-2) [84,85]. The consistency of the current results on **Fl** and **dFL** with those reported suggest the approaches used are adequate to investigate the photophysical properties of designed dFLs. The character of the HOMO and LUMO of Fl are well inherited by those of dFLs (Figure 2a,b). The attachment of the biphenylamino group to *C*8 and functional groups at *C*5 not only alter the energy levels and the spatial distribution of the MOs of dFLs, but also introduce new MOs and contribute to the electronic transitions. The absorption bands of **TPAdFLMe**, **TPAdFLTF**, and **TPAdFlPh** (Figure 1a–c) can all be assigned to the S_0_→S_1_ transition corresponding to transitions from the HOMO delocalized on TPA and the deazaflavin backbone, and the LUMO mainly contributed by the deazaflavin backbone (Figure 2c–e), so the absorption bands are redshifted with respect to **dFl**. In principle, the absorption intensity in terms of the molecular absorption coefficient (ε) is determined by the square of the dipole moment of the transition and the adiabatic excitation energy, while the oscillator strength, as the probability of the electronic transition taking place, is also directly proportional to both the square of the transition dipole moment and the adiabatic excitation energy. Therefore, the strong absorption **TPAdFlMe**, **TPAdFlTF**, and **TPAdFlPh** with respect to **Fl** and **dFl** can be explained with the more-than-tripled oscillator strength of the S_0_→S_1_ electronic transitions contributed by the TPA moiety (Table 1 and Figure 1).

**Figure 3:**
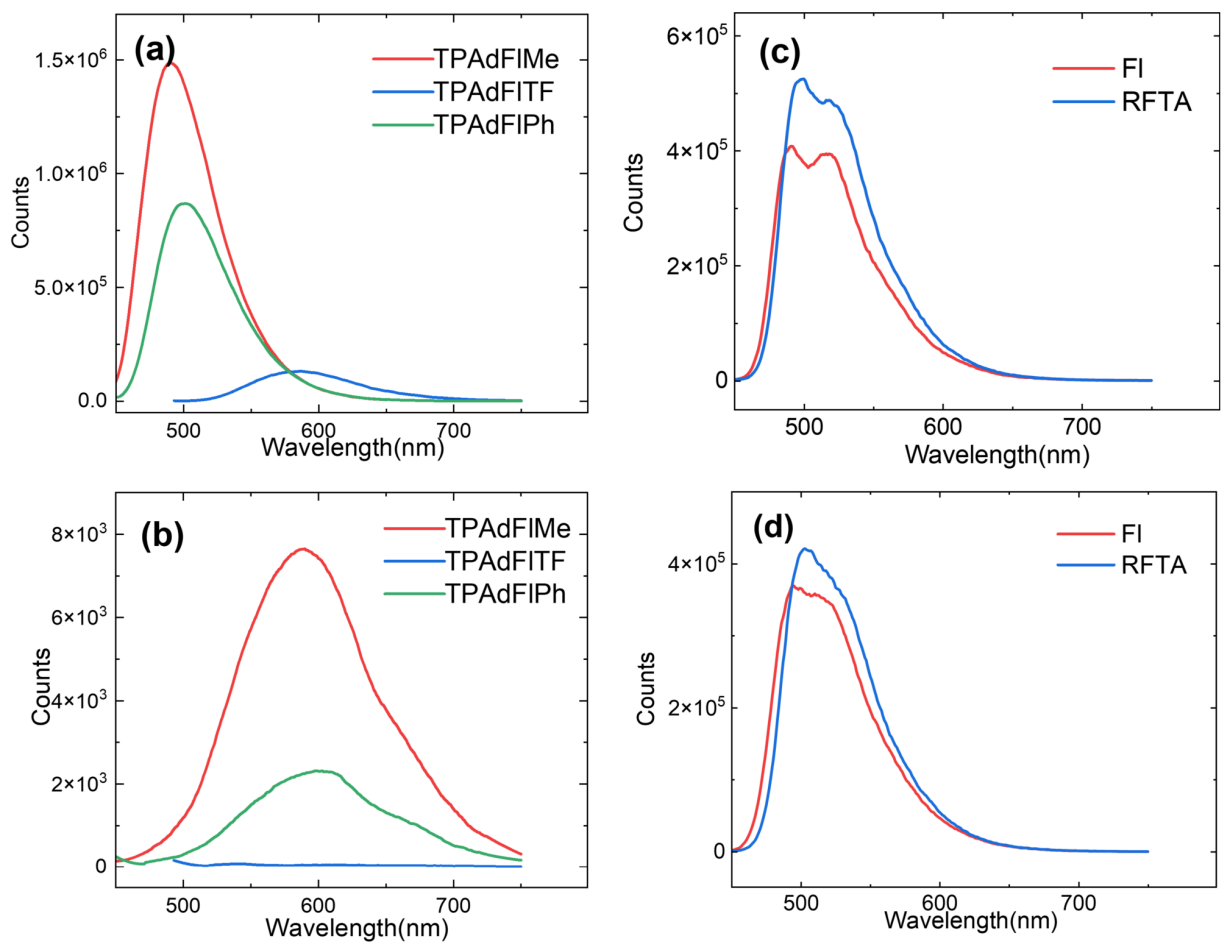
Emission spectra of **TPAdFlMe**, **TPAdFlTF** and **TPAdFlPh** in Tol (**a**) and ACN (**b**), and emission spectra of Fl and RFTA in Tol (**c**) and ACN (**d**). λ_ex_ used for **TPAdFlMe**, **TPAdFlTF**, **TPAdFlPh**, **Fl** and **RFTA** are 443, 492, 450, 438 and 447nm, respectively. c = 10^−5^ mol/L, 25 °C.

The fluorescence emission bands of deazaflavin derivatives exhibit a hypsochromic shift compared to **Fl** and **RFTA** (Figure 3 and Table 2). The effect of substitution at *C*5 on the fluorescence maxima follows the trends as aforementioned for the absorption spectra. The CF_3_- moiety causes a significant bathochromic shift in the fluorescence maxima to the value of 585 cm^−1^. The same change in λ_abs_, λ_em_ and Φ_F_ was reported in *C*5-substituted deazaflavin derivatives with variation in the group on *C*5 from methyl to trifluoromethyl [15]. Among the dFLs investigated, a high fluorescence quantum yield (Φ_F_) of 82% was observed for **TPAdFlMe** and the lowest Φ_F_ of 17% was found for **TPAdFlTF**, considering that the Φ_F_ of **Fl** and **RFTA** are 42% and 30%, respectively. The singlet oxygen quantum yield (Φ_△_) of **RFTA** is 76% and is the highest among the compounds investigated, and the Φ_△_ of **TPAdFlMe**, **TPAdFlTF** and **TPAdFlPh** are 42%, 25% and 39%, respectively. In this sense, **TPAdFlMe** and **TPAdFlPh** are promising PSs in sensitizing the formation of ^1^O_2_, already superior to the recently reported deazaalloxazines and deazaflavin derivatives, and would exhibit performance like those of **Fl** in photooxidation [13,14,15,29]. The measured redox potential (E_re_) of dFLs falls in the range of −0.71 V for **RFTA** to −1.75 V for **TPAdFlMe** (Figure S4). In this sense, the E_re_ of **TPAdFlMe** and **TPAdFlPh** in ACN vs. SCE would be −1.40 and −1.36 V, respectively, and is comparable with those of the reported 5-aryl deazaflavins (Table 2). Therefore, we expect **TPAdFlMe** and **TPAdFlPh** would exhibit performance comparable or even superior to those deazaflavin derivatives reported before in the photodehalogenation of aryl halides.

Inspired by the photophysical and electrochemical properties of **TPAdFlMe**, **TPAdFlPh**, **Fl** and **RFTA**, their performance in sensitizing the photodehalogenation of aryl halides was evaluated. In these reactions, **Ce_2_CO_3_** and **DIPEA** were used as a halide trap and sacrificial electron donor, respectively [13,14,15,29]. We started with the optimization of various factors, such as PS, solvents, reaction time, etc., to improve the conversion of aryl halides (Table 3, Figures S5-1–S5-21). We adapted the reported amount of 8 mol% dFLs, which is enough to convert 0.038 mmol aryl halides in 24 h [13,14,15,29]. In parallel experiments using **TPAdFlMe**, **TPAdFlPh**, **Fl** and **RFTA** as the PSs, **TPAdFlPh** outperforms other PSs in the photodehalogenation of bromobenzene in 18 h and the yield is 82%. The yield with **TPAdFlMe** is slightly lower (80%). The yield is 0 when Fl and RFTA are used (Table 3, Entries 1–4) [13,14,15,29]. The photodehalogenation of bromobenzene using **TPAdFlMe** as the PS was then performed in different solvents, including toluene, dichloromethane, ACN, methanol and a mixture of ACN and methanol. The highest yield of 85% was found in ACN. Considering the similar performance of **TPAdFlMe** and **TPAdFlPh** in 18 h photodehalogenation runs, ACN was selected as the solvent for further investigation (Table 3, entries 5–9). The impact of the reaction duration was also investigated (Table 3, entries 1–2, 7, 10–18). When **TPAdFlMe** is used, the benzene yield is 79% in 15 h, and this further increases to 80% and 85% after 18 and 24 h, respectively. When **TPAdFlPh** is used, the benzene yield is 83% at 15 h, and this further changes to 82% and 88%, respectively, in 18 and 24 h runs. These suggest the reaction duration should be at least 18 h to get a significant yield, and further elongation would also be beneficial. Parallel experiments were also performed without PSs or light irradiation using bromobenzene, chlorobenzene and iodobenzene as the substrate in 24 h batch runs (Table 3, entries 19–22). However, no reaction takes place in these cases, suggesting the vital role of light irradiation and dFLs as PSs for the photodehalogenation of aryl halides.

Considering the outstanding performance of **TPAdFlMe** and **TPAdFlPh** in the photodehalogenation of bromobenzene, they were also used to sensitize the dehalogenation of various aryl halides (Table 4 and Figures S5-22–S5-57). For most of the substrates considered, **TPAdFlMe** and **TPAdFlPh** are capable of converting a considerable amount of the aryl halide substrates, with yields larger than 60% in most cases. It is interesting to note that the yield when **TPAdFlPh** was used is always slightly better than that of **TPAdFlMe** in the conversion of most of the aryl halides, except for chlorobenzene and 1-benzhydryl-3-bromobenzene. This may be attributed to their slight difference in E_re_. Previously, C-5 phenyl derivatives of deazaflavin were reported to exhibit better performance in the photodehalogenation of aryl halides than other deazaflavin derivatives [13,14,15,29]. The performance data of **TPAdFlMe** and **TPAdFlPh** are already superior to those recently reported for 10-unsubstituted 3,7,8-trimethylisoalloxazine anion [29], and are comparable to those reported for 10-butyl-7,8-dimethoxy-3-methyl-5-phenyl-deazaflavin that is capable of converting p-chloroanisole and p-bromoanisole with yields for both of 80% in 18 h runs, and 82% and 98% in 24 h runs [13,15]. The difference may be attributed to the different contribution of TPA and methoxy to the photophysical properties of the deazaflavin derivatives and the slight difference in the reaction setup. Furthermore, **TPAdFlMe** and **TPAdFlPh** demonstrate exceptional performance in the photodehalogenation of halides of polycyclic aromatics, such as 3-bromopyridine (entries 15–16), 1-bromonapthalene (entries 17–18), 4-bromopyrene (entries 19–20), 1-benzhydryl-3-bromobenzene (entries 21–22), etc., with yields in the range of 73% for 1-benzhydryl-3-bromobenzene to 100% for 1-bromonapthalene in 18 h runs.

The performance of **TPAdFlMe** and **TPAdFlPh** in the dehalogenation of aryl polyhalides was also investigated (Table 5, Table 6 and Table 7 and Figures S5-58–S5-105). For the polyhalides investigated, it is rather general that monohalide intermediates are formed as the major product or the only product when the concentration of PS, **DIPEA** and **Cs_2_CO_3_** are low in 18 h runs, and the yield of fully reduced aryl compounds increases with the concentration of PS, **DIPEA** and **Cs_2_CO_3_** and the elongation of the reaction duration. The impact of the concentration of PS, **DIPEA and Cs_2_CO_3_** and the reaction duration can be found for the photodehalogenation of 1,2-dichlorobenzene (Table 5, Entries 1–4), 1,3-dichlorobenzene (Table 5, Entries 5–10), 1,3-dibromobenzene (Table 5, Entries 11–14) and 4,4′-Diiodobiphenyl (Table 7, Entries 1–4), while 1,3-diiodobenzene and 3,3′-Dibromobiphenyl (Table 6) were completely converted to corresponding aryl compounds at the conditions investigated. This suggests that the photodehalogenation of aryl polyhalides depends strongly on the reaction duration, and the concentration of PS, **DIPEA** and **Cs_2_CO_3_**, and that it is possible to control the product selectivity by careful optimization of the operating conditions. Further to these, the product yields when **TPAdFlPh** was used are not always higher than those with **TPAdFlMe**, even when the reactions were performed in parallel with the same operating conditions, suggesting the measured performance of PSs is the overall effect of the interplay of many controlling factors, owing to the complicated *con*PET mechanism of deazaflavin-sensitized photodehalogenation that was proposed to solve the concern of the mismatch of the E_re_ of PS and the substrate [15,19].

**Scheme 3:**
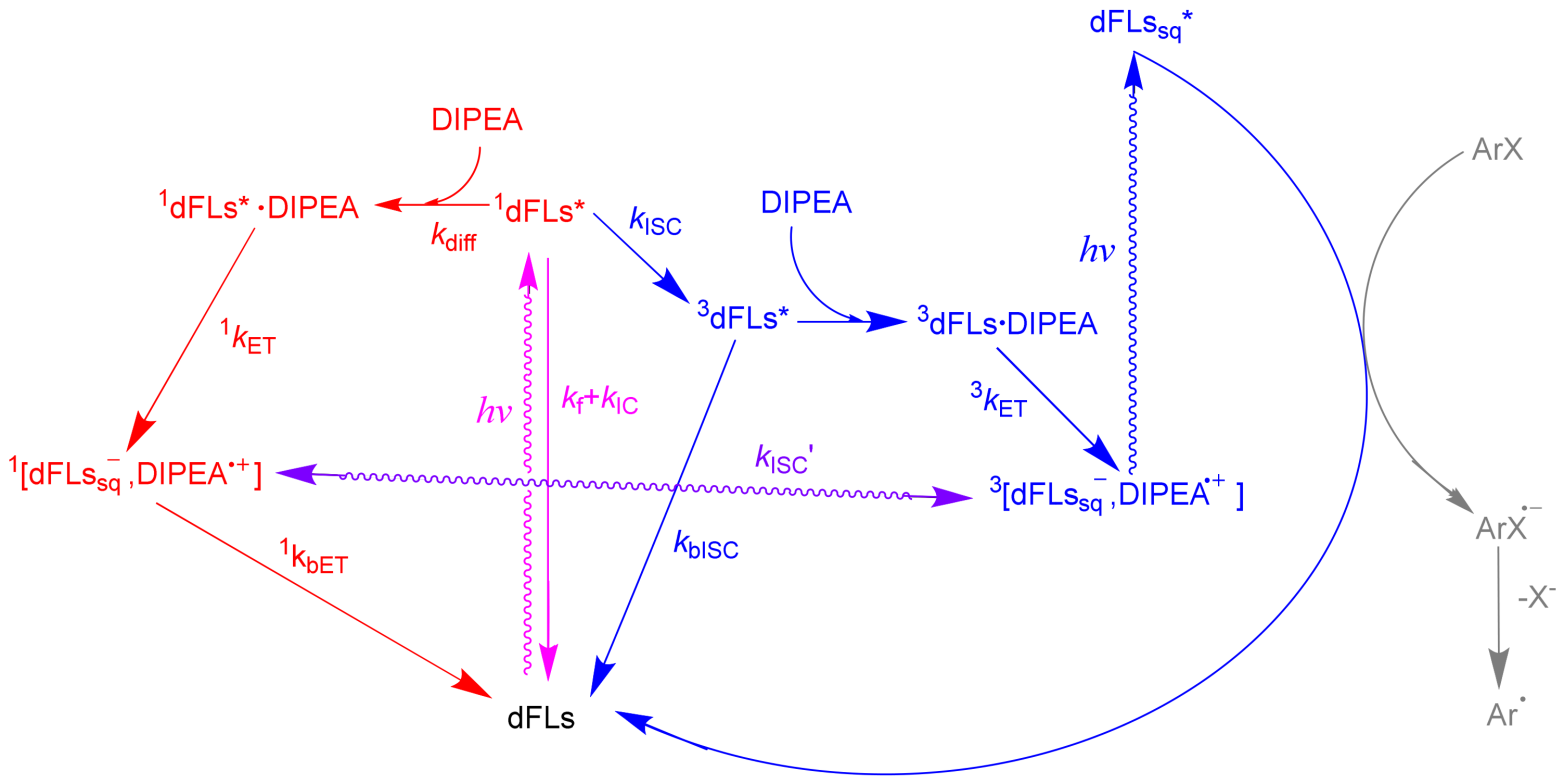
Potential pathways for the photodehalogenation of aryl halides with dFLs as sensitizers. dFLs would absorb energy of photon and get populated to its singlet excited state (^1^dFLs*) and the excited dFLs also decay radiatively or by internal conversion to its ground state (pink). DIPEA, as a sacrificial electron donor, reacts either with the excited singlet (^1^dFLs*) or triplet (^3^dFLs*) state of the dFLs to initiate the pathways. On the less productive ^1^dFLs* pathway (red), a radical pair, ^1^[dFLs_sq_^−^, DIPEA^•+^], is formed after ET and would recombine faster than its formation. On the ^3^dFLs* pathway (blue), ET forms ^3^[dFLs_sq_^−^, DIPEA^•+^] would accumulate, considering the stability of dFLs_sq_. This would enable the adsorption of a second photon by dFLs_sq_ and a consecutive photo-induced electron transfer from dFLs_sq_* to the aryl halide substrate, restoring dFLs to the ground state and closing the photocatalytic cycle, and initiating dehalogenation for formation of aryl radicals for further reactions (gray).

In the proposed *con*PET mechanism (Scheme 3) in the photodehalogenation of aryl halides sensitized by deazaflavin derivatives, DIPEA is the sacrificial electron donor and would initiate the catalytic cycle by ET to the PS at excited states. The ET with PS at singlet excited states is not productive due to the fast backward ET to DIPEA. However, the ET with PS at triplet states would form a radical pair, where the negatively charged PS radical interacts with the positively charged DIPEA radical. As the decay of the triplet radical pair to the ground state is spin-forbidden, it would be accumulated in the reaction system [84,86]. Further light irradiation would pop this PS-related species to the spin-allowed excited states, enabling a consecutive photo-induced electron transfer to the aryl halide substrate. The photocatalytic cycle is closed by the regeneration of PS to its ground state. The further evolution of the charged aryl halide leads to dehalogenation by the formation of an aryl radical and a halide ion. In parallel mechanism investigations, 2,2,6,6-tetramethylpiperidinyl-1-oxy (TEMPO) which is a radical scavenger was added to the reaction system to inhibit the photodehalogenation, considering the vital role of the negatively charged PS radical in *con*PET (Table 8). The gradual decrease in yield with the amount of TEMPO used confirms the vital role of the PS-related radical in photodehalogenation through the *con*PET mechanism [67,70,71]. Further mechanism investigations using time-resolved spectra and theoretical calculations are being performed to provide additional evidence to support the *con*PET mechanism and to guide the design of PSs for the efficient photodehalogenation of aryl halides. The results will be published when available.

The dehalogenation of ArX is initiated by the formation of ArX^−•^ as RIs by ET from dFLs_sq_* to ArX substrate. The energy level of dFLs_sq_* must match that of ArX to facilitate efficient ET (redox potential), and this is vital for dehalogenation. Considering this, the performance data indicates the ArX converted with the lowest redox potential is p-chloroanisole (E_re_ = –2.88 eV); this may set the lower limit for ArX to be converted with designed dFLs. Furthermore, higher concentrations of dFLs_sq_* would benefit the efficiency of ET. A high concentration of Cs_2_CO_3_ as the halide trap would promote the formation of Ar^•^. The lifetime of dFLs_sq_ should be long enough to get accumulated and populated to dFLs_sq_* by a second phonon. dFLs_sq_ is formed by ET from DIPEA. Apart from the energy level requirement for the ET (redox potential), high triplet quantum yield and slow radiative and non-radiative of ^3^dFLs may promote the efficiency of dFLs_sq_ formation. Slow fluorescence emission and internal conversion, and fast intersystem crossing to energy allowed ^3^dFLs and slow phosphorescent emission and non-radiative decay of ^3^dFLs may help to increase the triplet quantum yield of dFLs and benefit the subsequent ET for dFLs_sq_ formation. The strong adsorption of dFLs in the visible light range and redshifted adsorption may make the conversion utilizing sunlight possible. For the dFLs designed in this work, the non-zero conversion of *p*-chloroanisole confirms their effectiveness for the photodehalogenation of ArX. Higher triplet quantum yields and reasonable fluorescence quantum yields of **TPAdFlMe** and **TPAdFlPh** with respect to reported PSs are found, and are vital in dFLs_sq_ formation. These factors may act together with other factors, such as operating conditions, etc., in determining the performance of the dFLs in the photodehalogenation of ArX.

## 3. Materials and Methods

### 3.1. Materials and Synthesis

The designed dFLs (Scheme 2) were afforded and characterized (Figures S3-1–S3-13 and S4).

### 3.2. Theoretical Methods

Density functional theory (DFT) and time-dependent DFT (TD-DFT)-based calculations were performed to investigate the absorption properties of dFLs (Scheme 1). We used methyl to simplify the alkyl moieties. We fully relaxed the dFLs at B3LYP/6-311G(d) level of theory to get their ground state (S_0_) structures [87,88,89,90]. In the calculations, we used Polarizable Continuum Model (PCM) to treat the acetonitrile (CH_3_CN) solvent [91]. The absorption properties of dFLs were investigated with TD-DFT-based calculations at the same level of theory. Frequency calculations were performed for all reported structures to insure the nature of minima on the potential energy surface (PES). All the DFT and TD-DFT-based calculations were performed with Gaussian 16 [92].

## 4. Conclusions

Stimulated by the significant role of aryl radicals in organic synthesis, and the recent pioneer works on the photodehalogenation of aryl halides, we designed and afforded **TPAdFlMe**, **TPAdFlTF** and **TPAdFlPh**, and investigated their photophysical properties and performance as sensitizers in the photodehalogenation of aryl halides. The attachment of diphenylamino to *C*8 does enhance the absorption of these dFLs in the visible range, and benefits the intersystem crossing to triplet states. The absorption bands of these dFLs are redshifted and the absorbance is more than doubled compared with that of **RFTA**. The redox potentials of **TPAdFlMe**, **TPAdFlTF** and **TPAdFlPh** are more negative than **RFTA**. The performances of **TPAdFlMe** and **TPAdFlPh** are already comparable to recently reported deazaflavin-based sensitizers in the photodehalogenation of aryl halides. We propose that dFL-sensitized photodehalogenation would proceed though the *con*PET mechanism, with consecutive excitation of charged PS-based radicals under light irradiation as the key step to generating aryl radicals. We expect the current findings will help to design novel flavin-based triplet sensitizers for photoredox catalytic organic transformations. Further mechanistic investigations combining time-resolved spectra and theoretical calculations are being performed in our lab to provide additional evidence to support the *con*PET mechanism and to guide the design of PSs for the efficient photodehalogenation of aryl halides. The results will be published when available. The performance of a triplet PS in photocatalysis depends on many factors, such as the long triplet lifetime, suitable redox potential, and reasonable stability and binding with substrate molecules to facilitate efficient ET and the future evolution of the molecular composites, etc. We will work continuously to generalize the strategies for tailoring chromophores for application as heavy atom-free triplet sensitizers in photocatalysis.

## Figures and Tables

**Table 1:** Electronic transitions involved in UV-Vis spectra of dFLs *^a^*.

	Energy	f *^b^*	Composition	Percentage *^c^*	Character
Fl:					
S_0_→S_1_	3.02 eV/410 nm	0.2013	59→60	96.54%	π→π*
S_0_→S_4_	3.75 eV/331 nm	0.1821	58→60	92.42%	n→π*
S_0_→T_1_	2.15 eV/576 nm	0.0000	59→60	96.79%	π→π*
S_0_→T_2_	2.73 eV/454 nm	0.0000	58→60	86.92%	n→π*
S_0_→T_3_	2.90 eV/428 nm	0.0000	56→60	69.71%	n→π*
			57→60	18.76%	n→π*
			52→60	8.25%	n→π*
dFl:					
S_0_→S_1_	3.28 eV/378 nm	0.2220	59→60	96.82%	π→π*
S_0_→S_3_	4.03 eV/307 nm	0.1507	58→60 59→61	89.60% 7.99%	π→π* π→π*
S_0_→T_1_	2.53 eV/490 nm	0.0000	59→60	94.62%	π→π*
S_0_→T_2_	2.92 eV/425 nm	0.0000	58→60	84.28%	π→π*
			59→61	4.92%	π→π*
TPAdFlMe:					
S_0_→S_1_	2.86 eV/433 nm	0.7205	107→108	98.30%	π→π*
S_0_→S_4_	3.93 eV/315 nm	0.1137	58→60	84.28%	π→π*
			59→61	4.92%	π→π*
S_0_→T_1_	2.25 eV/551 nm	0.0000	107→108	93.66%	π→π*, n→π*
S_0_→T_2_	2.84 eV/437 nm	0.0000	118→120	90.23%	π→π*, n→π*
			119→120	2.32%	π→π*, n→π*
TPAdFlTF:					
S_0_→S_1_	2.56 eV/485 nm	0.6648	119→120	98.80%	π→π*
S_0_→S_2_	3.06 eV/406 nm	0.0405	118→120	97.40%	π→π*, n→π*
S_0_→T_1_	1.85 eV/671 nm	0.0000	119→120	92.77%	π→π*, n→π*
S_0_→T_2_	2.51 eV/493 nm	0.0000	118→120	90.23%	π→π*, n→π*
			119→120	2.32%	π→π*, n→π*
TPAdFlPh:					
S_0_→S_1_	2.86 eV/434 nm	0.7562	123→124	98.24%	π→π*
S_0_→S_4_	3.91 eV/317 nm	0.0708	123→125	66.26%	π→π*, n→π*
			120→124	28.16%	π→π*, n→π*
S_0_→T_1_	2.21 eV/561 nm	0.0000	123→124	93.62%	π→π*, n→π*
S_0_→T_2_	2.82 eV/440 nm	0.0000	122→124	87.17%	π→π*, n→π*
			123→125	2.59%	π→π*, n→π*

*^a^* For clearance, only first 2 S_0_→S_n_ transitions with f > 0.05 and S_0_→T_n_ transitions with energy lower than that of S_0_→S_1_ transition were shown. Low-energy transitions of dFLs are summarized in Tables S6-1–S6-4. *^b^* Oscillator strength. *^c^* Contribution of the electronic transitions in percentage, calculated as ${CI}^{2}/0.5\times 100\%$, where CI is the calculated CI coefficient.

**Table 2:** The measured photophysical properties of dFLs.

	λ_abs_ /nm *^a^*	e /L·mol^−1^·cm^−1 *b*^	λ_em_ /nm *^c^*	Φ_F_ /% *^d^*	Φ_D_ /% *^e^*	τ_F_ /ns *^f^*	E_re_ /v *^g^*
**TPAdFlMe**	444	3.00 × 10^4^	491	82	42	3.97	−1.75(−1.40)
**TPAdFlTF**	490	1.68 × 10^4^	585	17	25	4.26	−0.75(−0.40)
**TPAdFlPh**	450	1.82 × 10^4^	500	63	39	4.20	−1.71(−1.36)
**Fl**	439	0.51 × 10^4^	524	42	38	6.13	−0.64 *^h^*
**RFTA**	447 *^h^*	0.67 × 10^4^ *^h^*	529 *^h^*	30 *^h^*	76 *^h^*	6.28 *^h^*	−0.79 *^h^*

*^a^* Wavelength at first absorption maxima in UV-Vis absorption spectra in toluene. *^b^* Molar absorption coefficient. *^c^* Wavelength at emission maxima in toluene. *^d^* Fluorescence quantum yield in toluene. *^e^* Singlet oxygen quantum yield in toluene. *^f^* Fluorescence lifetime in toluene. *^g^* Reduction potential, measured vs. Ag/AgCl (0.1 M KCl) electrode in CH_2_Cl_2_; the numbers in parentheses were converted to value in ACN with respect to SCE. *^h^* Literature value taken from Ref. [15], measured in ACN, with respect to SCE.

**Table 3:** Optimization of dFL-sensitized photodehalogenation of aryl monohalides *^a^*.

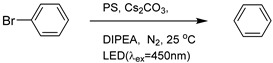					
Entry	PS	Solvent	Time (h)	Substrate	Yield (%) *^b^*
1	**TPAdFlMe**	ACN	18	Bromobenzene	80
2	**TPAdFlPh**	ACN	18	Bromobenzene	82
3	**Fl**	ACN	18	Bromobenzene	0
4	**RFTA**	ACN	18	Bromobenzene	0
5	**TPAdFlMe**	Tol	24	Bromobenzene	0
6	**TPAdFlMe**	DCM	24	Bromobenzene	0
7	**TPAdFlMe**	ACN	24	Bromobenzene	85
8	**TPAdFlMe**	ACN:MeOH = 9:1	24	Bromobenzene	49
9	**TPAdFlMe**	MeOH	24	Bromobenzene	0
10	**TPAdFlMe**	ACN	6	Bromobenzene	37
11	**TPAdFlMe**	ACN	9	Bromobenzene	47
12	**TPAdFlMe**	ACN	12	Bromobenzene	49
13	**TPAdFlMe**	ACN	15	Bromobenzene	79
14	**TPAdFlPh**	ACN	6	Bromobenzene	44
15	**TPAdFlPh**	ACN	9	Bromobenzene	47
16	**TPAdFlPh**	ACN	12	Bromobenzene	73
17	**TPAdFlPh**	ACN	15	Bromobenzene	83
18	**TPAdFlPh**	ACN	24	Bromobenzene	88
19	**None**	ACN	24	Bromobenzene	0
20 *^c^*	**TPAdFlPh**	ACN	24	Bromobenzene	0
21 *^c^*	**TPAdFlPh**	ACN	24	Chlorobenzene	0
22 *^c^*	**TPAdFlPh**	ACN	24	Iodobenzene	0

*^a^* Reaction conditions: substrates (0.038 mmol), PS (8 mol%), DIPEA (26 μL, 2.0 equiv.), Cs_2_CO_3_ (12 mg, 1.0 equiv.), λ_ex_ = 450 nm, 25 °C, N_2_ atmosphere. *^b^* Yields were determined via calibrated GC-analysis. *^c^* No light.

**Table 4:** dFL-sensitized dehalogenation of aryl halides *^a^*.

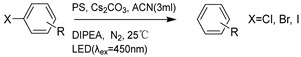				
Entry	PS	Time (h)	Substrate	Yield (%) *^b^*
1	**TPAdFlMe**	24	Chlorobenzene	80
2	**TPAdFlPh**	24	Chlorobenzene	60
3	**TPAdFlMe**	24	Bromobenzene	85
4	**TPAdFlPh**	24	Bromobenzene	88
5	**TPAdFlMe**	18	Iodobenzene	100
6	**TPAdFlPh**	18	Iodobenzene	100
7	**TPAdFlMe**	18	p-chloroanisole	49
8	**TPAdFlPh**	18	p-chloroanisole	62
9	**TPAdFlMe**	18	p-bromoanisole	67
10	**TPAdFlPh**	18	p-bromoanisole	69
11	**TPAdFlMe**	24	p-bromoanisole	71
12	**TPAdFlPh**	24	p-bromoanisole	87
13	**TPAdFlMe**	18	p-iodoanisole	100
14	**TPAdFlPh**	18	p-iodoanisole	100
15	**TPAdFlMe**	18	3-bromopyridine	92
16	**TPAdFlPh**	18	3-bromopyridine	97
17	**TPAdFlMe**	18	1-bromonapthalene	96
18	**TPAdFlPh**	18	1-bromonapthalene	100
19	**TPAdFlMe**	18	4-bromopyrene	96
20	**TPAdFlPh**	18	4-bromopyrene	97
21	**TPAdFlMe**	18	1-benzhydryl-3-bromobenzene	86
22	**TPAdFlPh**	18	1-benzhydryl-3-bromobenzene	73

*^a^* Reaction conditions: substrates (0.038 mmol), PS (8 mol%), DIPEA (26 μL, 2.0 equiv.), Cs_2_CO_3_ (12 mg, 1.0 equiv.), λ_ex_ = 450 nm, 25 °C, N_2_ atmosphere. *^b^* Yields were determined via calibrated GC-analysis.

**Table 5:** dFL-sensitized dehalogenation of aryl dihalides *^a^*.

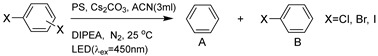								
Entry	PS	PS (mol%)	DIPEA (mmol)	Cs_2_CO_3_ (mmol)	Time (h)	Substrate	Yield A (%) *^b^*	Yield B (%) *^b^*
1	**TPAdFlMe**	16	0.152	0.076	18	1,2-dichlorobenzene	0	76
2	**TPAdFlPh**	16	0.152	0.076	18	1,2-dichlorobenzene	0	95
3	**TPAdFlMe**	16	0.152	0.038	24	1,2-dichlorobenzene	62	36
4	**TPAdFlPh**	16	0.152	0.038	24	1,2-dichlorobenzene	94	6
5	**TPAdFlMe**	8	0.076	0.038	18	1,3-dichlorobenzene	0	50
6	**TPAdFlPh**	8	0.076	0.038	18	1,3-dichlorobenzene	59	13
7	**TPAdFlMe**	8	0.076	0.038	24	1,3-dichlorobenzene	60	9
8	**TPAdFlPh**	8	0.076	0.038	24	1,3-dichlorobenzene	61	17
9	**TPAdFlMe**	16	0.152	0.038	24	1,3-dichlorobenzene	60	34
10	**TPAdFlPh**	16	0.152	0.038	24	1,3-dichlorobenzene	33	63
11	**TPAdFlMe**	8	0.076	0.038	18	1,3-dibromobenzene	0	96
12	**TPAdFlPh**	8	0.076	0.038	18	1,3-dibromobenzene	0	100
13	**TPAdFlMe**	16	0.152	0.038	24	1,3-dibromobenzene	53	47
14	**TPAdFlPh**	16	0.152	0.038	24	1,3-dibromobenzene	0	100
15	**TPAdFlMe**	8	0.076	0.038	18	1,3-diiodobenzene	94	0
16	**TPAdFlPh**	8	0.076	0.038	18	1,3-diiodobenzene	100	0

*^a^* Reaction conditions: substrates (0.038 mmol), λ_ex_ = 450 nm, 25 °C, N_2_ atmosphere. *^b^* Yields were determined via calibrated GC-analysis.

**Table 6:** dFL-sensitized dehalogenation of 3,3′-Dibromobiphenyl *^a^*.

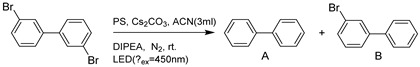							
Entry	PS	PS (mol%)	DIPEA (mmol)	Cs_2_CO_3_ (mmol)	Time (h)	Yield A (%) *^b^*	Yield B (%) *^b^*
1	**TPAdFlMe**	16	0.152	0.038	24	100	0
2	**TPAdFlPh**	16	0.152	0.038	24	96	4

*^a^* Reaction conditions: substrates (0.038 mmol), λ_ex_ = 450 nm, 25 °C, N_2_ atmosphere. *^b^* Yields were determined via calibrated GC-analysis.

**Table 7:** dFL-sensitized dehalogenation of 4,4′-Diiodobiphenyl *^a^*.

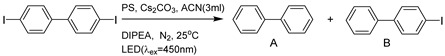							
Entry	PS	PS (mol%)	DIPEA (mmol)	Cs_2_CO_3_ (mmol)	Time (h)	Yield A (%) *^b^*	Yield B (%) *^b^*
1	**TPAdFlMe**	8	0.076	0.038	18	38	62
2	**TPAdFlPh**	8	0.076	0.038	18	28	72
3	**TPAdFlMe**	16	0.152	0.076	18	100	0
4	**TPAdFlPh**	16	0.152	0.076	18	100	0

*^a^* Reaction conditions: substrates (0.038 mmol), λ_ex_ = 450 nm, 25 °C, N_2_ atmosphere. *^b^* Yields were determined via calibrated GC-analysis.

**Table 8:** dFL-sensitized dehalogenation of bromobenzene *^a^*.

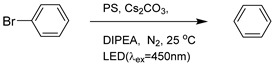				
Entry	PS	Time (h)	TEMPO (mol%)	Yield (%) *^b^*
1	**TPAdFlMe**	18	0	80
2	**TPAdFlMe**	18	8	82
3	**TPAdFlMe**	18	80	79
4	**TPAdFlMe**	18	160	62
5	**TPAdFlMe**	18	240	0
6	**TPAdFlPh**	18	0	82
7	**TPAdFlPh**	18	8	83
8	**TPAdFlPh**	18	80	74
9	**TPAdFlPh**	18	160	62
10	**TPAdFlPh**	18	240	18

*^a^* Reaction conditions: substrates (0.038 mmol), PS (8 mol%), DIPEA (26 μL, 2.0 equiv.), Cs_2_CO_3_ (12 mg, 1.0 equiv.), λ_ex_ = 450 nm, 25 °C, N_2_ atmosphere. *^b^* Yields were determined via calibrated GC-analysis (Figures S5-106–S5-117).

## Data Availability

The original contributions presented in this study are included in the article/Supplementary Materials. Further inquiries can be directed to the corresponding author(s).

## Supplementary Materials

The following supporting information can be downloaded at: https://www.mdpi.com/article/10.3390/molecules31091400/s1, 1. General Information; 2. Synthesis and Molecular Structure Characterization Data; 3. NMR and MS spectra; 4. Electrochemical measurements; 5. GC spectra; 6. DFT/TD-DFT Results; 7. References.
