# Supplementary material for: C5-alkyl and C5-aryl Substituted 5-Deazaflavin as Sensitizers for Photodehalogenation of Aryl Halides
Source: Molecules. 2026 Apr 23;31(9):1400. doi: 10.3390/molecules31091400 (PMC13165051; doi:10.3390/molecules31091400)
Supplement: Supplementary file 1 [file molecules-31-01400-s001.zip › molecules-4237622-supplementary.pdf]

Supporting Information for:

*C*5-alkyl and *C*5-aryl Substituted 5-Deazaflavin as Sensitizers for Photodehalogenation of Aryl Halides

Huimin Guo[1,*], Xing Guan[1], Heping Li[1], Weihua Guo[1]

[1]School of Chemistry, State Key Laboratory of Fine Chemicals, Dalian University of Technology, Dalian, 116024, P. R. China.

Corresponding author, email: guohm@dlut.edu.cn (H.G.)

Contents

1. General Information······S2

2. Synthesis and Molecular Structure Characterization Data······S5

3. NMR and MS spectra······S10

4. Electrochemical measurements······S17

5. GC spectra······S18

6. DFT/TD-DFT Results······S73

7. References······S91

This file includes 135 Figures and 5 Tables within 91 pages.

KEYWORDS:

Aryl Halides; Deazaflavin; Flavin; Photodehalogenation; Photosensitizer

1. General Information

All the chemicals used in synthesis were of analytical purity and were used as received. Solvents were dried and distilled before used for synthesis.

Analytical Measurements. All chemicals are analytically pure and used as received. NMR spectra were recorded on a NEO 400 MHz spectrometer with $CDCl_3$, as solvents and tetramethylsilane (TMS) as standard at 0.00 ppm. MS were measured with Ultraflextreme MALDI TOF mass spectrometer and Agilent G6230B.

Spectroscopic Measurements. Absorption spectra were recorded on an UV2550 UV–Vis spectrophotometer (Shimadzu, Japan). Fluorescence spectra were measured on an FS5 spectrophotometer (Edinburgh Instruments, UK). Fluorescence lifetimes were measured with an OB920 luminescence lifetime spectrometer (Edinburgh Instruments, UK). The nanosecond transient absorption spectra were measured on LP920 laser flash photolysis spectrometer (Edinburgh Instruments Ltd., UK). Luminescence Quantum Yield were measured with UV2550 UV–Vis spectrophotometer (Shimadzu, Japan) and FS5 spectrophotometer (Edinburgh Instruments, UK). The reaction yield was determined by gas chromatography and measured on GC-MS-6890N/5975B (Agilent, America) and GC-MS-7000B (Agilent, America). The capillary column for GC-MS adopts 100% bonded dimethylpolysiloxane as the stationary phase.

Preparation of Sample Solution for Spectroscopic Measurements. The compound was dissolved in acetonitrile to get 5 mL solution (1.0×10^{-3} M).

Singlet Oxygen Quantum Yield (Φ_Δ). 1,3-Diphenylisobenzofuran (DPBF) was used as $^1O_2$ scavenger and the $^1O_2$ production was monitored by following the absorbance of DPBF at 414 nm. A comparative method was used and was calculated according to the following equation (1) to determine the singlet oxygen quantum yield.

$$\Phi_{\Delta,\mathrm{nuk}} = \Phi_{\Delta,\mathrm{std}}(\frac{A_{\mathrm{std}}}{A_{\mathrm{unk}}})(\frac{I_{\mathrm{unk}}}{I_{\mathrm{std}}})(\frac{\eta_{\mathrm{unk}}}{\eta_{\mathrm{std}}})^2 \qquad (1)$$

In the above equation, *unk* and *std* indicate the unknown sample and the standard, respectively. Φ, A, m, and η represent the singlet oxygen quantum yield, absorbance at excitation wavelength, slope of the absorbance of DPBF changing over time, and refractive index of the solvent used for measurement, respectively. Optically matched solutions were used (the solutions of the sample and the standard should give the same absorbance at the excitation wavelength). Tripyridine ruthenium was used as standard (Φ_Δ = 0.57 in DCM).

Electrochemical Measurements. The measurements were performed in N_2-saturated solution. The cyclic voltammograms were recorded with the CHI610D electrochemical workstation (CHI instruments, Inc., Shanghai, China). The electrochemistry cell includes three electrodes: working electrode, reference electrode and auxiliary electrode. Glassy carbon electrode was used as the

working electrode, Ag/AgCl electrode (0.1 M in dichloromethane) was used as the reference electrode, and the counter electrode was a Pt electrode. Ferrocene was added to the solution as internal standard. Supporting electrolyte is tetrabutylammonium hexafluorophosphate. The scan rate was 100 mV/s, 25 ^{o}C.

Photodehalogenation of Aryl Halide. An aryl halide (0.038 mmol), photosensitizer(8 mol%), Cs_2CO_3 (12 mg, 1.0 equiv.) and N,N-diisopropyl-ethylamine (DIPEA, 13 μL, 2.0 equiv.) were dissolved in acetonitrile (3 mL), and N_2 was bubbled through the solution to remove O_2 for an inert atmosphere. The reaction mixture was stirred continuously and reacted under irradiation at 450 nm with a 20 W LED for 18 or 24 hours. The reaction system was analyzed by gas chromatography (GC).

2. **Synthesis and Molecular Structure Characterization Data**

Synthesis of N^1-butyl-N^3,N^3-diphenylbenzene-1,3-diamine (Compound a):

2 mL of n-Butylamine (approximately 0.02 mol), 5 mL of DMAC, and 0.65 g of 3-bromo-N,N-diphenylaniline (approximately 0.002 mol) are mixed and refluxed at 140°C for 2 hours. Heating is then stopped, and the mixture is allowed to cool to room temperature. 1 mol/L HCl solution is added to adjust the pH. The mixture is washed with a large amount of water, followed by several extractions with small amounts of DCM, and the organic phase is washed 2-3 times with saturated NaCl solution. The organic phase is then dried over anhydrous sodium sulfate, and the solvent is evaporated under reduced pressure to obtain the crude product of compound a.

The crude product is purified by column chromatography, using a elution solvent ratio of PE:EA = 50:1. The pure product of compound a is obtained as a pale yellow oily liquid after rotary evaporation.

Synthesis of 6-(butyl(3-(diphenylamino)phenyl)amino) pyrimidine-2,4(1H, 3H)-dione (Compound b):

0.85 g of compound b (approximately 0.002 mol) and 5 mL of DMA are refluxed at 180°C for 4 hours, then cooled to 100°C. 1 mol/L HCl solution is added to adjust the pH. The mixture is washed with a large amount of water, followed by several extractions with small amounts of DCM to obtain a turbid yellow organic phase. The organic phase is washed 2–3 times with saturated NaCl solution, dried over anhydrous sodium sulfate, and concentrated under reduced pressure to yield

the crude product of compound b.

The crude product is purified by column chromatography using an elution solvent ratio of PE:EA = 2:3. Rotary evaporation yields the pure product of compound b as a pale yellow solid.

^{1}H NMR δ 9.47 (s, 1H), 7.43 (t, *J* = 7.8 Hz, 1H), 7.34 – 7.26 (m, 2H), 7.22 (d, *J* = 8.2 Hz, 4H), 7.16 – 7.08 (m, 2H), 7.08 – 6.99 (m, 1H), 6.70 (d, *J* = 9.9 Hz, 2H), 6.67 (s, 1H), 5.95 (s, 1H), 3.45 (d, *J* = 7.6 Hz, 2H), 3.25 (s, *J* = 3.1 Hz, 3H), 2.06 – 1.99 (m, 1H), 1.66 – 1.59 (m, 1H), 1.08 (t, 3H).

HR-MS(ESI):$[M+H]^{+}$($C_{29}H_{28}N_{4}O_{2}$) calc:440.22, exp:440.2282

Synthesis of 10-butyl-8-(diphenylamino)-5-methylpyrimido [4,5-b]quinoline-2,4(3H,10H)-dione (TPAdFlMe):

0.45 g of compound b (approximately 0.001 mol), 5 mL of acetic anhydride (approximately 0.05 mol), and 5 mL of NMP are refluxed at 140°C for 3 hours. Heating is then stopped, and the mixture is cooled to room temperature. Saturated sodium carbonate solution is added for washing until no bubbles are generated. Water is added for washing, followed by several extractions with small amounts of DCM to obtain the organic phase. The organic phase is washed 2–3 times with saturated NaCl solution, dried over anhydrous sodium sulfate, and concentrated under reduced pressure to yield the crude product of compound **TPAdFlMe**.

The crude product is purified by column chromatography with an elution solvent ratio of PE:EA = 1:2. Rotary evaporation yields the pure product of compound **TPAdFlMe** as a yellow solid.

^{1}H NMR: δ 7.97 (d, *J* = 9.3 Hz, 1H), 7.44 (t, *J* = 7.7 Hz, 4H), 7.32 – 7.26 (m, 7H), 7.08 (d, *J* = 8.9 Hz, 1H), 6.82 (d, *J* = 2.5 Hz, 1H), 3.47 (s, 3H), 3.17 (s, 3H), 1.29 (m, 2H), 1.22 (m, 2H), 0.82 (t, 3H). ^{13}C NMR (151 MHz, Chloroform-*d*) δ 163.01, 154.87, 152.52, 144.15, 139.86, 129.13, 125.86, 125.78, 125.48, 125.25, 115.95, 115.47, 108.19, 43.63, 29.33, 27.90, 21.67, 18.82, 13.10, 13.03.

HR-MS(ESI):$[M+H]^+$($C_{29}H_{28}N_4O_2$) calc:464.22, exp:464.2284

Synthesis of 10-butyl-8-(diphenylamino)-5-(trifluoromethyl)pyrimido[4,5-b]quinoline-2,4(3H,10H)-dione (TPAdFlTF)：

0.45 g of compound b (approximately 0.001 mol), 1 mL of trifluoroacetic anhydride (approximately 0.007 mol), and 5 mL of NMP are refluxed at 140°C for 4 hours. Heating is then stopped, and the mixture is cooled to room temperature. Saturated sodium carbonate solution is added for washing until no bubbles are generated. Water is added for washing, followed by several extractions with small amounts of DCM to obtain the organic phase. The organic phase is washed 2–3 times with saturated NaCl solution, dried over anhydrous sodium sulfate, and concentrated under reduced pressure to yield the crude product of compound **TPAdFlTF**.

The crude product is purified by column chromatography with an elution solvent ratio of PE:EA = 1:1. Rotary evaporation yields the pure product of compound **TPAdFlTF** as a red solid.

^{1}H NMR: δ 7.98 (dd, *J* = 9.6, 2.3 Hz, 1H), 7.48 (t, *J* = 7.7 Hz, 3H), 7.40 – 7.27 (m, 6H), 7.19 – 7.04 (m, 1H), 6.79 (d, *J* = 2.3 Hz, 1H), 3.45 (s, 3H), 1.25 (m, 2H), 1.15 (m,

2H), 0.79 (t, 3H). ^{13}C NMR (151 MHz, Chloroform-*d*) δ 160.83, 157.03, 155.01, 153.74, 144.40, 142.21, 139.95, 139.73, 130.28, 129.52, 129.32, 129.28, 127.01, 126.98, 125.31, 123.37, 121.52, 116.89, 113.22, 110.53, 101.71, 45.59, 32.75, 31.93, 30.04, 28.46, 28.37, 19.84, 13.50.

HR-MS(ESI):[M+H]$^+$($C_{29}H_{25}F_3N_4O_2$) calc:518.19, exp:518.1986

Synthesis of 10-butyl-8-(diphenylamino)-5-phenylpyrimido[4,5-b]quino - line 2,4(3H,10H)-dione (TPAdFlPh):

0.45 g of compound b (approximately 0.001 mol), 550 mg of benzene-1,2-dicarboxylic anhydride (approximately 0.002 mol), and 5 mL of NMP are refluxed at 140°C for 4 hours. Heating is then stopped, and the mixture is cooled to room temperature. Water is added for washing, followed by saturated sodium carbonate solution, and then washed again with water. The mixture is extracted several times with small amounts of DCM to obtain the organic phase. The organic phase is washed 2–3 times with saturated NaCl solution, dried over anhydrous sodium sulfate, and concentrated under reduced pressure to yield the crude product of compound **TPAdFlPh**.

The crude product is purified by column chromatography with an elution solvent ratio of PE:EA = 2:3. Rotary evaporation yields the pure product of compound **TPAdFlPh** as a yellow solid.

^{1}H NMR: δ 7.51 (dd, *J* = 10.4, 6.9 Hz, 2H), 7.43 (t, *J* = 7.6 Hz, 3H), 7.32 – 7.24 (m, 3H), 7.21 (d, *J* = 7.2 Hz, 1H), 7.11 (d, *J* = 9.1 Hz, 1H), 6.90 – 6.84 (m, 1H), 3.32 (s, 3H), 1.61 (m, 2H),1.29 (m, 2H),1.23 (t, 3H). ^{13}C NMR (151 MHz, Chloroform-*d*) δ

162.20, 156.08, 153.83, 145.00, 141.52, 136.81, 131.72, 130.09, 129.68, 128.20, 128.02, 127.15, 126.85, 126.42, 125.34, 117.26, 116.26, 108.05, 44.73, 29.70, 27.94, 19.90, 13.61.

HR-MS(ESI):[M+H]$^+$($C_{34}H_{30}N_4O_2$) calc:526.24,exp:526.2446

3. NMR and MS spectra

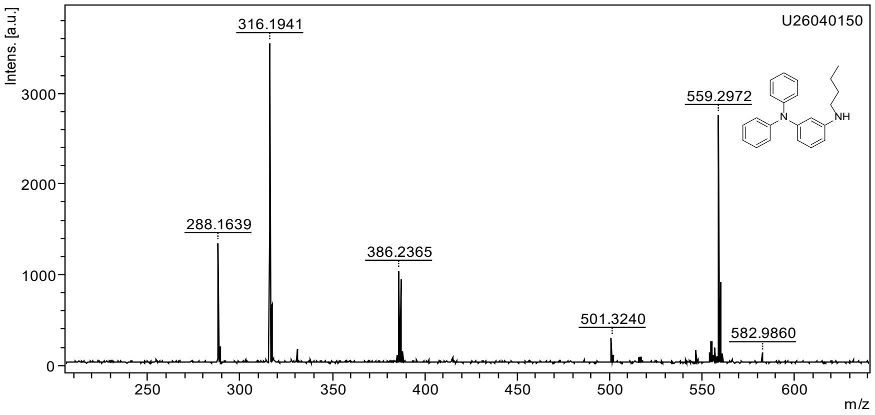

Figure S3-1. MS spectrum of compound a(M $^{+}$)

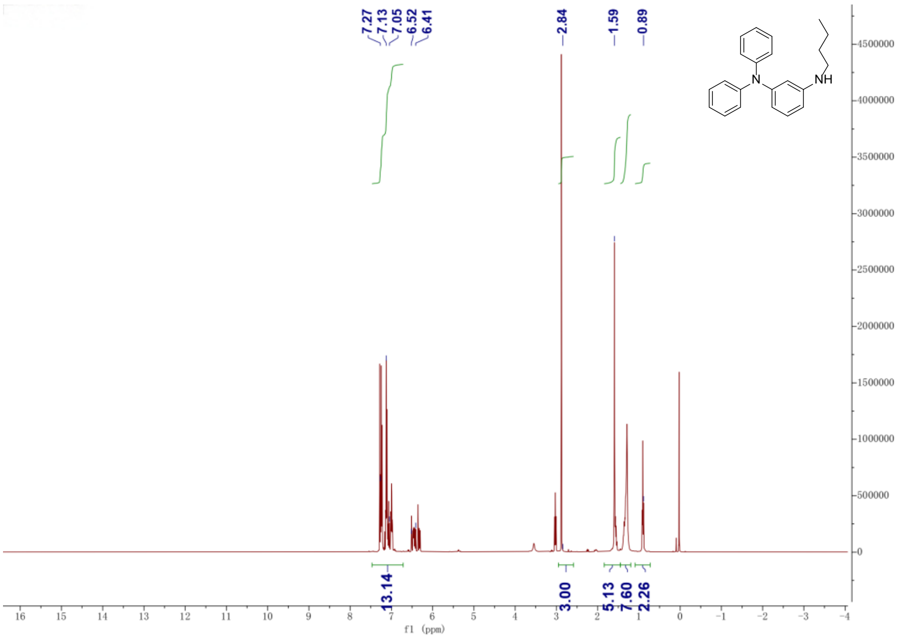

Figure S3-2. ^{1}H NMR spectrum of compound a (400 MHz, $CDCl_3$), 25 °C

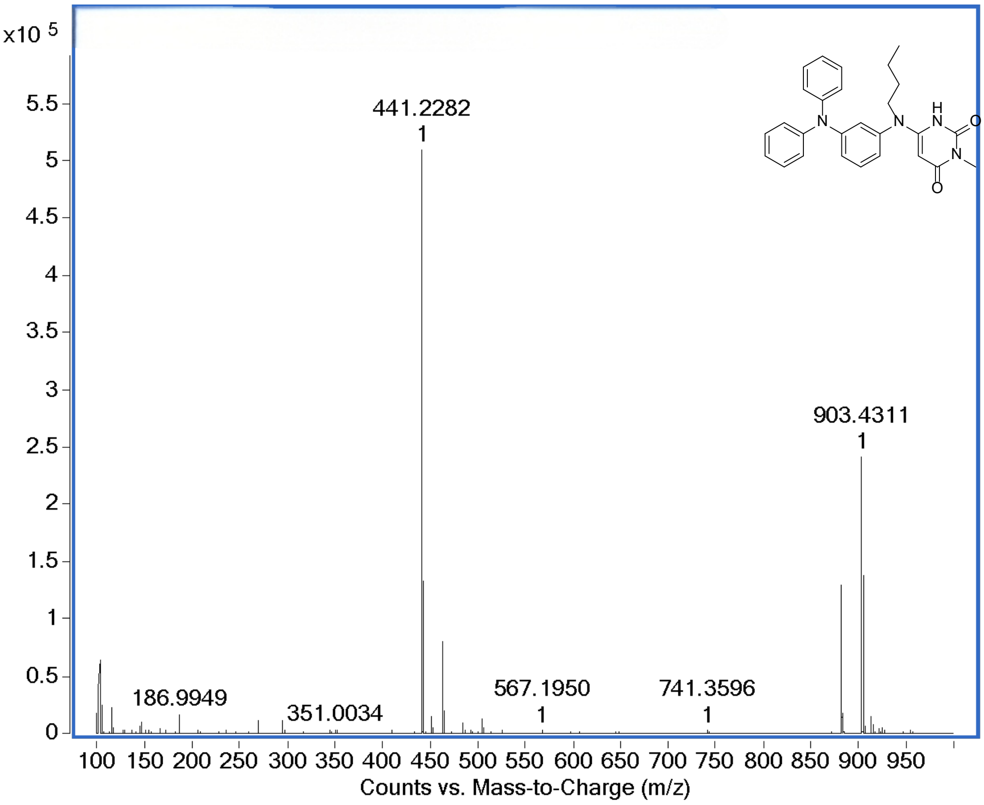

Figure S3-3. MS spectrum of compound b(M+H+).

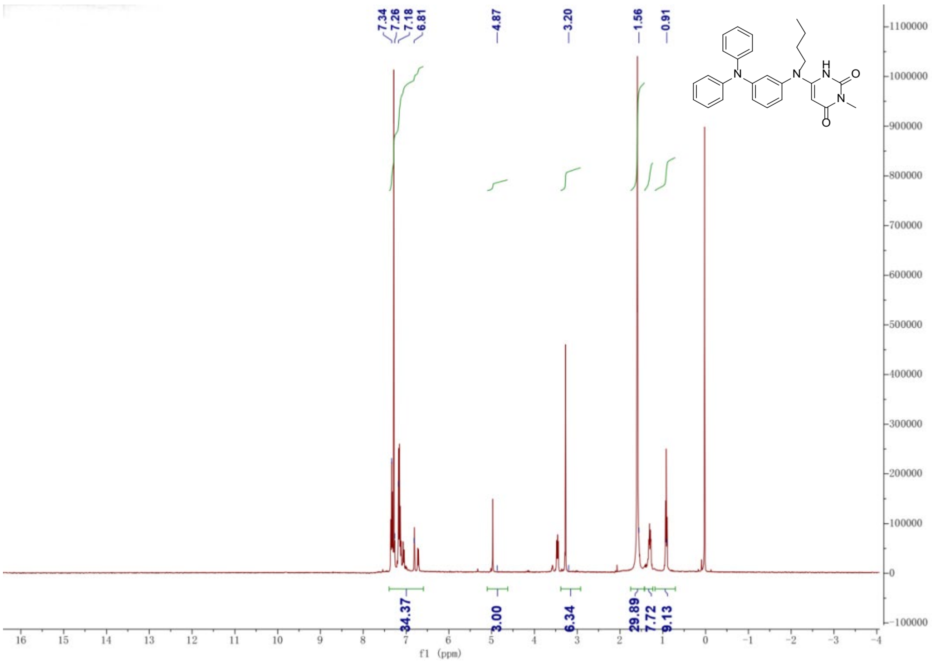

Figure S3-4. ^{1}H NMR spectrum of compound b (400 MHz, $CDCl_3$), 25 °C.

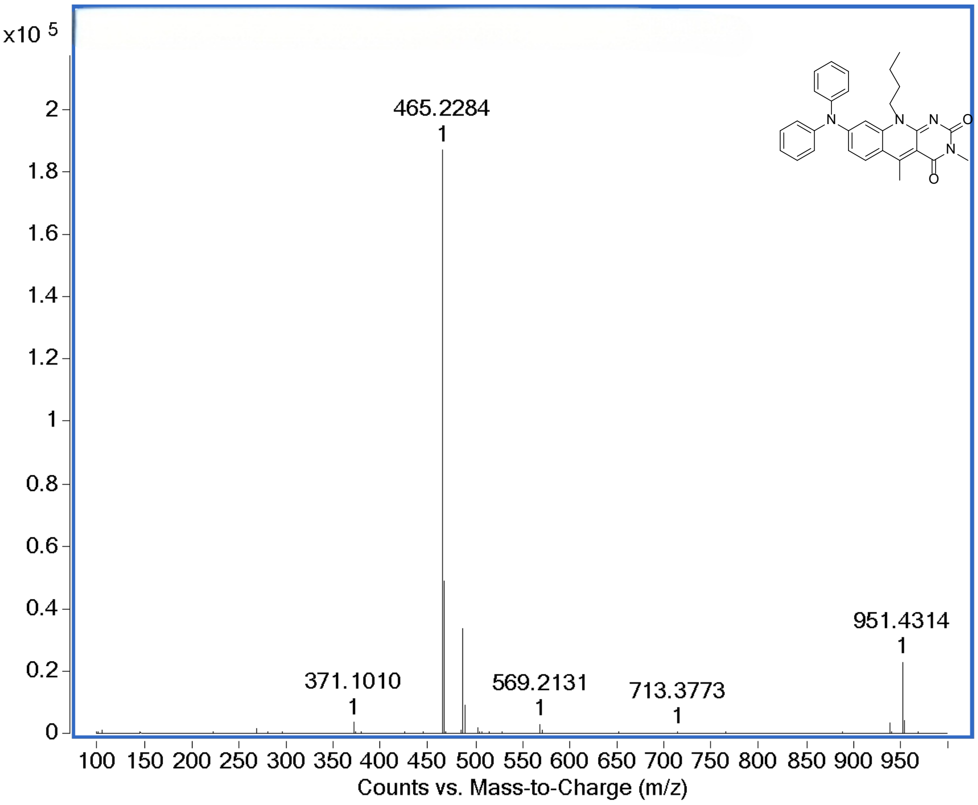

Figure S3-5. MS spectrum of **TPAdFlMe**(M+H+).

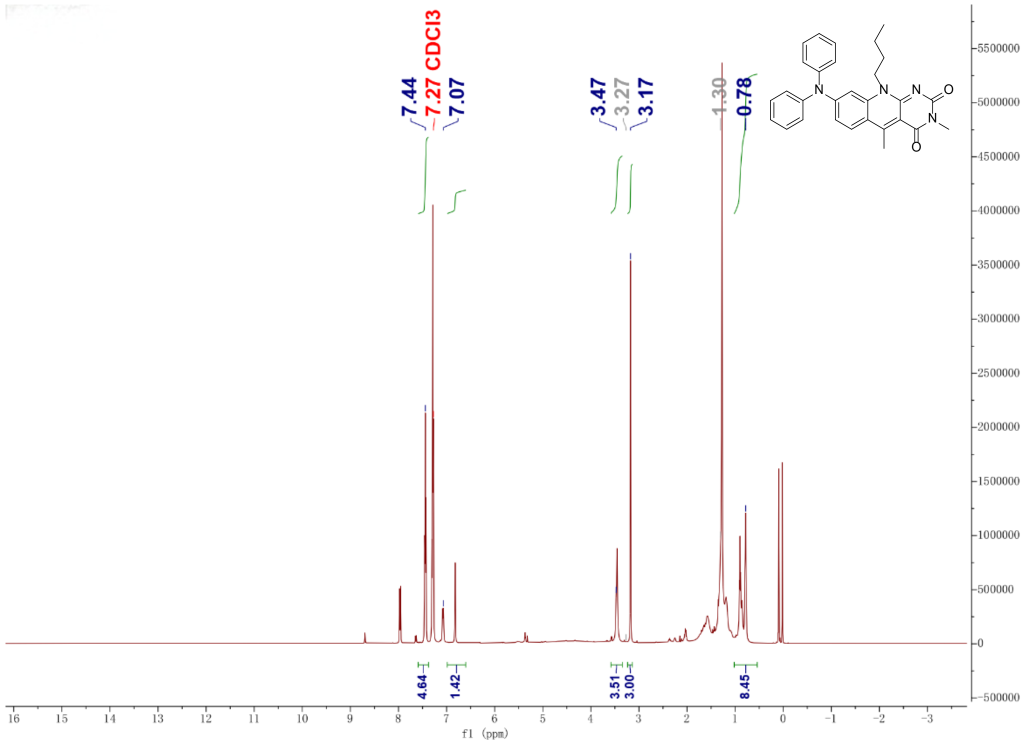

Figure S3-6. ^{1}H NMR spectrum of **TPAdFlMe** (400 MHz, $CDCl_3$), 25 °C.

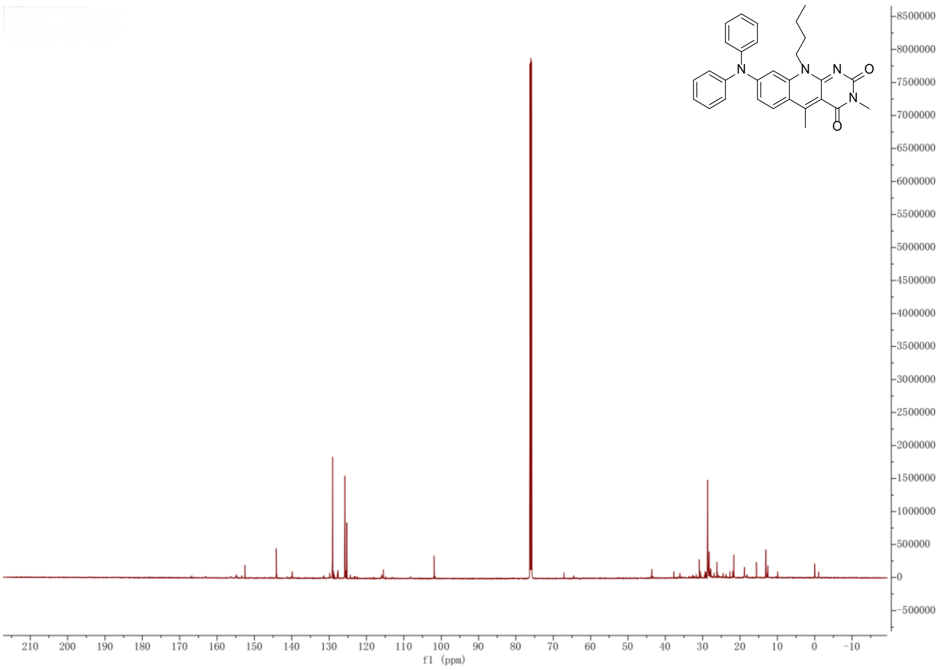

Figure S3-7. ^{13}C NMR spectrum of **TPAdFlMe** (400 MHz, $CDCl_3$), 25 °C.

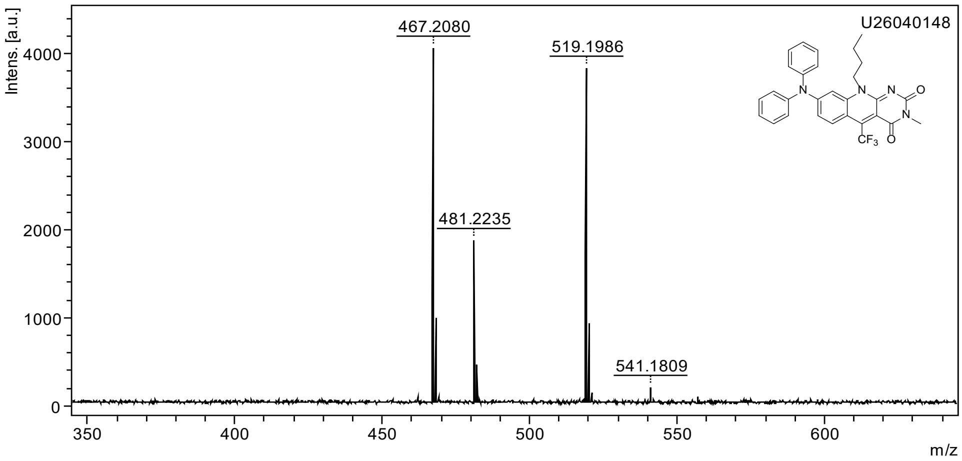

Figure S3-8. MS spectrum of **TPAdFlTF**(M+H+).

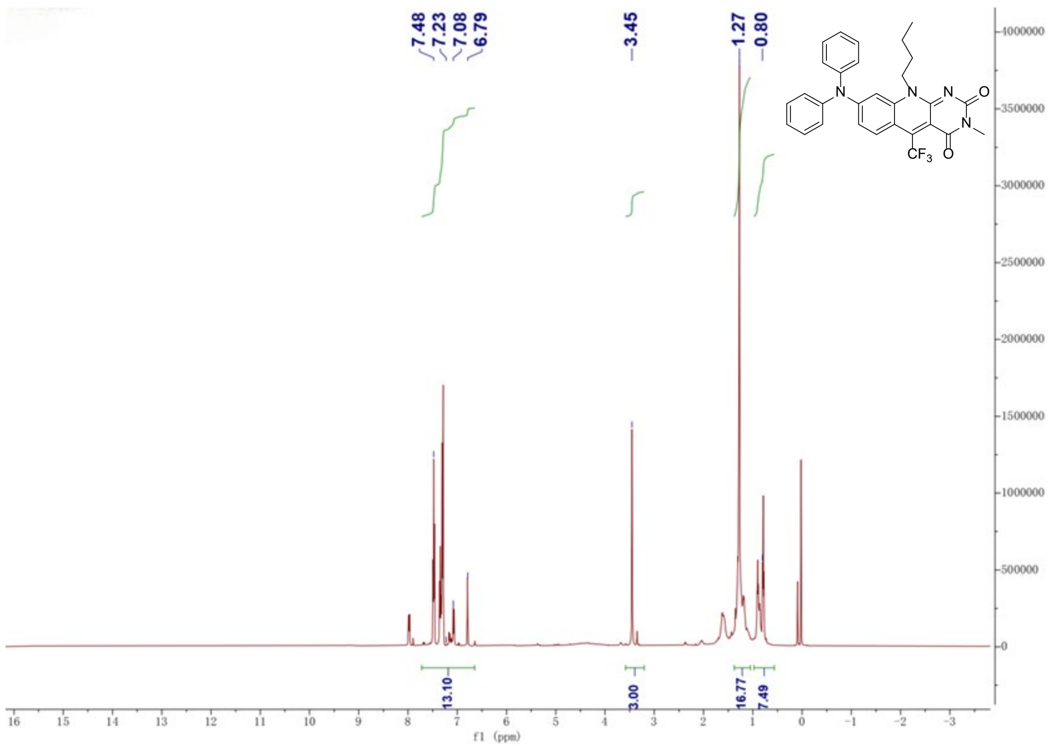

Figure S3-9. ^{1}H NMR spectrum of **TPAdFlTF** (400 MHz, CDCl3), 25 °C.

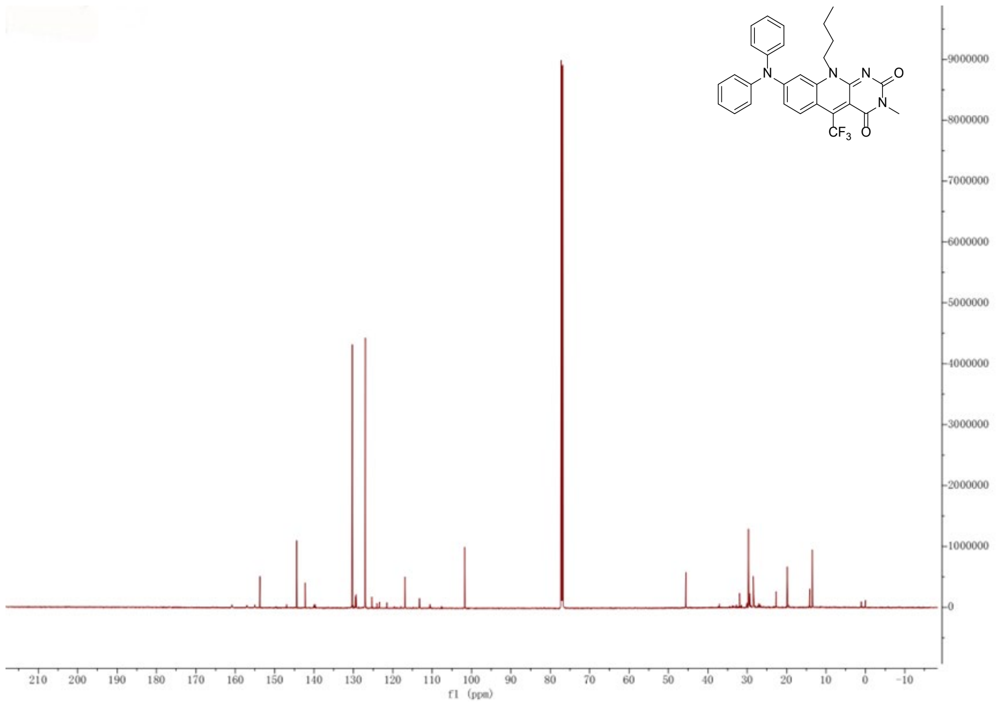

Figure S3-10. ^{13}C NMR spectrum of **TPAdFlTF** (400 MHz, CDCl3), 25 °C.

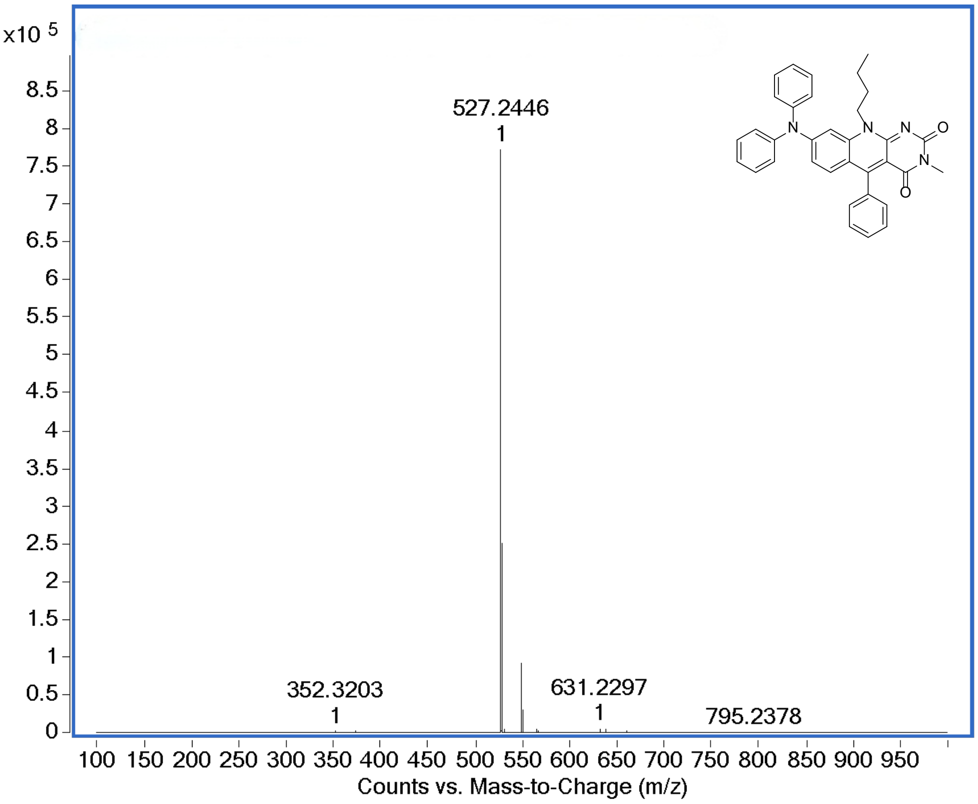

Figure S3-11. MS spectrum of **TPAdFlPh**(M+H+).

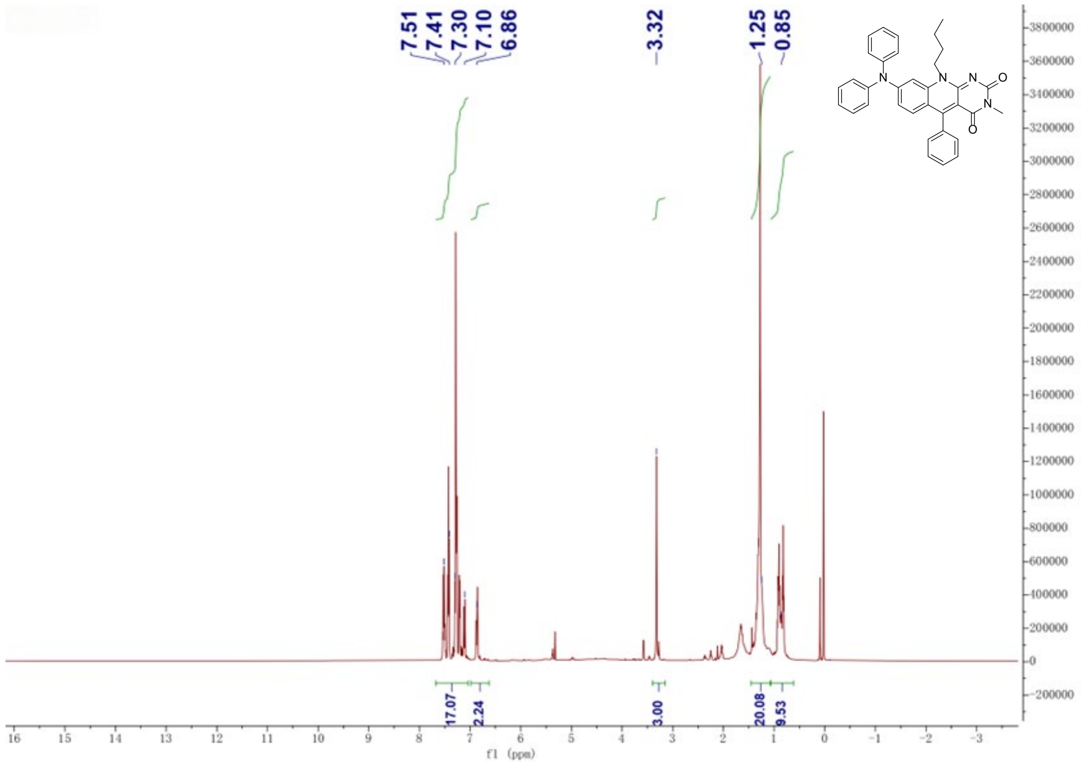

Figure S3-12. ^{1}H NMR spectrum of **TPAdFlPh** (400 MHz, $CDCl_3$), 25 °C.

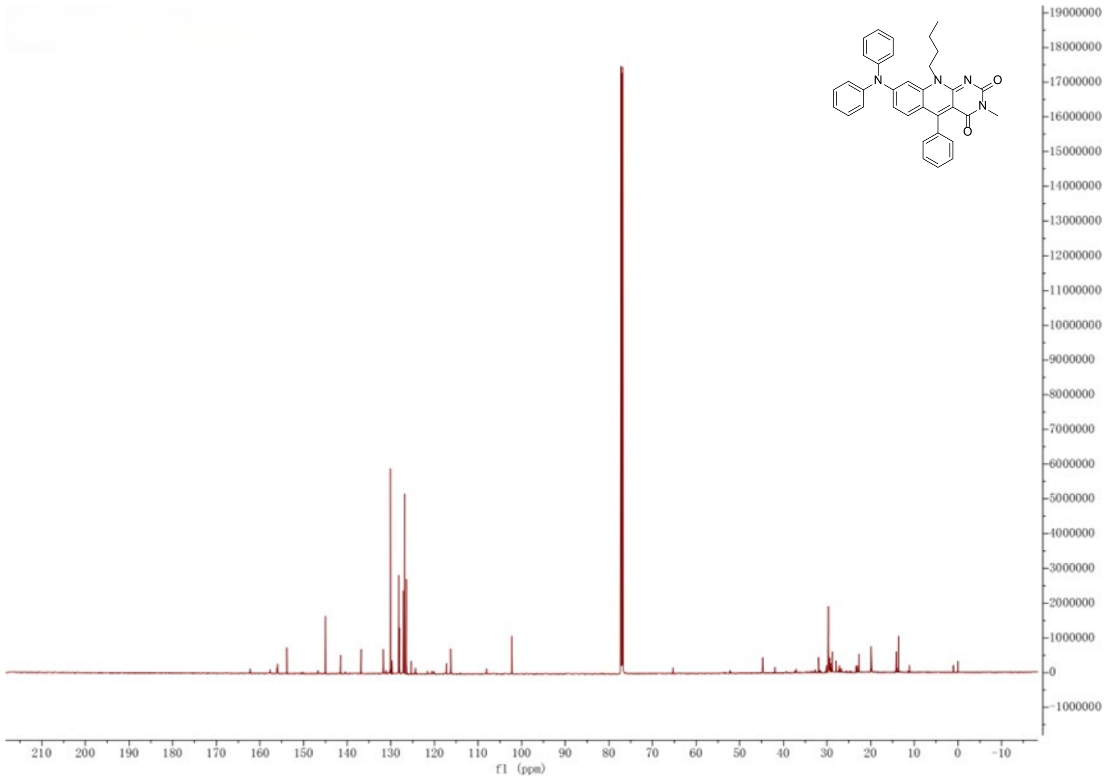

Figure S3-13. ^{13}C NMR spectrum of **TPAdFlPh** (400 MHz, $CDCl_3$), 25 °C.

4. Electrochemical Measurement

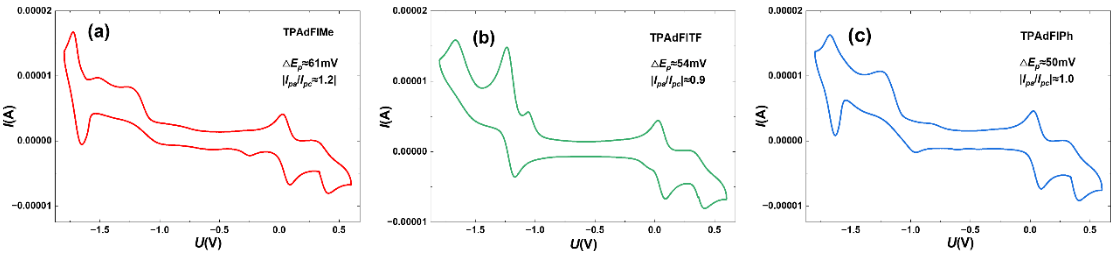

Figure S4 Cyclic voltammetry curves, TPAdFlMe(a), TPAdFlTF(b), TPAdFlPh(c) in dichloromethane(DCM), the scan rate was 100 mV/s, 25 °C.

5. GC Spectra

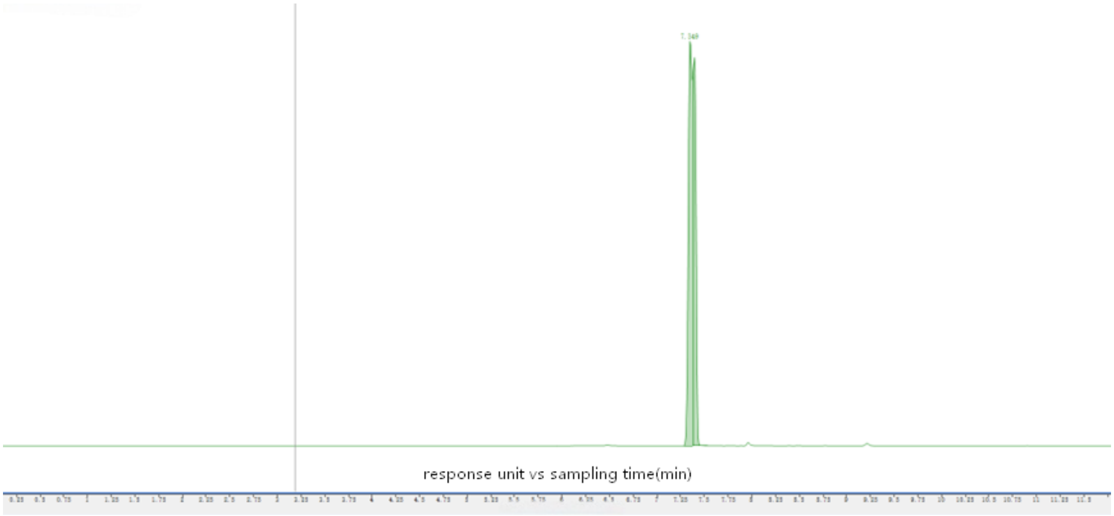

Figure S5-1. TPAdFlMe 8%, DIPEA 0.076 mmol, Cs_2CO_3 0.038 mmol, time 24 h, no benzene is formed.

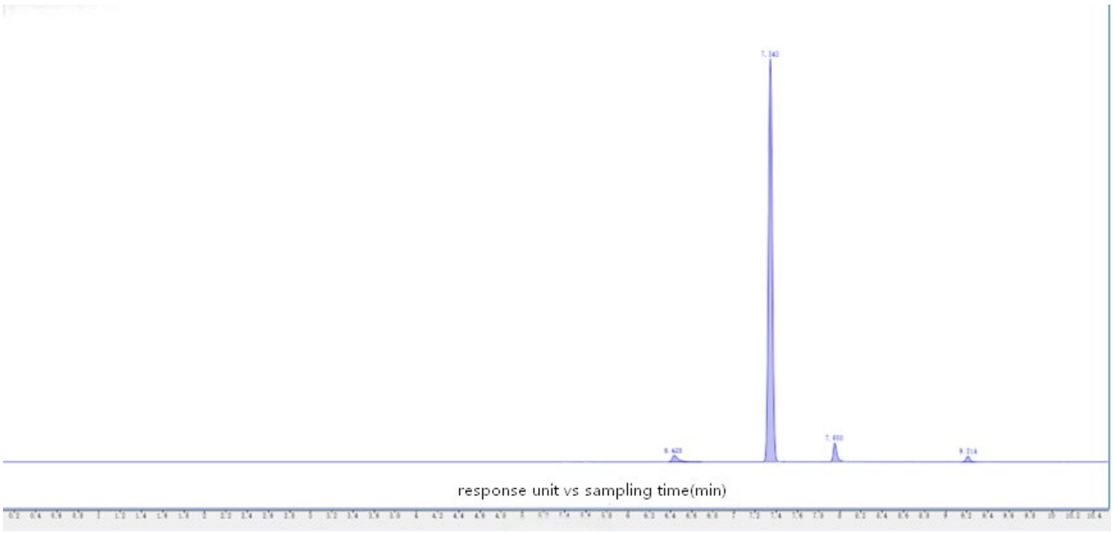

Figure S5-2. TPAdFlMe 8%, DIPEA 0.076 mmol, Cs_2CO_3 0.038 mmol, time 24 h, no benzene is formed.

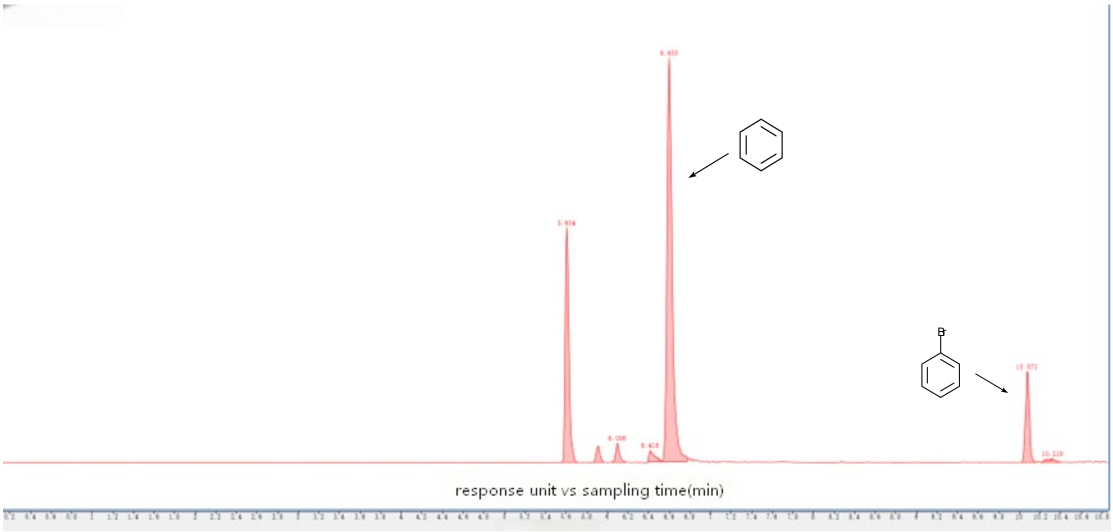

Figure S5-3. TPAdFlMe 8%, DIPEA 0.076 mmol, Cs_2CO_3 0.038 mmol, time 24 h, yield 49%

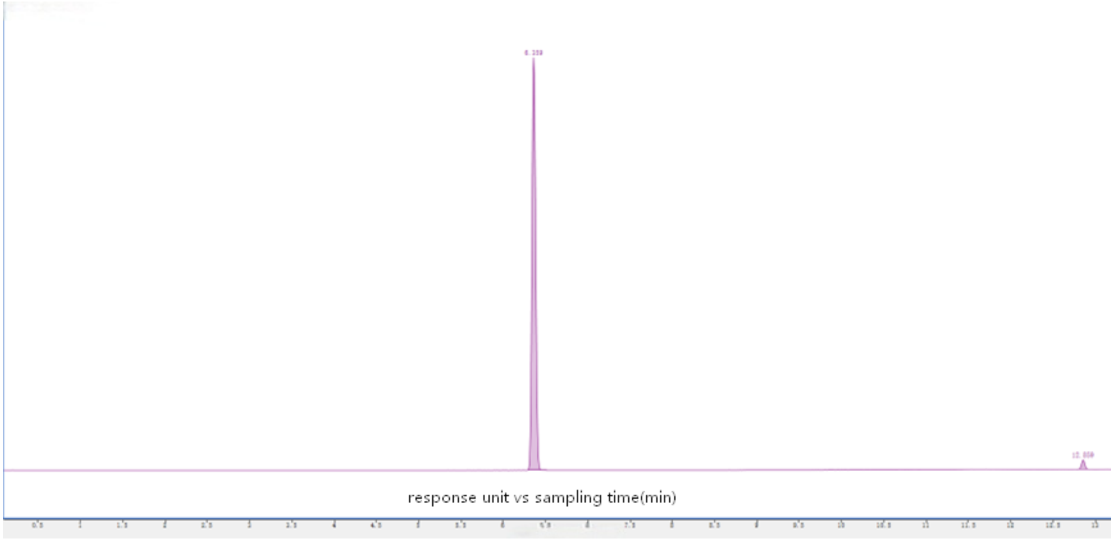

Figure S5-4. TPAdFlMe 8%, DIPEA 0.076 mmol, Cs_2CO_3 0.038 mmol, time 24 h, no benzene is formed.

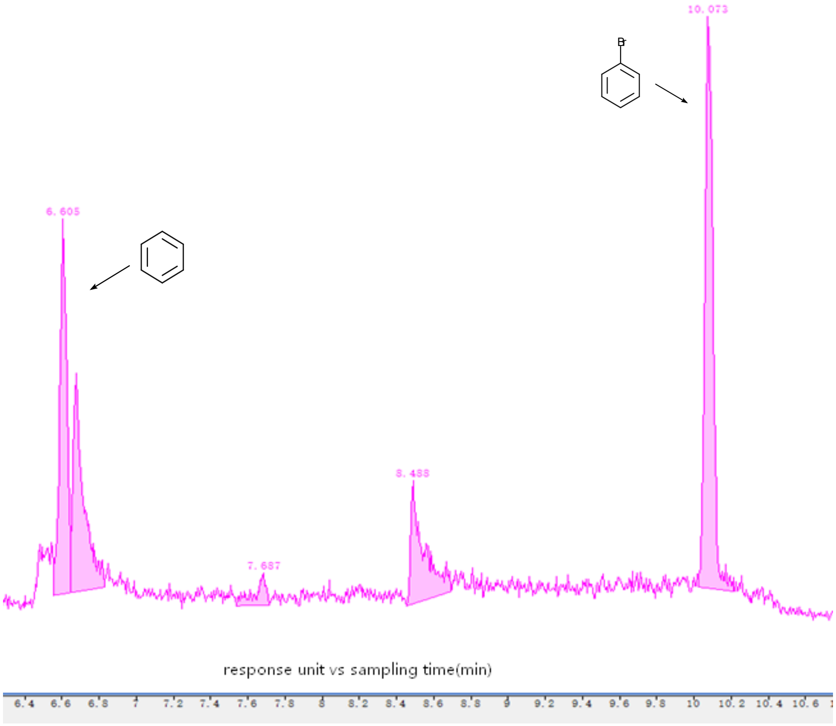

Figure S5-5. TPAdFlMe 8%, DIPEA 0.076 mmol, Cs_2CO_3 0.038 mmol, time 6h, yield 37%.

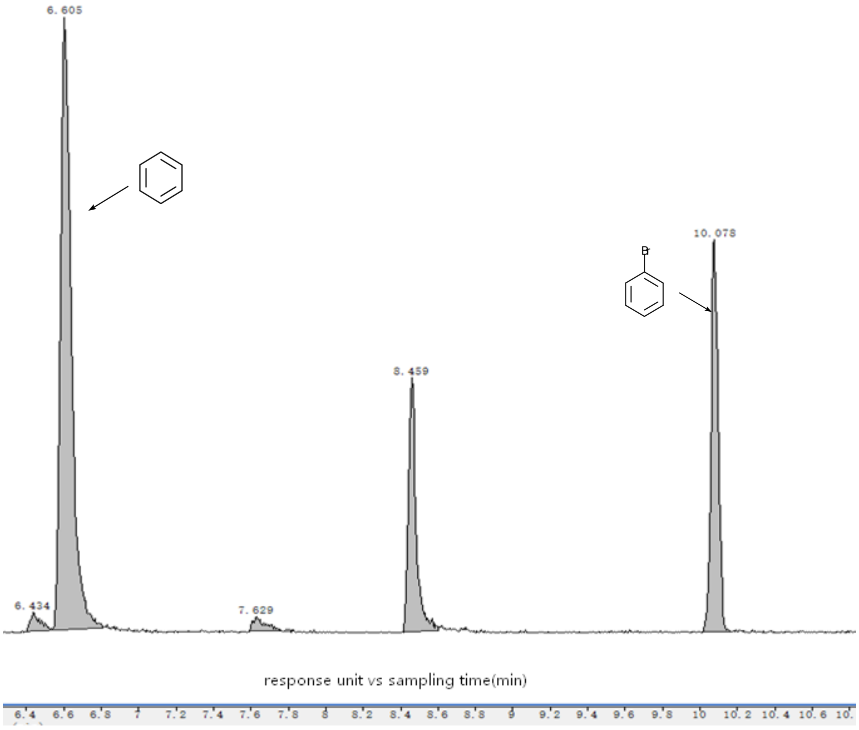

Figure S5-6. TPAdFlMe 8%, DIPEA 0.076 mmol, Cs_2CO_3 0.038 mmol, time 9h, yield 47%.

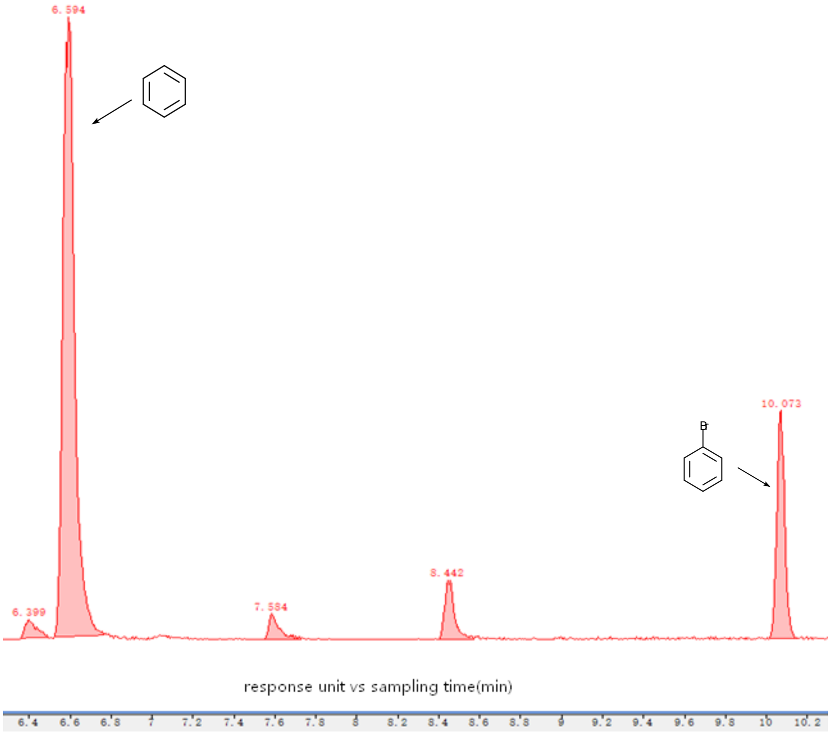

Figure S5-7. TPAdFlMe 8%, DIPEA 0.076 mmol, Cs_2CO_3 0.038 mmol, time 12h, yield 49%

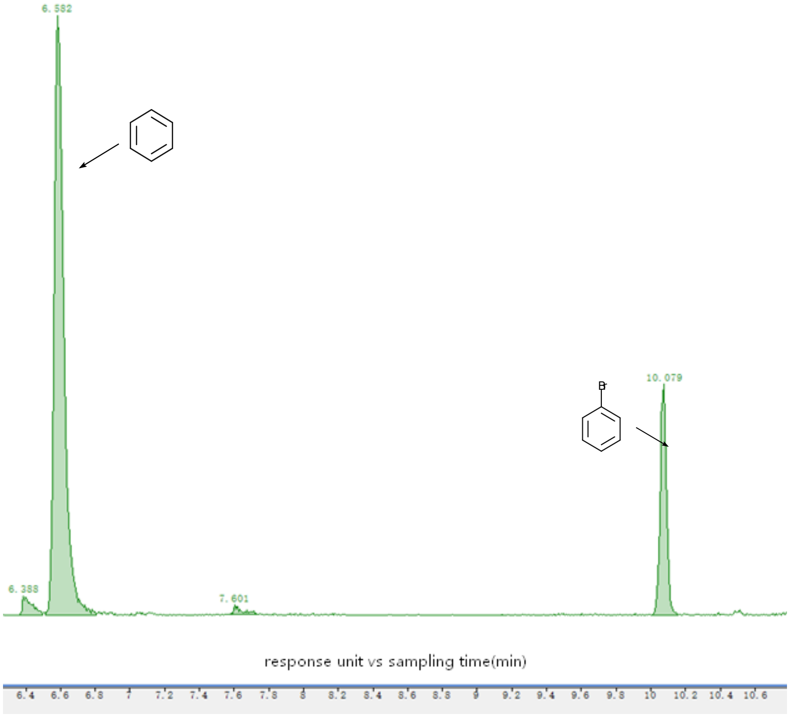

Figure S5-8. TPAdFlMe 8%, DIPEA 0.076 mmol, Cs_2CO_3 0.038 mmol, time 15h, yield 79%

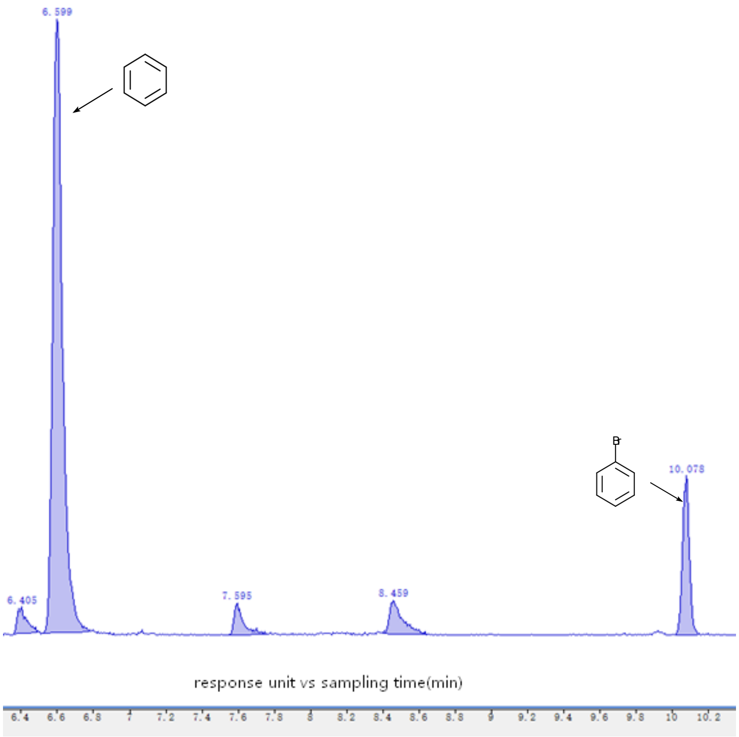

Figure S5-9. TPAdFlMe 8%, DIPEA 0.076 mmol, Cs_2CO_3 0.038 mmol, time 18 h, yield 80%.

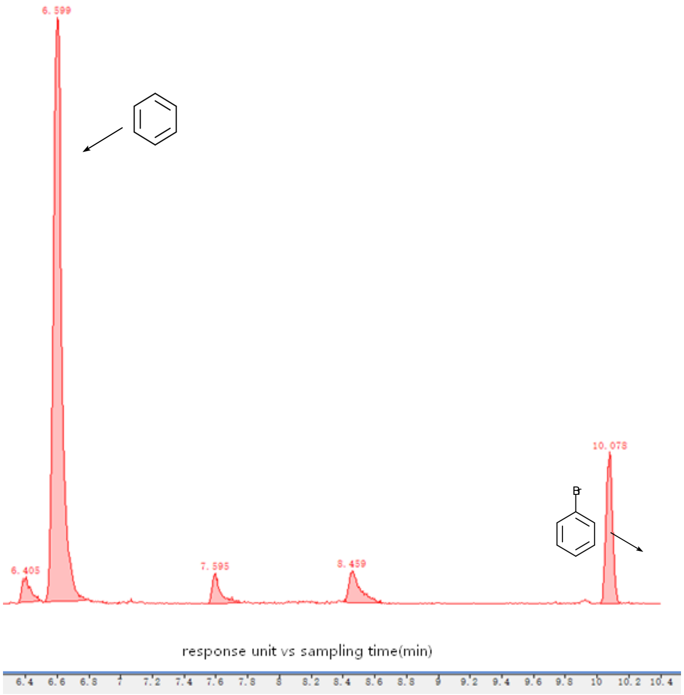

Figure S5-10. TPAdFlMe 8%, DIPEA 0.076 mmol, Cs_2CO_3 0.038 mmol, time 24 h, yield 85%.

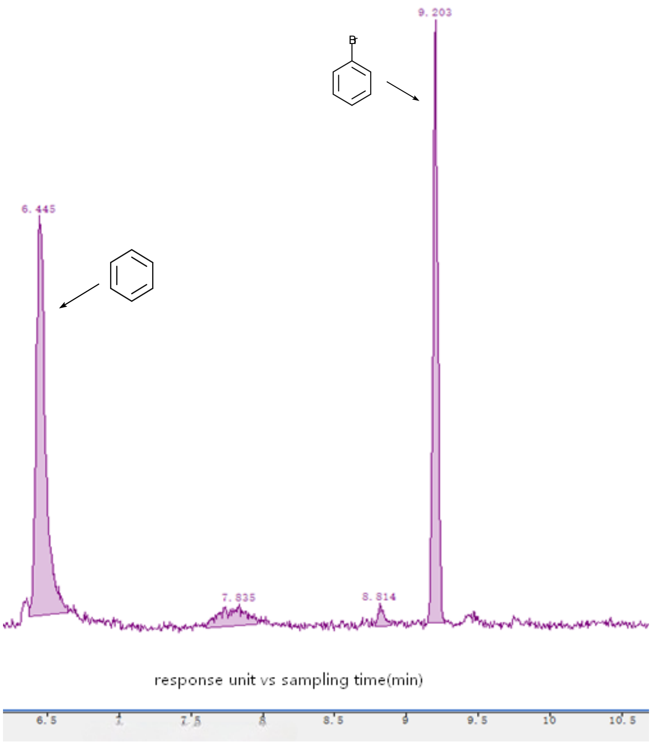

Figure S5-11. TPAdFlPh 8%, DIPEA 0.076 mmol, Cs_2CO_3 0.038 mmol, time 6h, yield 44%.

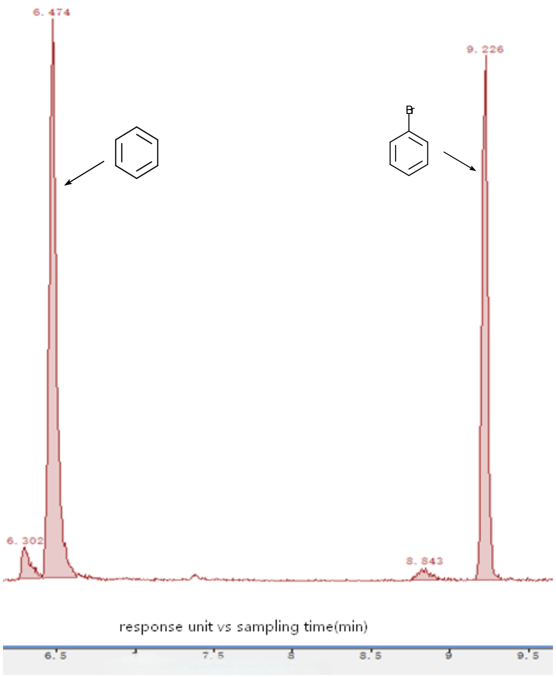

Figure S5-12. TPAdFlPh 8%, DIPEA 0.076 mmol, Cs_2CO_3 0.038 mmol, time 9h, yield 47%.

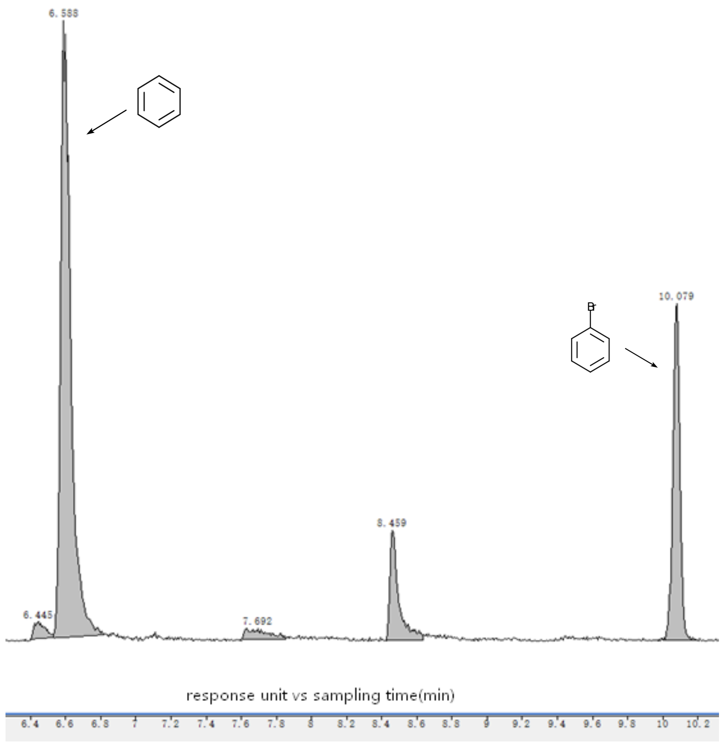

Figure S5-13. TPAdFlPh 8%, DIPEA 0.076 mmol, Cs_2CO_3 0.038 mmol, time 12h, yield 73%.

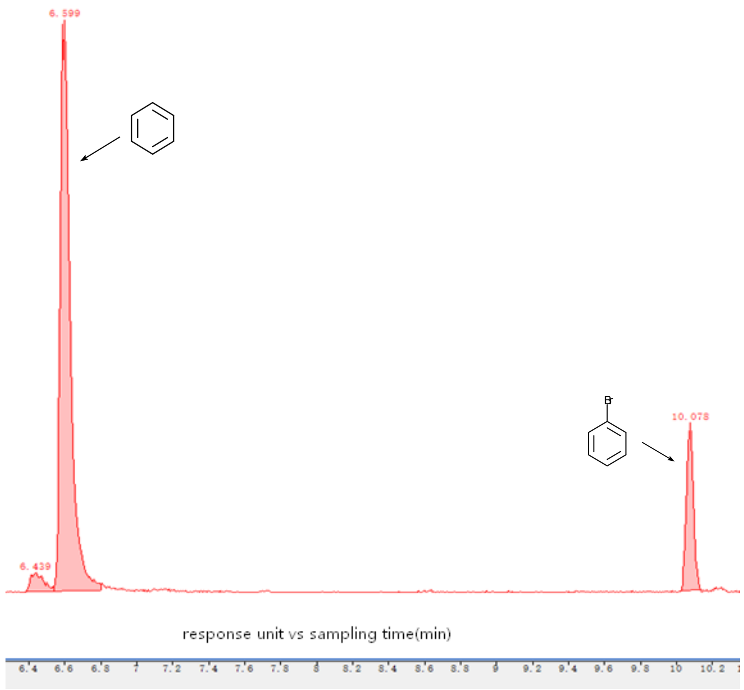

Figure S5-14. TPAdFlPh 8%, DIPEA 0.076 mmol, Cs_2CO_3 0.038 mmol, time 15h, yield 83%.

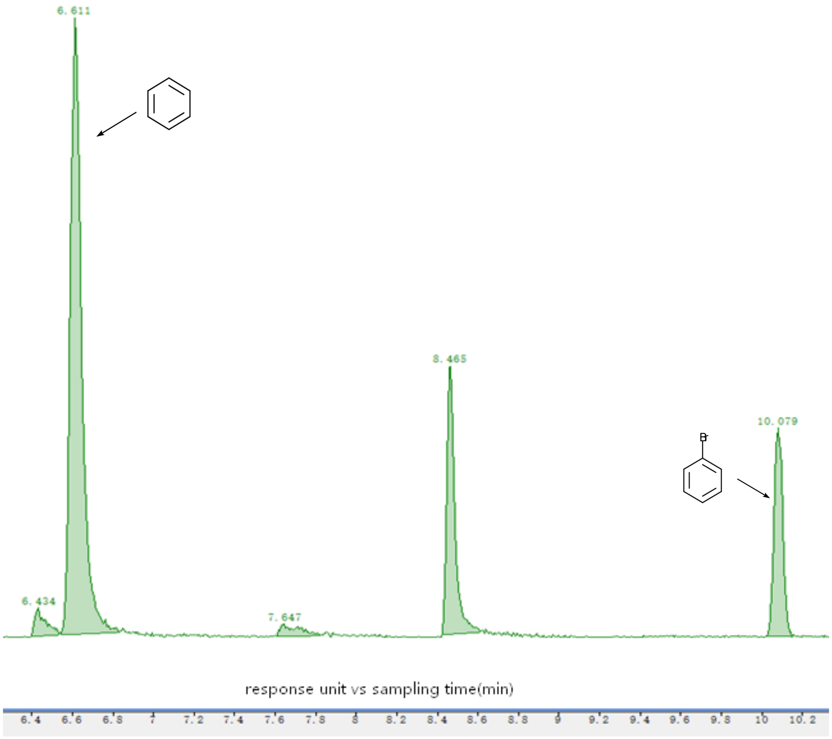

Figure S5-15. TPAdFlPh 8%, DIPEA 0.076 mmol, Cs_2CO_3 0.038 mmol, time 18 h, yield 82%.

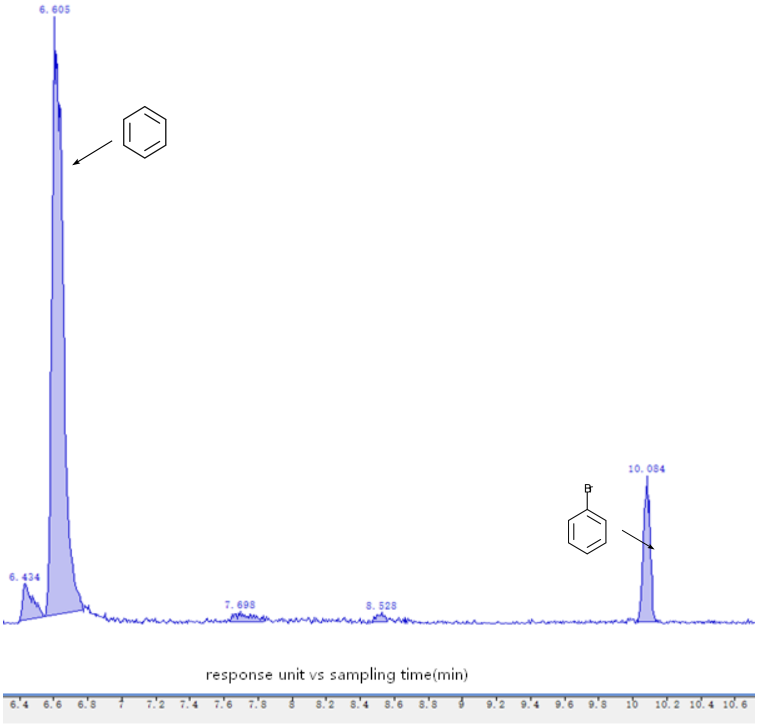

Figure S5-16. TPAdFlPh 8%, DIPEA 0.076 mmol, Cs_2CO_3 0.038 mmol, time 24 h, yield 88%.

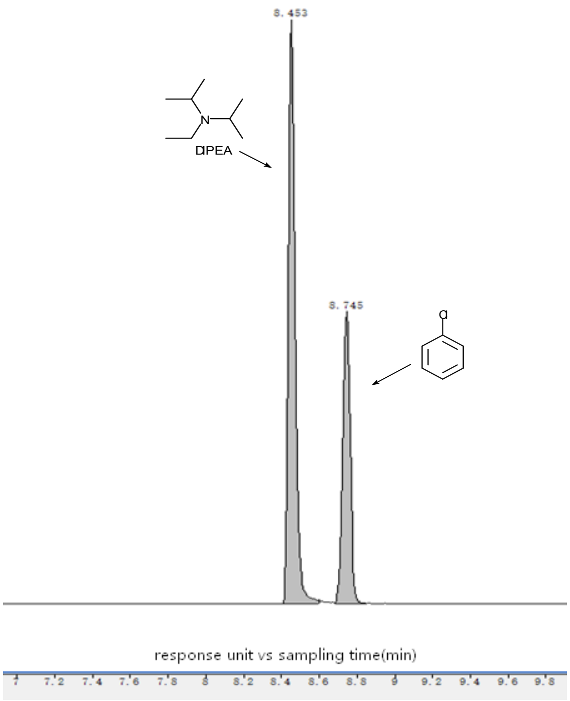

Figure S5-17. TPAdFlMe 0%, DIPEA 0.076 mmol, Cs_2CO_3 0.038 mmol, time 18 h, yield 0%.

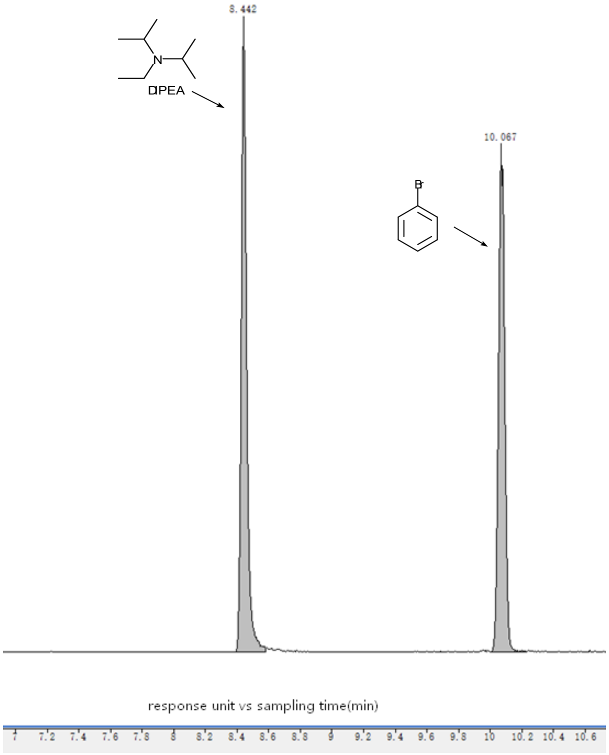

Figure S5-18. TPAdFlMe 0%, DIPEA 0.076 mmol, Cs_2CO_3 0.038 mmol, time 18 h, yield 0%.

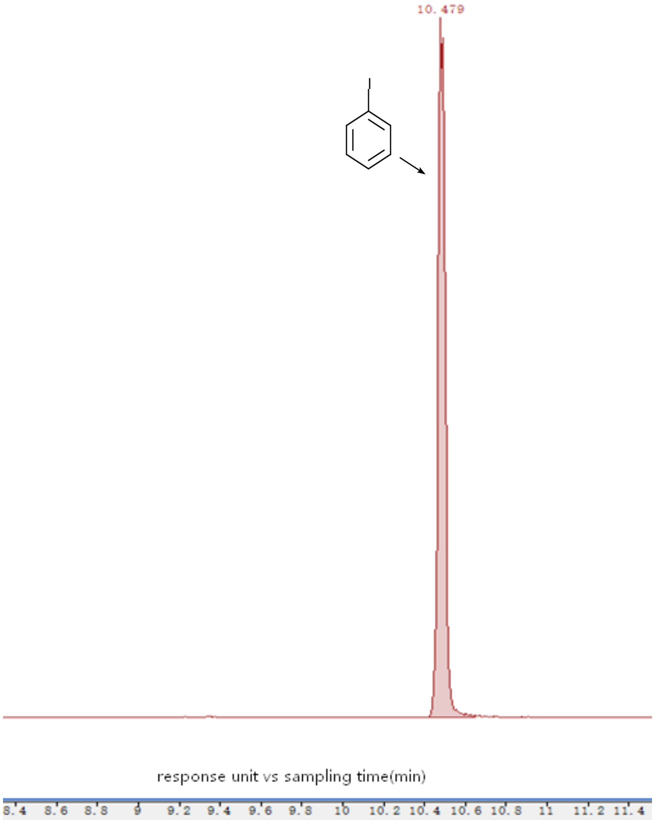

Figure S5-19. TPAdFlMe 0%, DIPEA 0.076 mmol, Cs_2CO_3 0.038 mmol, time 18 h, yield 0%.

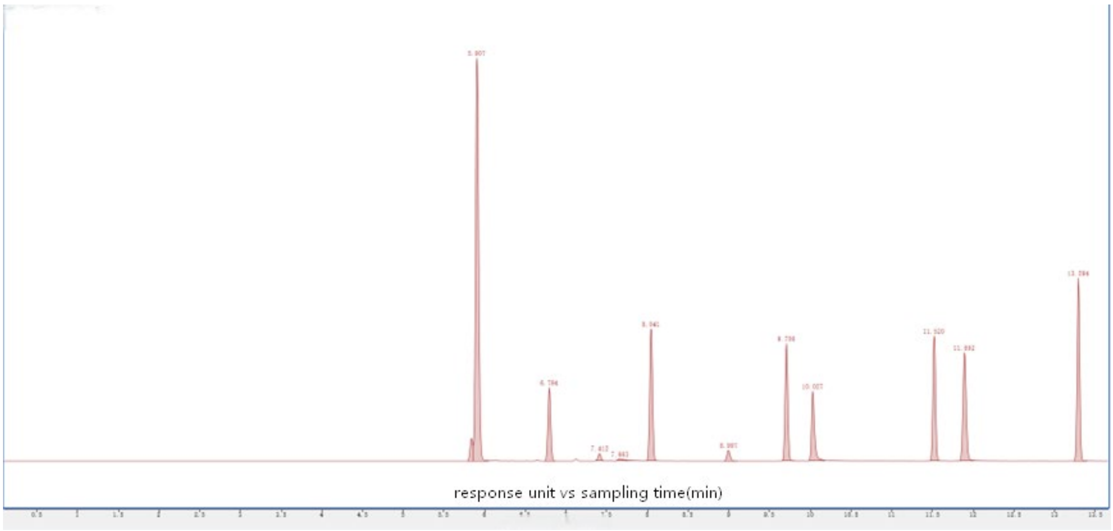

Figure S5-20. Fl 8%, DIPEA 0.076 mmol, Cs_2CO_3 0.038 mmol, time 18 h, no benzene is formed.

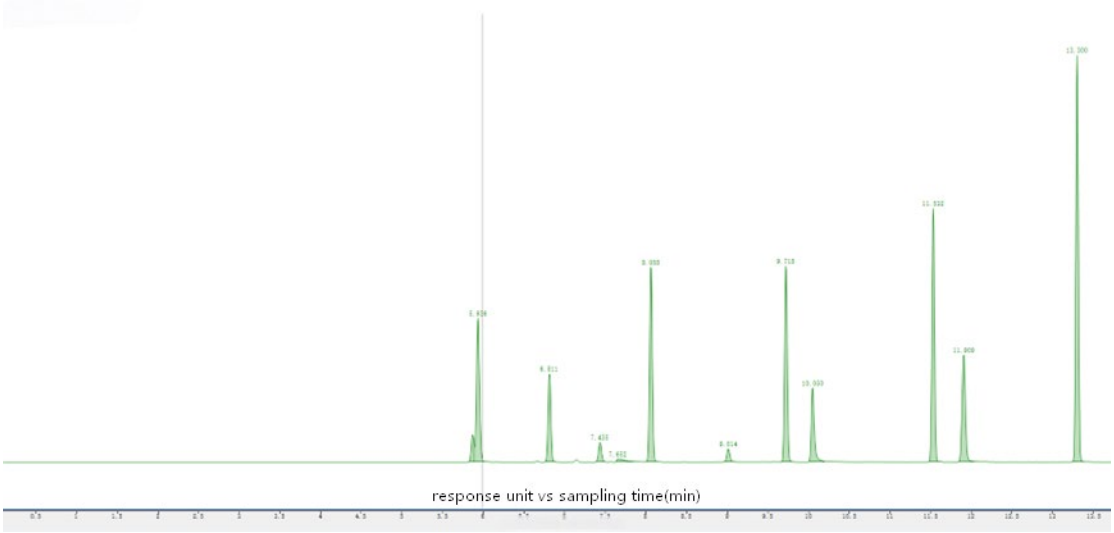

Figure S5-21. RFTA 8%, DIPEA 0.076 mmol, Cs_2CO_3 0.038 mmol, time 18 h, yield no benzene is formed.

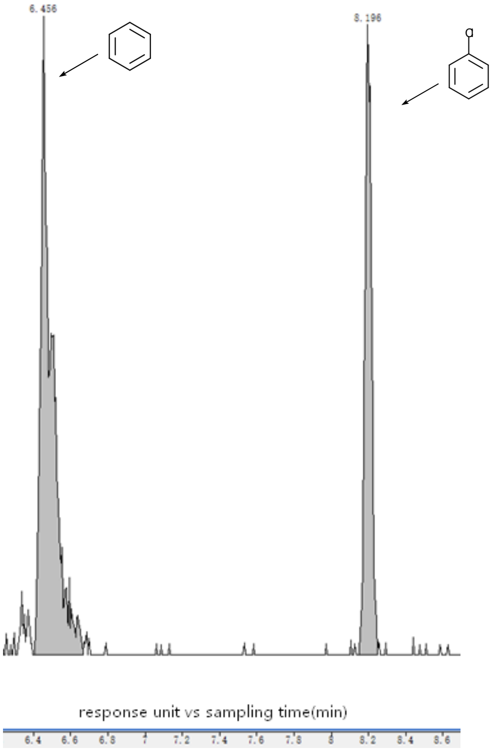

Figure S5-22. TPAdFlMe 0%, DIPEA 0.076 mmol, Cs_2CO_3 0.038 mmol, time 18 h, yield 0%.

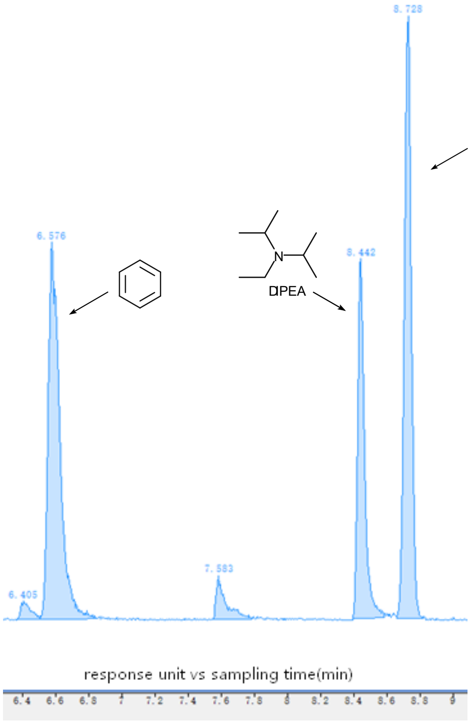

Figure S5-23. TPAdFlPh 8%, DIPEA 0.076 mmol, Cs_2CO_3 0.038 mmol, time 18 h, yield 51%.

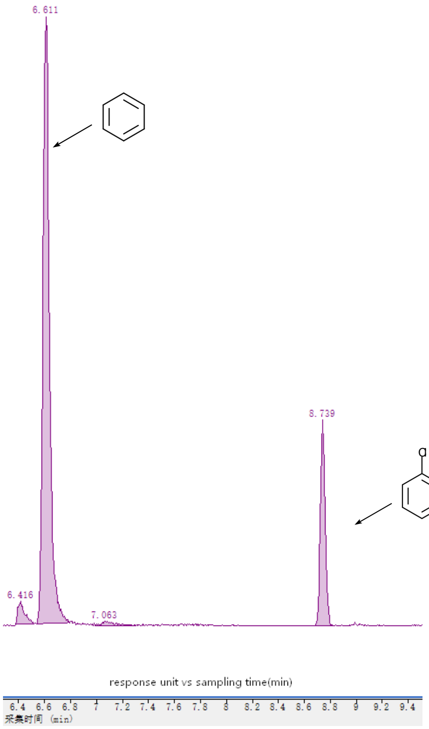

Figure S5-24. TPAdFlMe 8%, DIPEA 0.076 mmol, Cs_2CO_3 0.038 mmol, time 24 h, yield 80%.

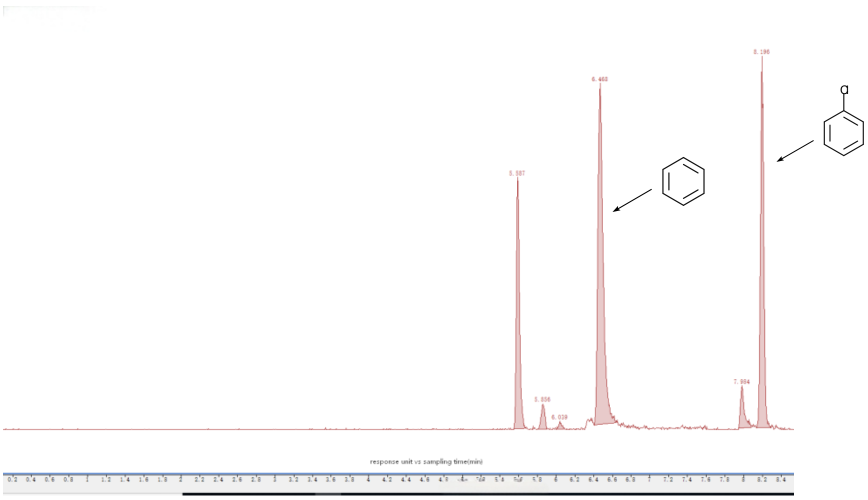

Figure S5-25 TPAdFlPh 8%, DIPEA 0.076 mmol, Cs_2CO_3 0.038 mmol, time 24 h, yield 60%.

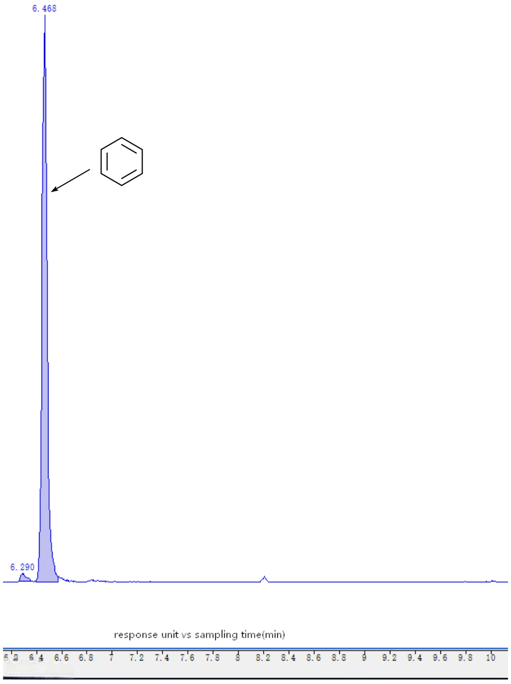

Figure S5-26 TPAdFlMe 8%, DIPEA 0.076 mmol, Cs_2CO_3 0.038 mmol, time 18 h, yield 100%.

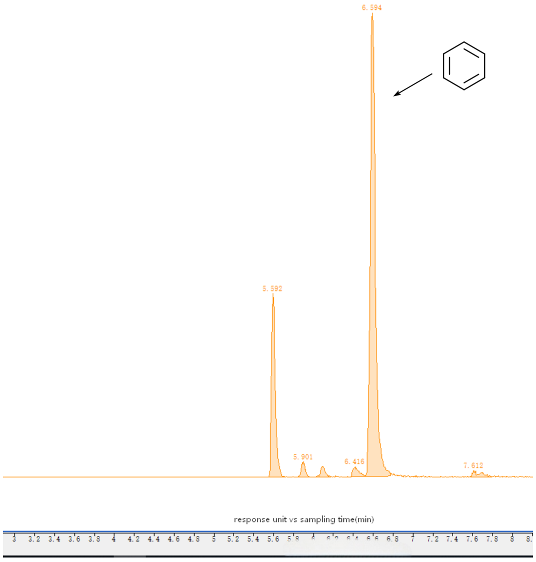

Figure S5-27 TPAdFlPh 8%, DIPEA 0.076 mmol, Cs_2CO_3 0.038 mmol, time 18 h, yield 100%.

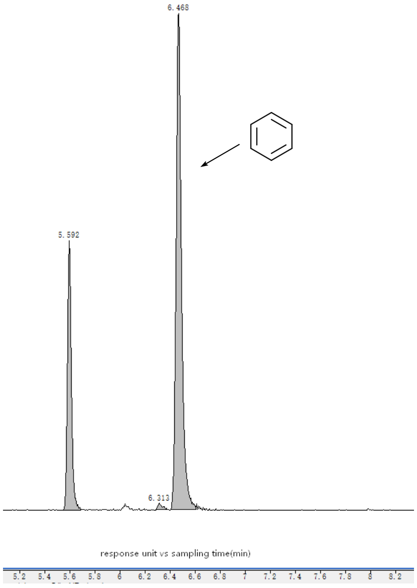

Figure S5-28 TPAdFlMe 8%, DIPEA 0.076 mmol, Cs_2CO_3 0.038 mmol, time 24 h, yield 100%.

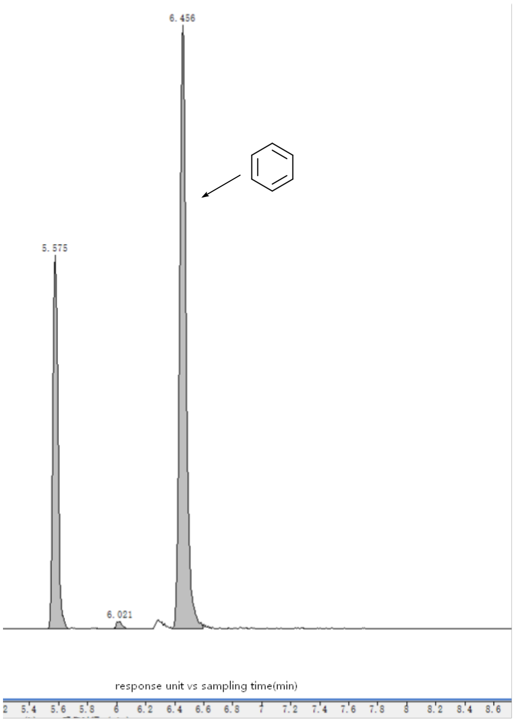

Figure S5-29 TPAdFlPh 8%, DIPEA 0.076 mmol, Cs_2CO_3 0.038 mmol, time 24 h, yield 100%.

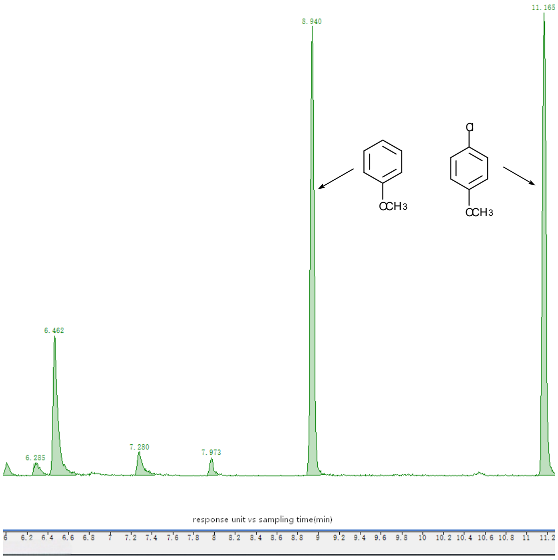

Figure S5-30 TPAdFlMe 8%, DIPEA 0.076 mmol, Cs_2CO_3 0.038 mmol, time 18 h, yield 49%.

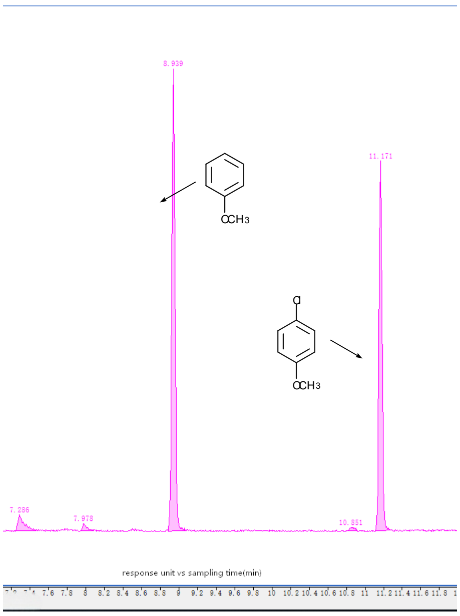

Figure S5-31 TPAdFlPh 8%, DIPEA 0.076 mmol, Cs_2CO_3 0.038 mmol, time 18 h, yield 56%.

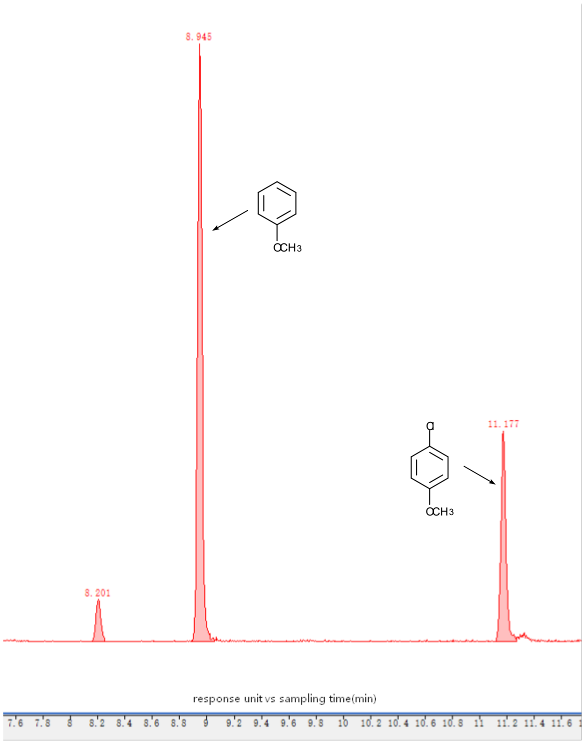

Figure S5-32 TPAdFlMe 8%, DIPEA 0.076 mmol, Cs_2CO_3 0.038 mmol, time 24 h, yield 70%.

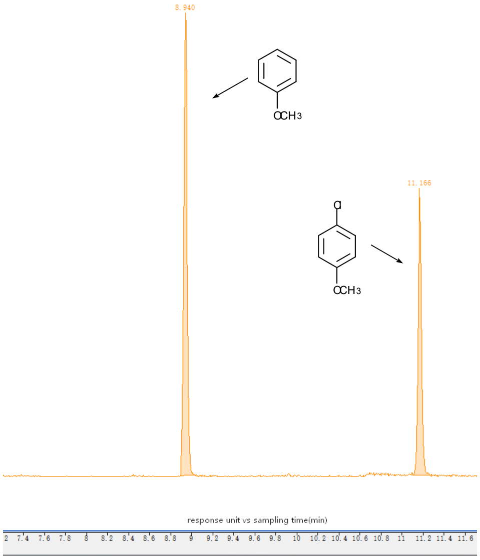

Figure S5-33 TPAdFlPh 8%, DIPEA 0.076 mmol, Cs_2CO_3 0.038 mmol, time 24 h, yield 62%.

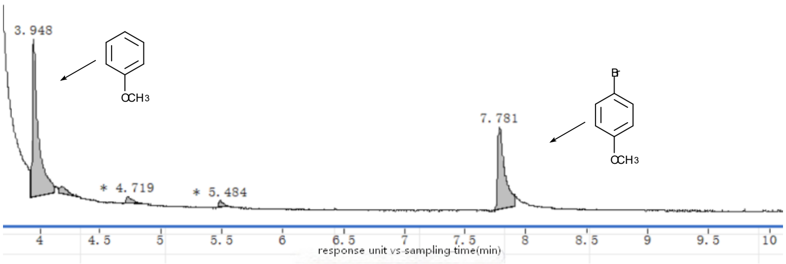

Figure S5-34 TPAdFlMe 8%, DIPEA 0.076 mmol, Cs_2CO_3 0.038 mmol, time 18 h, yield 67%.

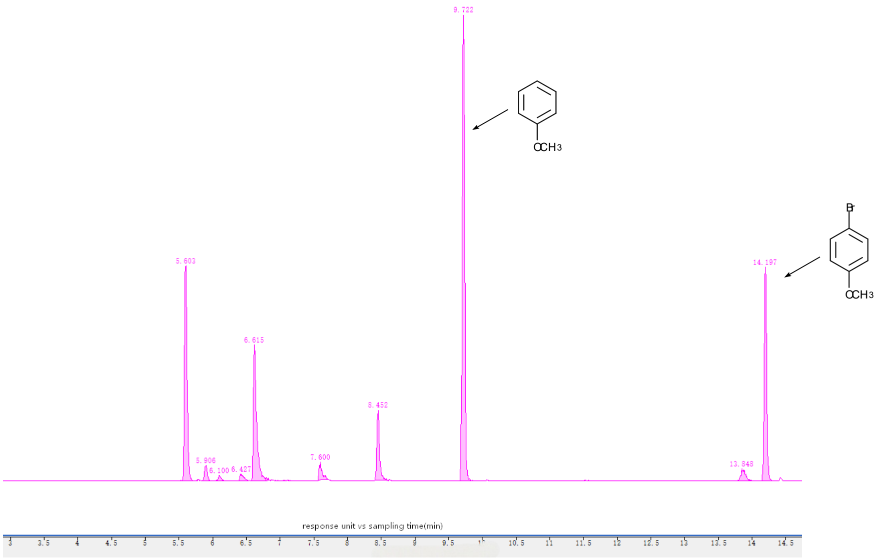

Figure S5-35 TPAdFlPh 8%, DIPEA 0.076 mmol, Cs_2CO_3 0.038 mmol, time 18 h, yield 69%.

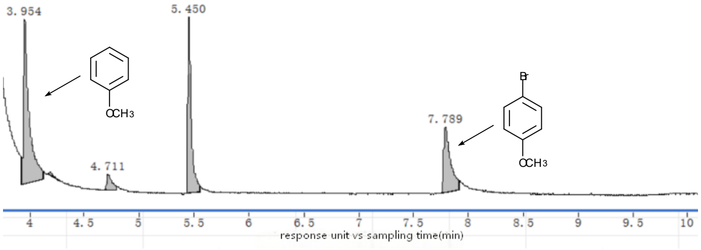

Figure S5-36 TPAdFlMe 8%, DIPEA 0.076 mmol, Cs_2CO_3 0.038 mmol, time 24 h, yield 71%.

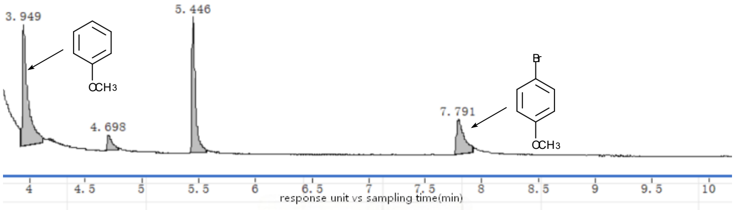

Figure S5-37 TPAdFlMe 8%, DIPEA 0.076 mmol, Cs_2CO_3 0.038 mmol, time 24 h, yield 87%.

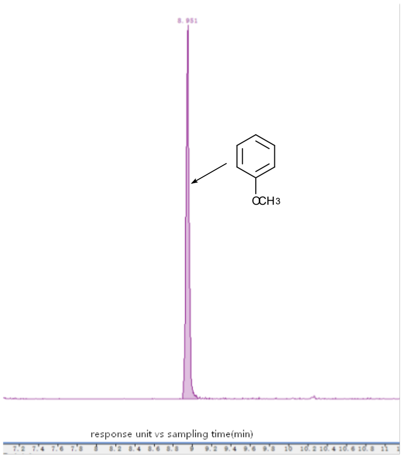

Figure S5-38 TPAdFlMe 8%, DIPEA 0.076 mmol, Cs_2CO_3 0.038 mmol, time 18 h, yield 100%.

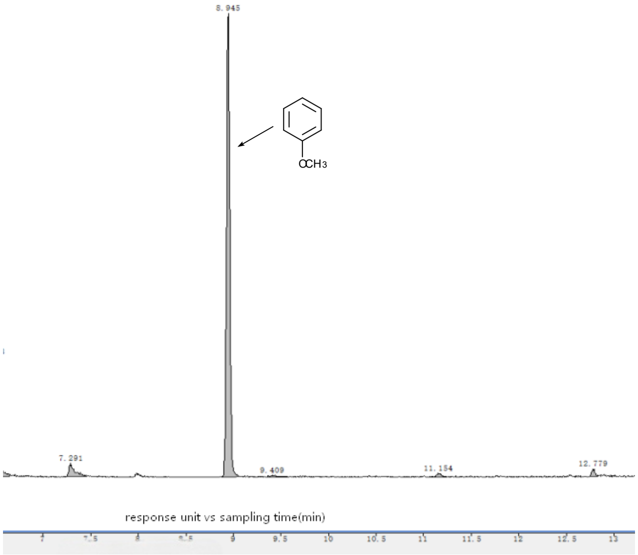

Figure S5-39 TPAdFlPh 8%, DIPEA 0.076 mmol, Cs_2CO_3 0.038 mmol, time 18 h, yield 100%.

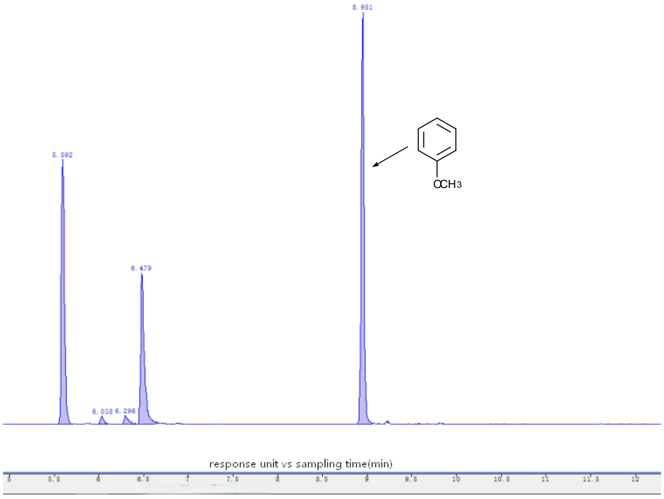

Figure S5-40 TPAdFlMe 8%, DIPEA 0.076 mmol, Cs_2CO_3 0.038 mmol, time 24 h, yield 100%.

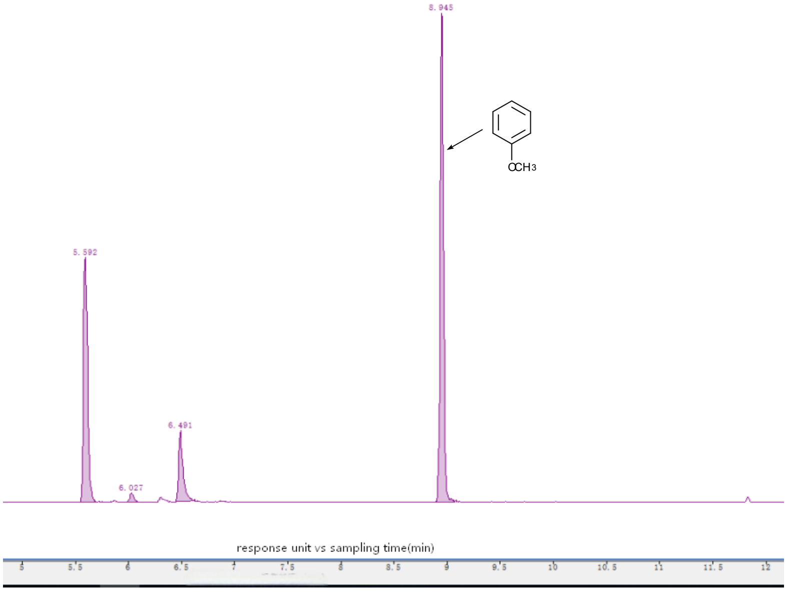

Figure S5-41 TPAdFlPh 8%, DIPEA 0.076 mmol, Cs_2CO_3 0.038 mmol, time 24 h, yield 100%.

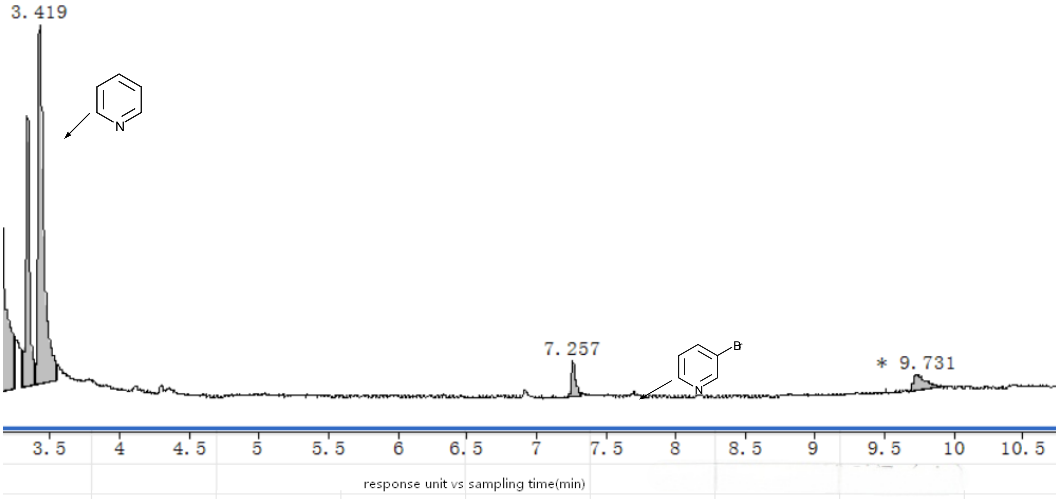

Figure S5-42 TPAdFlMe 8%, DIPEA 0.076 mmol, Cs_2CO_3 0.038 mmol, time 18 h, yield 92%.

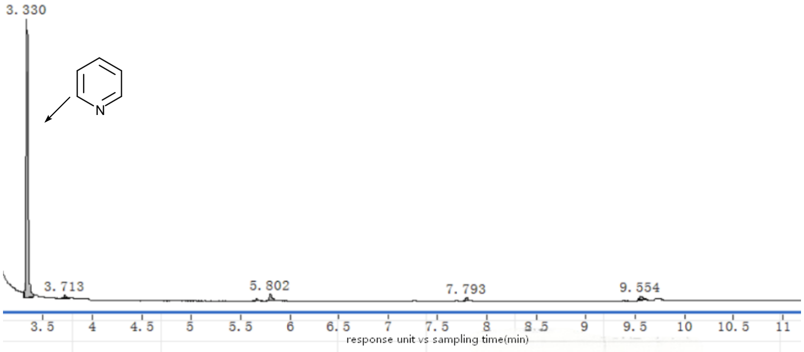

Figure S5-43 TPAdFlPh 8%, DIPEA 0.076 mmol, Cs_2CO_3 0.038 mmol, time 18 h, yield 97%.

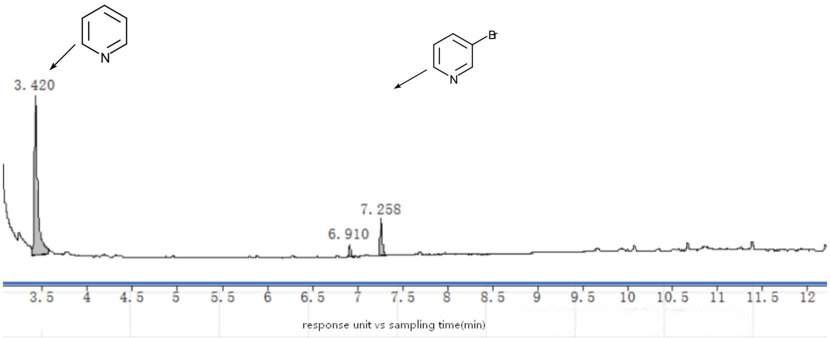

Figure S5-44 TPAdFlMe 8%, DIPEA 0.076 mmol, Cs_2CO_3 0.038 mmol, time 24 h, yield 96%.

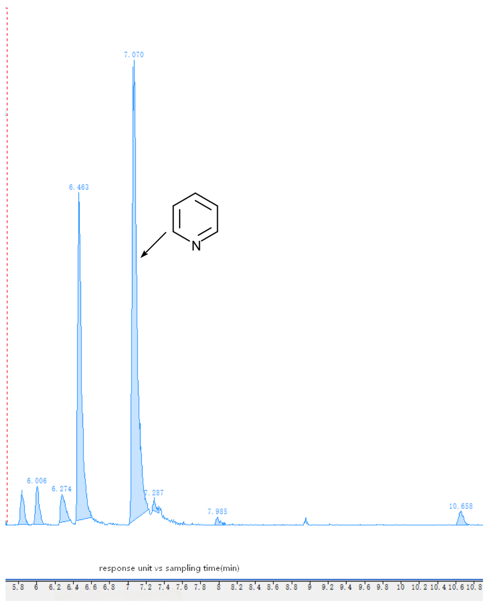

Figure S5-45 TPAdFlPh 8%, DIPEA 0.076 mmol, Cs_2CO_3 0.038 mmol, time 24 h, yield 100%.

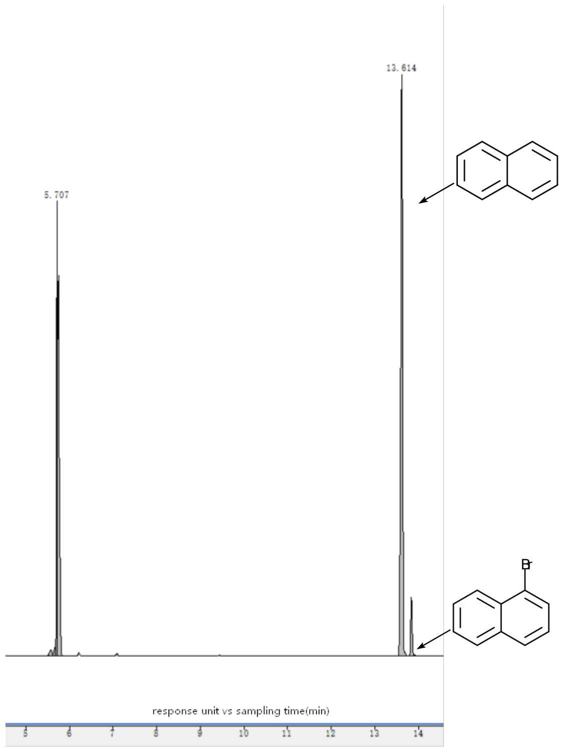

Figure S5-46 TPAdFlMe 8%, DIPEA 0.076 mmol, Cs_2CO_3 0.038 mmol, time 18 h, yield 96%.

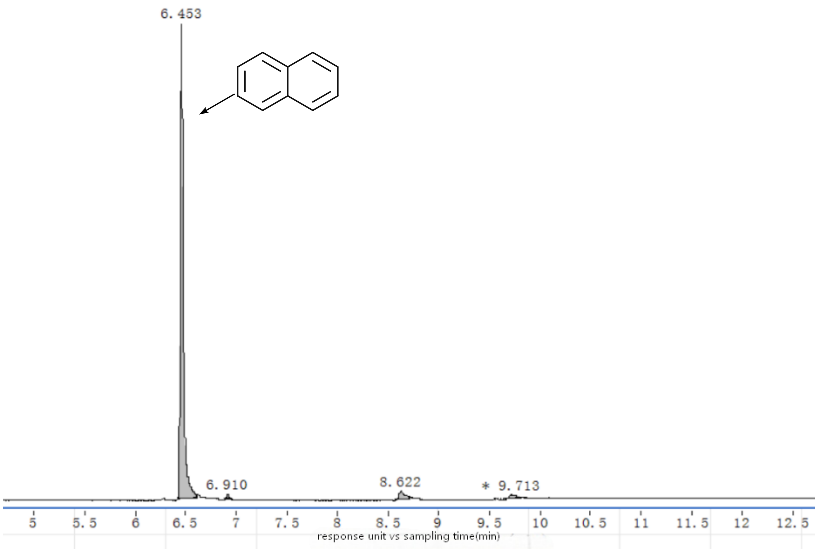

Figure S5-47 TPAdFlMe 8%, DIPEA 0.076 mmol, Cs_2CO_3 0.038 mmol, time 24 h, yield 95%.

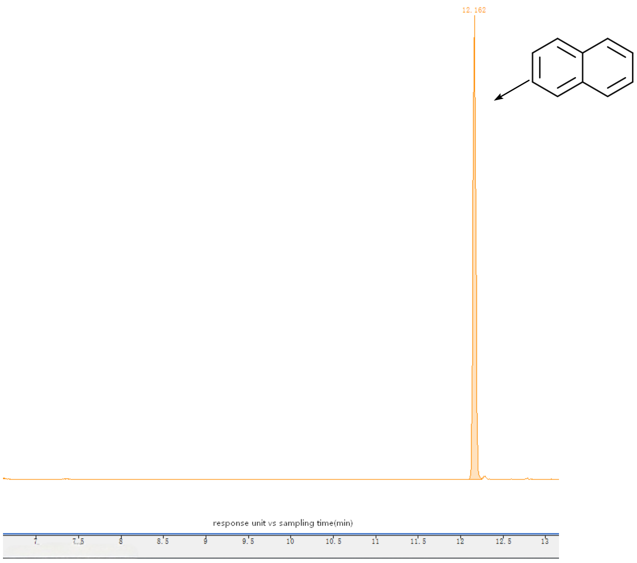

Figure S5-48 TPAdFlPh 8%, DIPEA 0.076 mmol, Cs_2CO_3 0.038 mmol, time 18 h, yield 100%.

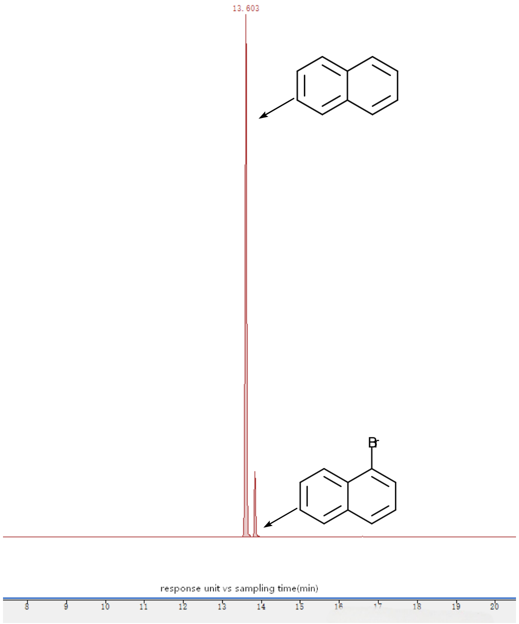

Figure S5-49 TPAdFlPh 8%, DIPEA 0.076 mmol, Cs_2CO_3 0.038 mmol, time 24 h, yield 94%.

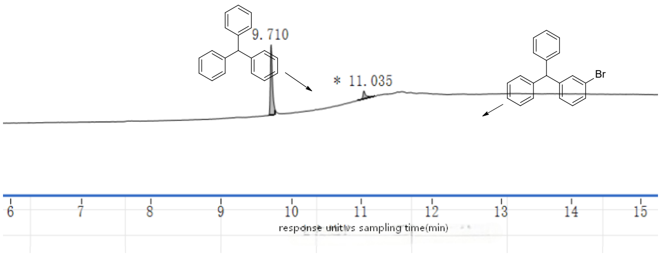

Figure S5-50 TPAdFlMe 8%, DIPEA 0.076 mmol, Cs_2CO_3 0.038 mmol, time 18 h, yield 86%.

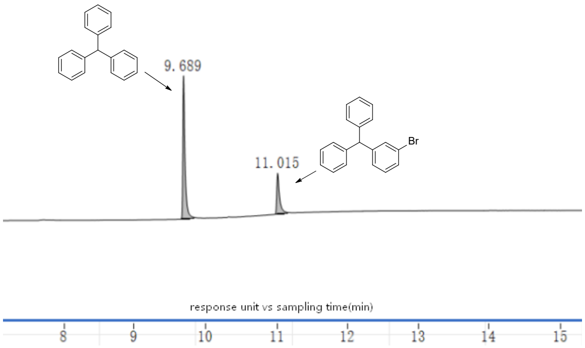

Figure S5-51 dFl$_{ph}$ 8%, DIPEA 0.076 mmol, Cs_2CO_3 0.038 mmol, time 18 h, yield 73%.

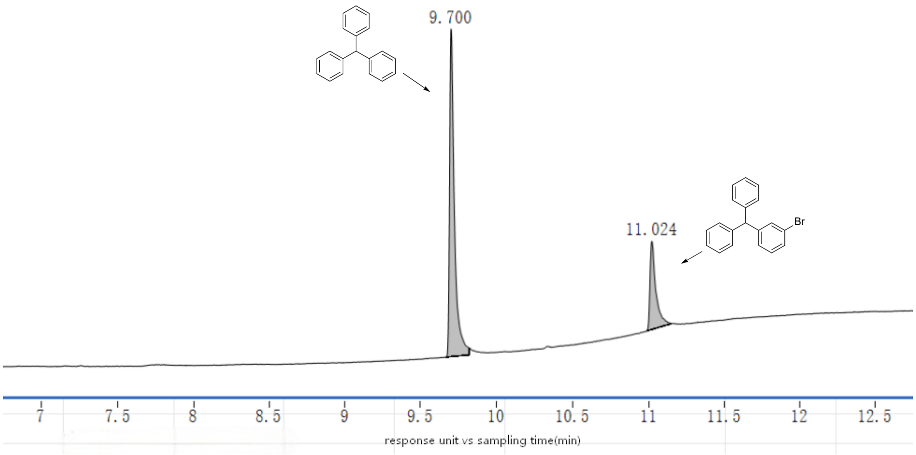

Figure S5-52 TPAdFlMe 8%, DIPEA 0.076 mmol, Cs_2CO_3 0.038 mmol, time 24 h, yield 75%.

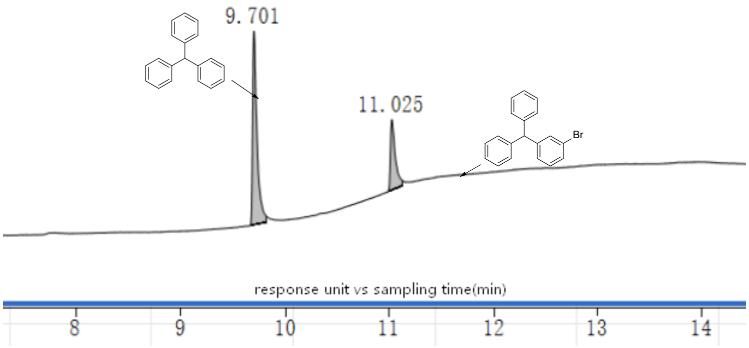

Figure S5-53 TPAdFlPh 8%, DIPEA 0.076 mmol, Cs_2CO_3 0.038 mmol, time 24 h, yield 71%.

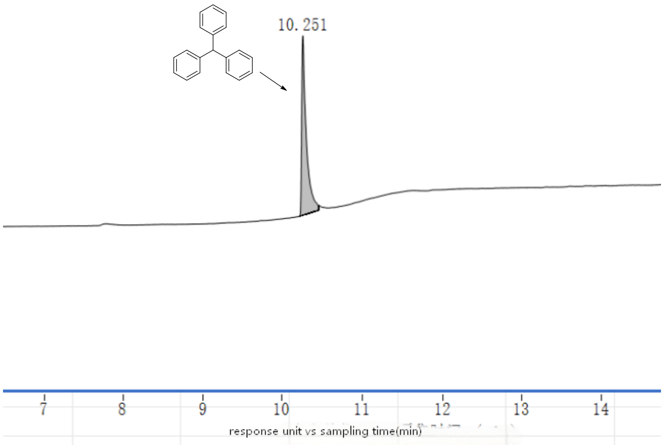

Figure S5-54 TPAdFlMe 8%, DIPEA 0.076 mmol, Cs_2CO_3 0.038 mmol, time18 h, yield 96%.

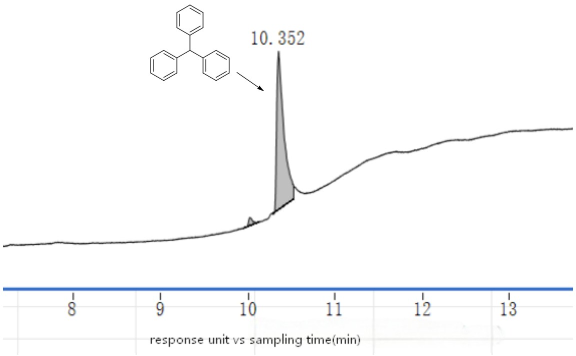

Figure S5-55 TPAdFlPh 8%, DIPEA 0.076 mmol, Cs_2CO_3 0.038 mmol, time 18 h, yield 97%.

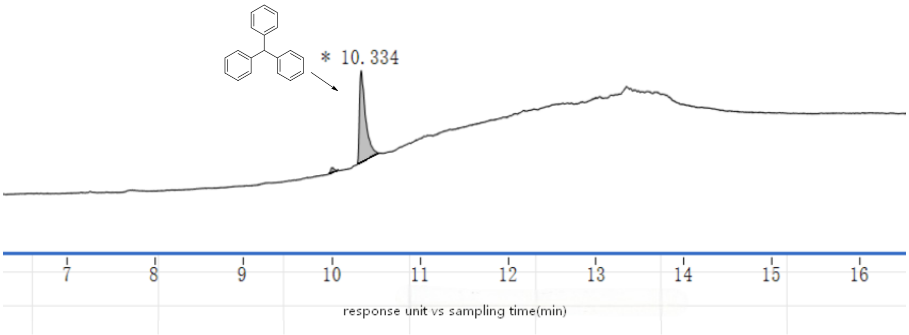

Figure S5-56 TPAdFlMe 8%, DIPEA 0.076 mmol, Cs_2CO_3 0.038 mmol, time 24 h, yield 97%.

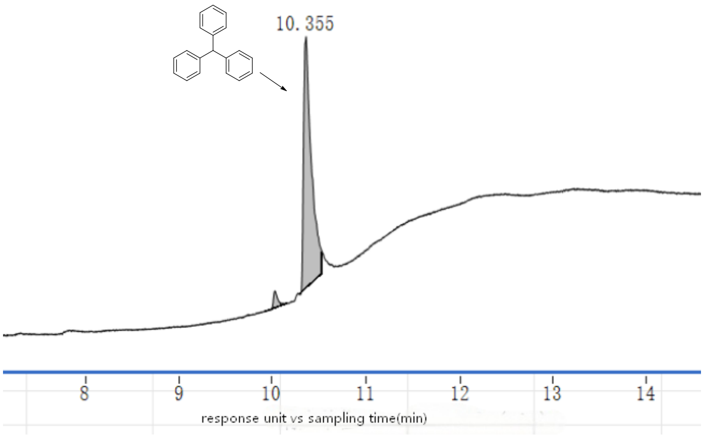

Figure S5-57 TPAdFlPh 8%, DIPEA 0.076 mmol, Cs_2CO_3 0.038 mmol, time 24 h, yield 97%.

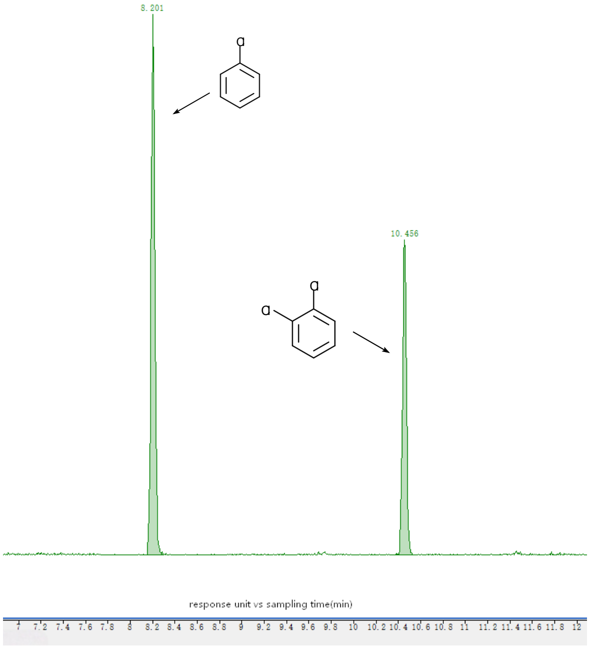

Figure S5-58 TPAdFlMe 8%, DIPEA 0.076 mmol, Cs_2CO_3 0.038 mmol, time 18 h, yield 62%.

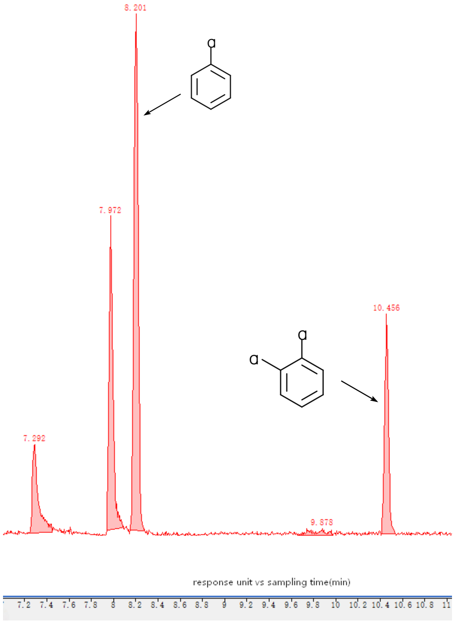

Figure S5-59 TPAdFlPh 8%, DIPEA 0.076 mmol, Cs_2CO_3 0.038 mmol, time 18 h, yield 73%.

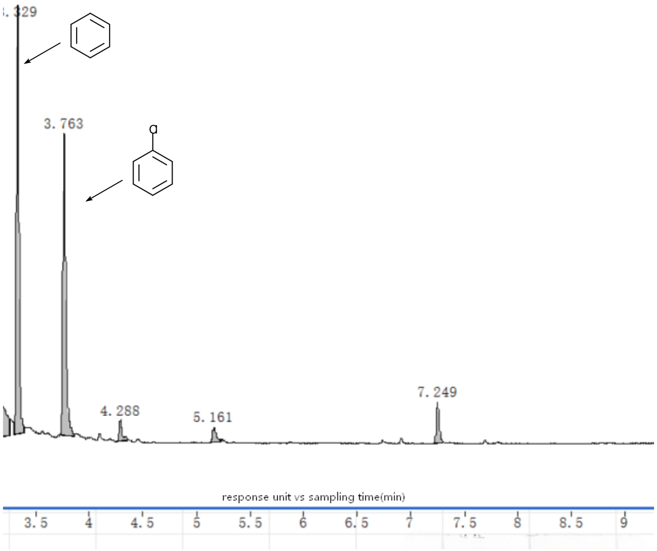

Figure S5-60 TPAdFlMe 8%, DIPEA 0.076 mmol, Cs_2CO_3 0.038 mmol, time 24 h, yield 56 %Benzene +37%Chlorobenzene.

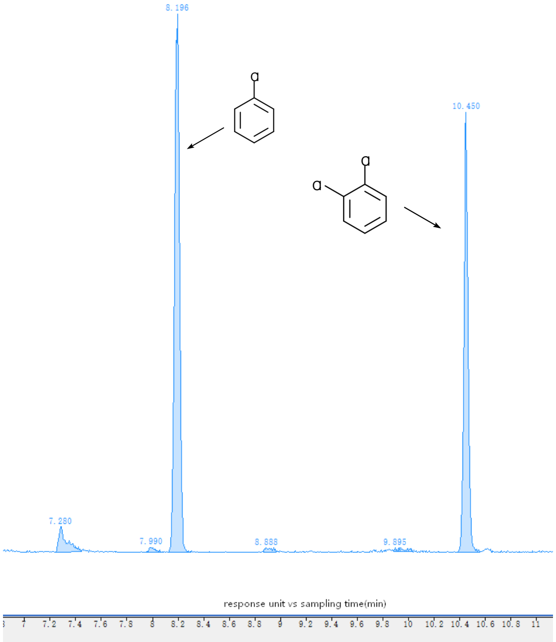

Figure S5-61 TPAdFlPh 8%, DIPEA 0.076 mmol, Cs_2CO_3 0.038 mmol, time 24 h, yield 58%.

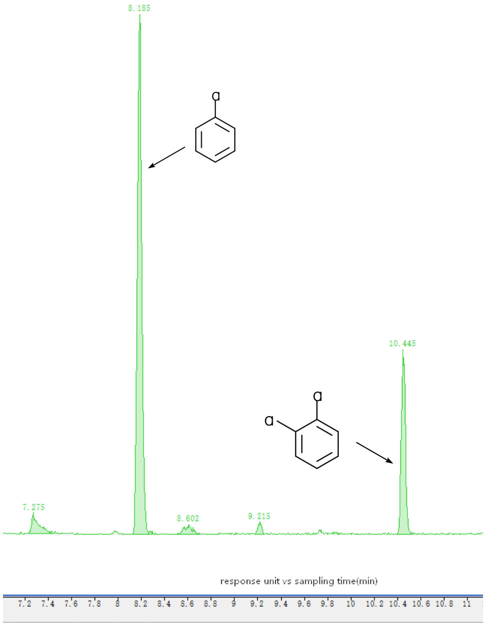

Figure S5-62 TPAdFlMe 16%, DIPEA 0.152 mmol, Cs_2CO_3 0.076 mmol, time 18 h, yield 76%.

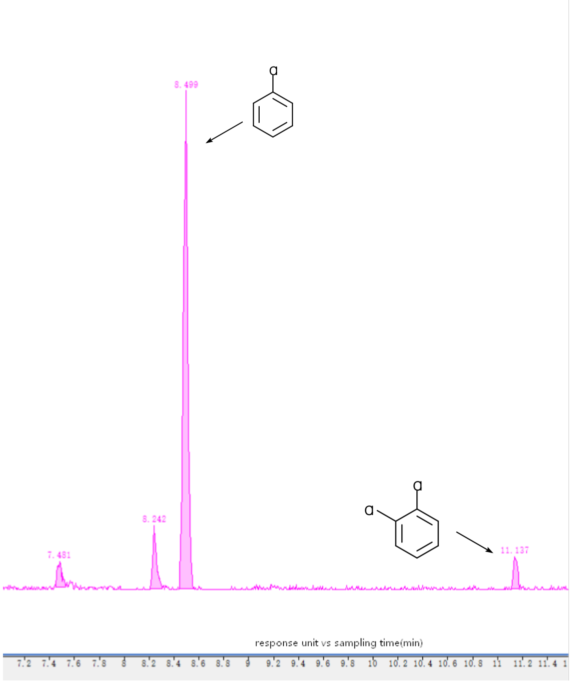

Figure S5-63 TPAdFlPh 16%, DIPEA 0.152 mmol, Cs_2CO_3 0.076 mmol, time 18 h, yield 95%.

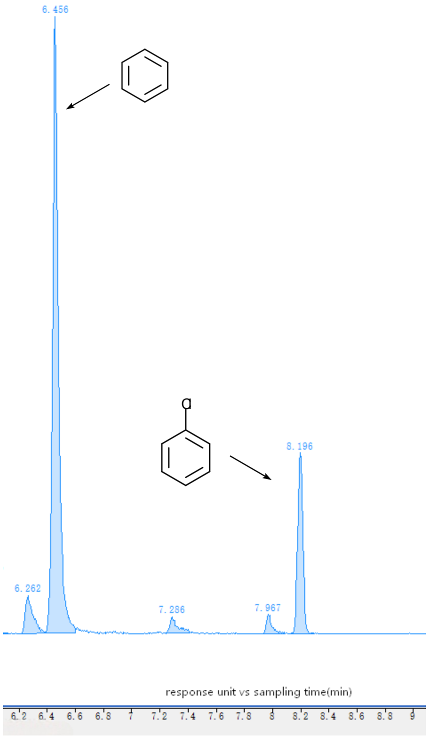

Figure S5-64 TPAdFlMe 16%, DIPEA 0.152 mmol, Cs_2CO_3 0.076 mmol, time 24 h, yield 62% Benzene+36% Chlorobenzene.

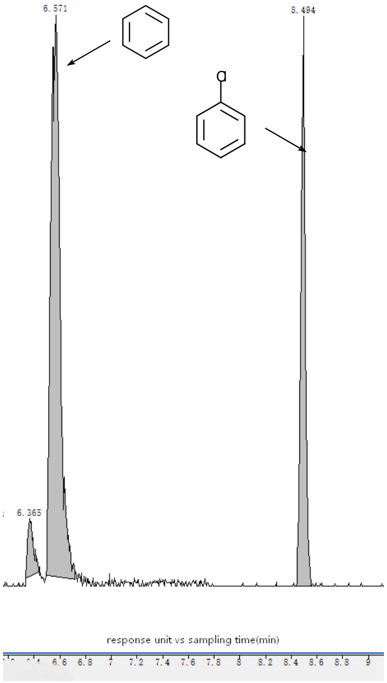

Figure S5-65 TPAdFlPh 16%, DIPEA 0.152 mmol, Cs_2CO_3 0.076 mmol, time 24 h, yield 94% Benzene+6% Chlorobenzene.

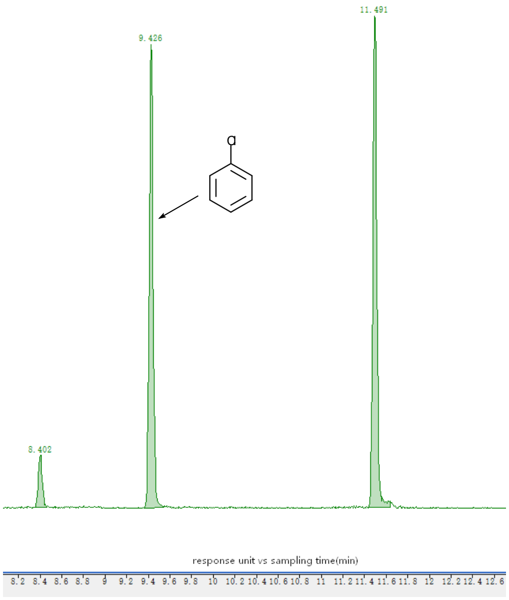

Figure S5-66 TPAdFlMe 8%, DIPEA 0.076 mmol, Cs_2CO_3 0.038 mmol, time 18 h, yield 50%.

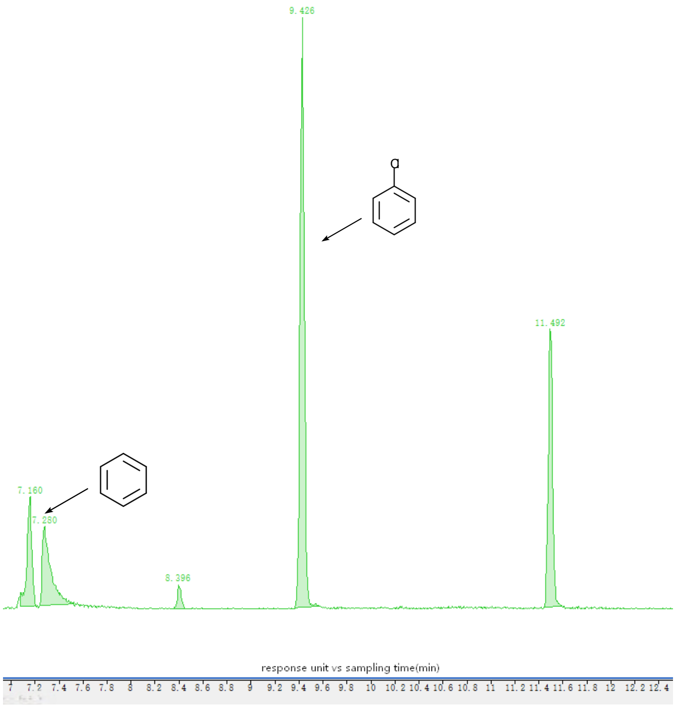

Figure S5-67 TPAdFlPh 8%, DIPEA 0.076 mmol, Cs_2CO_3 0.038 mmol, time 18 h, yield 59% Chlorobenzene +13% Benzene.

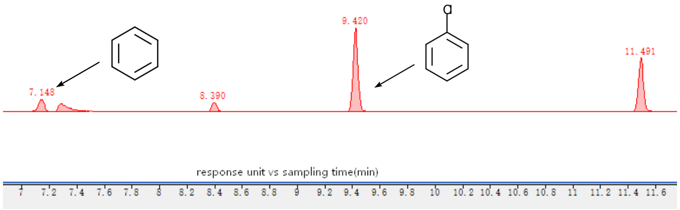

Figure S5-68 TPAdFlMe 8%, DIPEA 0.076 mmol, Cs_2CO_3 0.038 mmol, time 24 h, yield 60% Chlorobenzene +9% Benzene.

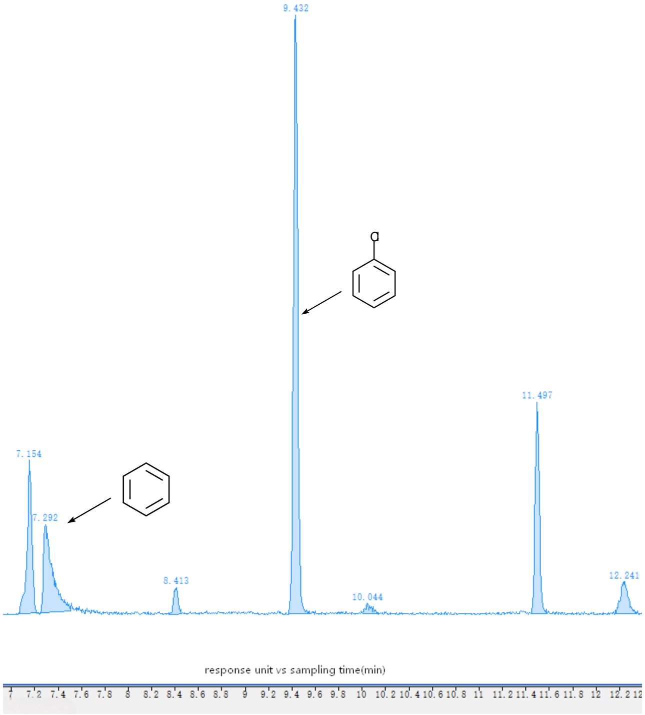

Figure S5-69 TPAdFlPh 8%, DIPEA 0.076 mmol, Cs_2CO_3 0.038 mmol, time 24 h, yield 61% Chlorobenzene +17% Benzene.

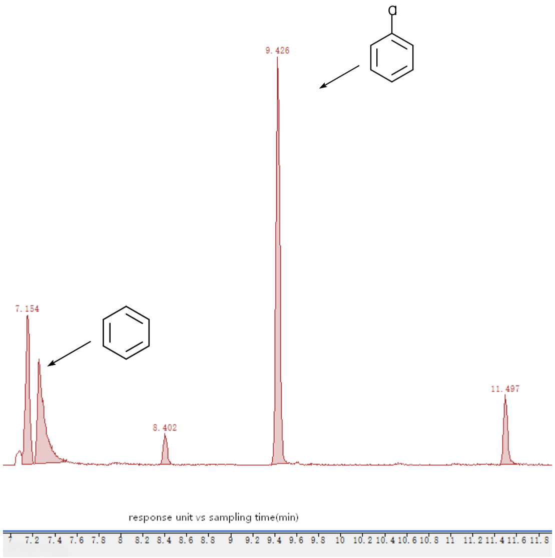

Figure S5-70 TPAdFlMe 16%, DIPEA 0.152 mmol, Cs_2CO_3 0.076 mmol, time 18 h, yield 64% Chlorobenzene +25% Benzene.

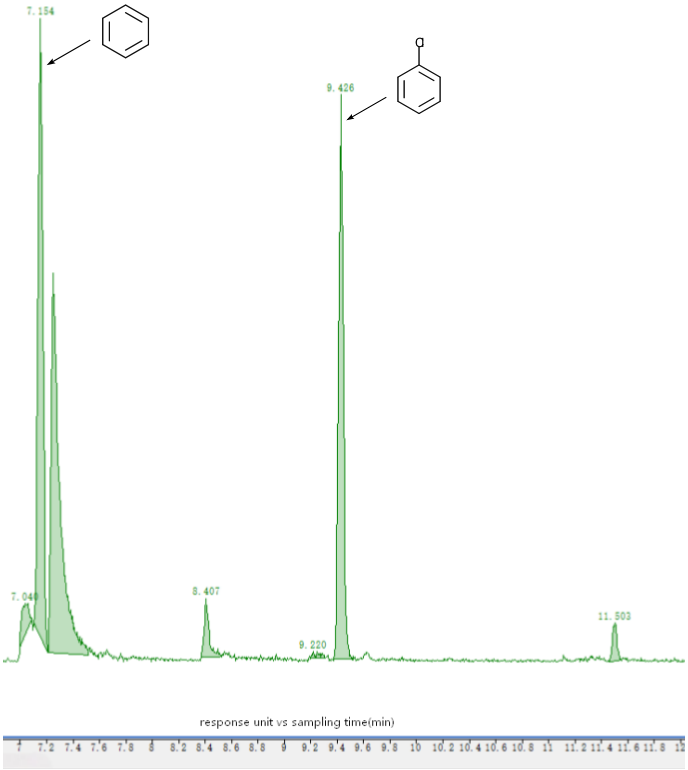

Figure S5-71 TPAdFlPh 16%, DIPEA 0.152 mmol, Cs_2CO_3 0.076 mmol, time 18 h, yield 46% Chlorobenzene +51% Benzene.

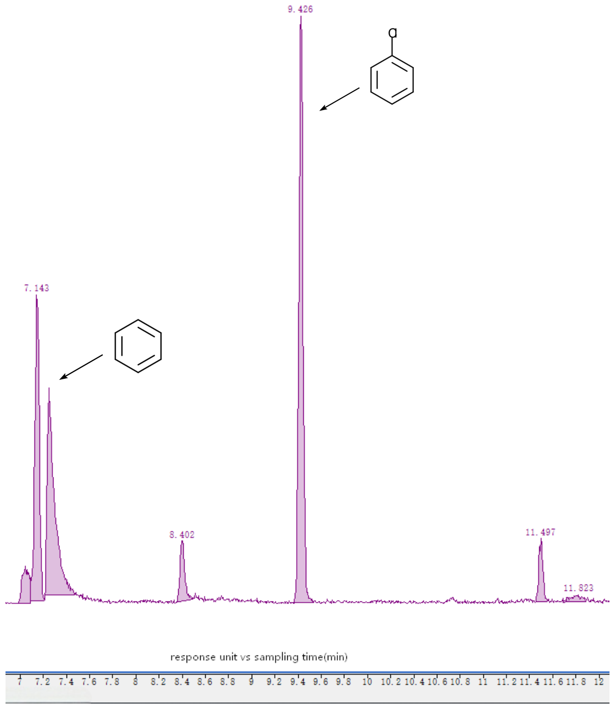

Figure S5-72 TPAdFlMe 16%, DIPEA 0.152 mmol, Cs_2CO_3 0.076 mmol, time 24 h, yield 60% Chlorobenzene +34% Benzene.

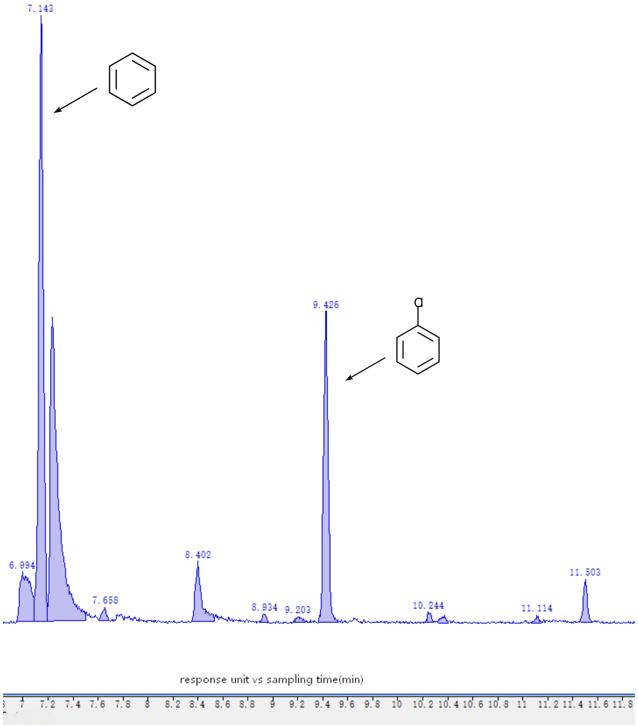

Figure S5-73 TPAdFlPh 16%, DIPEA 0.152 mmol, Cs_2CO_3 0.076 mmol, time 24 h, yield 33% Chlorobenzene +63% Benzene.

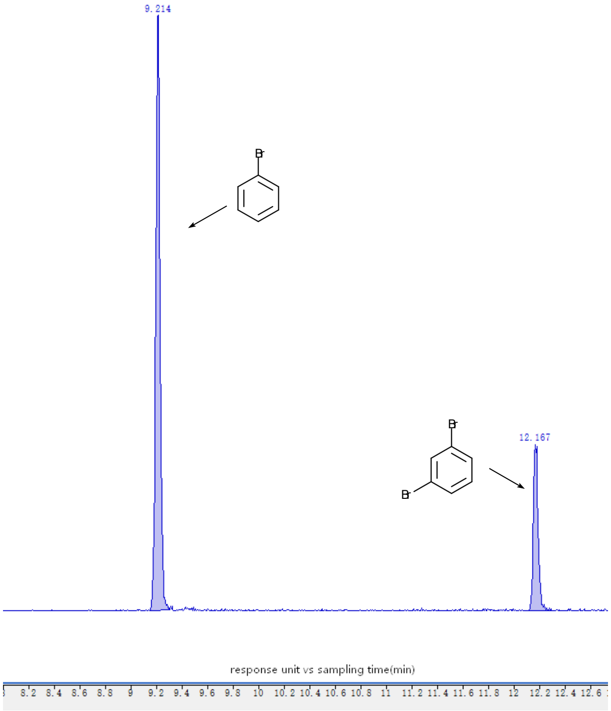

Figure S5-74 TPAdFlMe 8%, DIPEA 0.076 mmol, Cs_2CO_3 0.038 mmol, time 18 h, yield 96%.

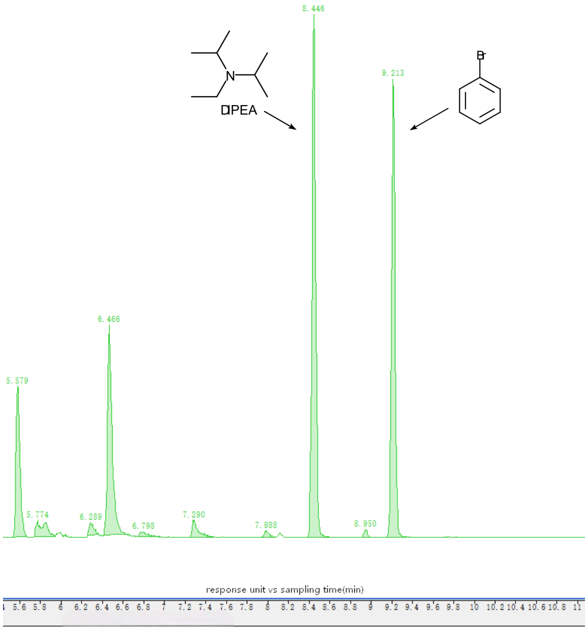

Figure S5-75 TPAdFlPh 8%, DIPEA 0.076 mmol, Cs_2CO_3 0.038 mmol, time 18 h, yield 100%.

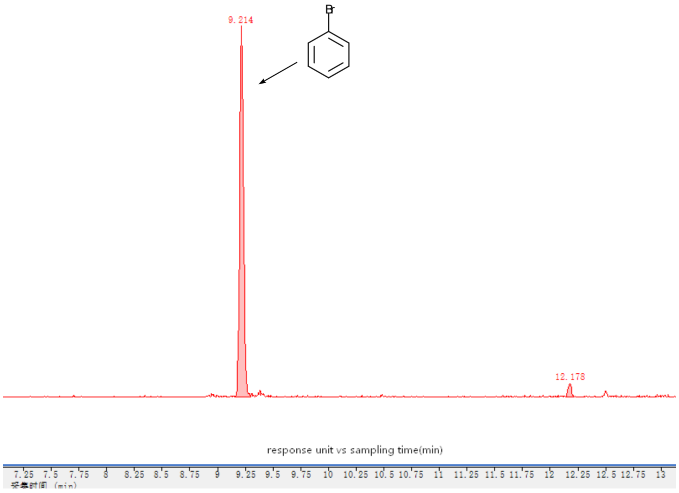

Figure S5-76 TPAdFlMe 8%, DIPEA 0.076 mmol, Cs_2CO_3 0.038 mmol, time 24 h, yield 97%.

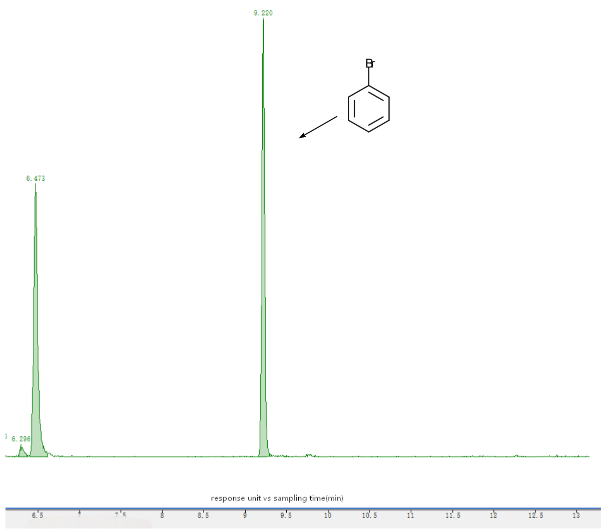

Figure S5-77 TPAdFlPh 8%, DIPEA 0.076 mmol, Cs_2CO_3 0.038 mmol, time 18 h, yield 100%.

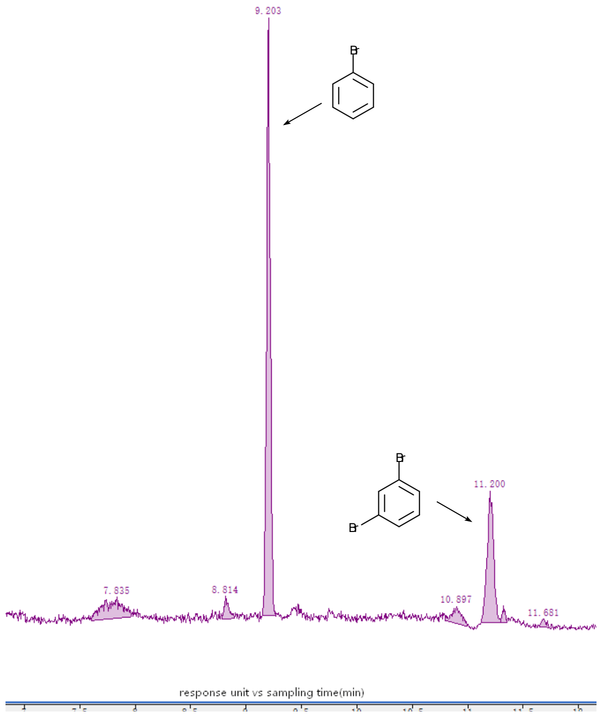

Figure S5-78 TPAdFlMe 16%, DIPEA 0.152 mmol, Cs_2CO_3 0.076 mmol, time 18 h, yield 95%.

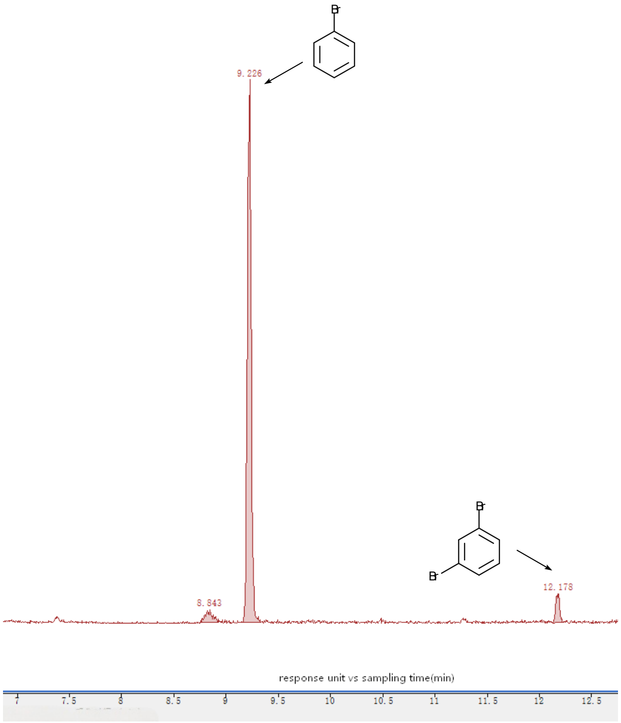

Figure S5-79 TPAdFlPh 16%, DIPEA 0.152 mmol, Cs_2CO_3 0.076 mmol, time 18 h, yield 95%.

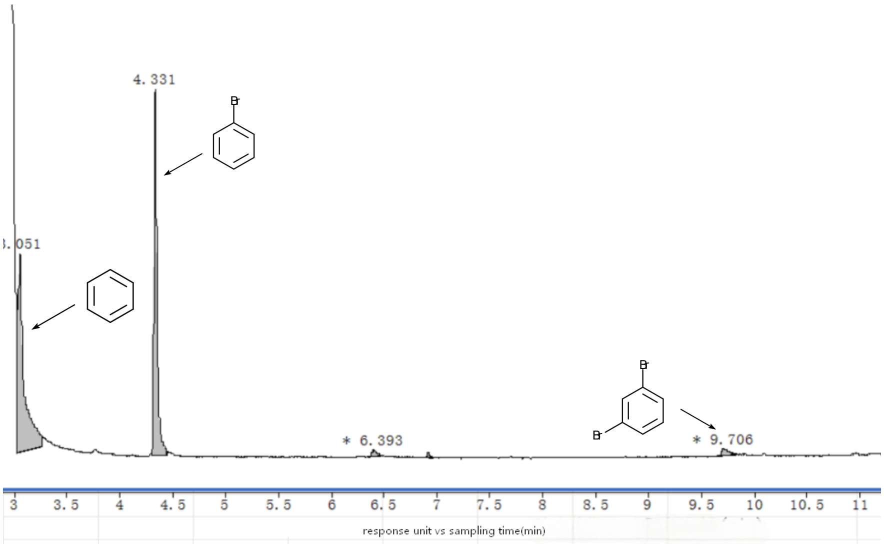

Figure S5-80 TPAdFlMe 16%, DIPEA 0.152 mmol, Cs_2CO_3 0.076 mmol, time 24 h, yield 53% Benzene +47% Bromobenzene.

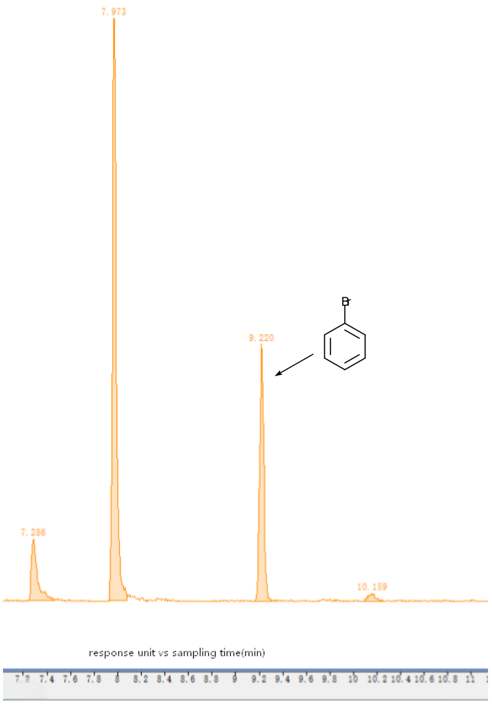

Figure S5-81 TPAdFlPh 16%, DIPEA 0.152 mmol, Cs_2CO_3 0.076 mmol, time 24 h, yield 100%.

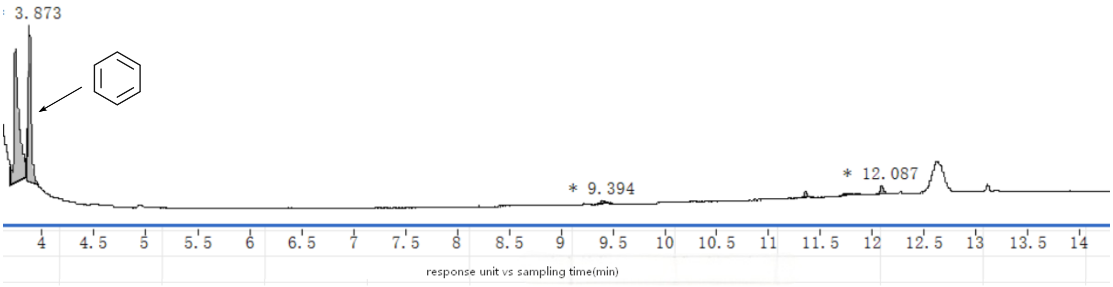

Figure S5-82 TPAdFlMe 8%, DIPEA 0.076 mmol, Cs_2CO_3 0.076 mmol, time 18 h, yield 94%.

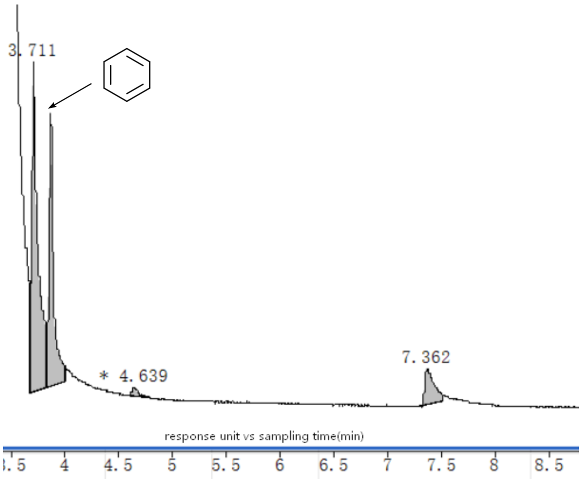

Figure S5-83 TPAdFlPh 8%, DIPEA 0.076 mmol, Cs_2CO_3 0.076 mmol, time 18 h, yield 100%.

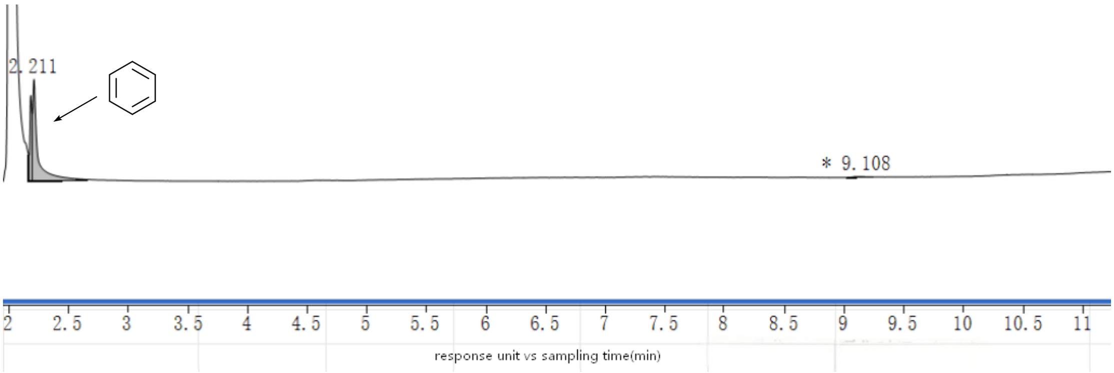

Figure S5-84 TPAdFlMe 8%, DIPEA 0.076 mmol, Cs_2CO_3 0.076 mmol, time 24 h, yield 97%.

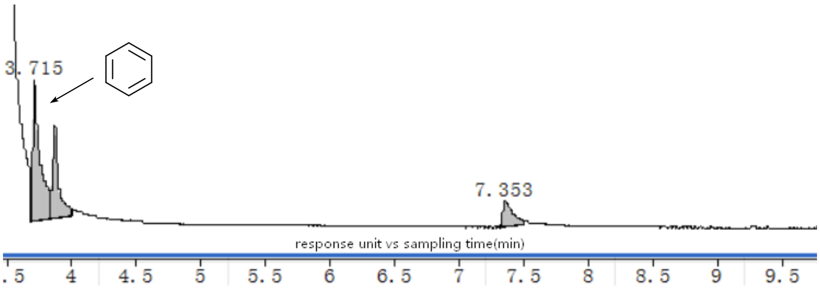

Figure S5-85 TPAdFlPh 8%, DIPEA 0.076 mmol, Cs_2CO_3 0.076 mmol, time 24 h, yield 100%.

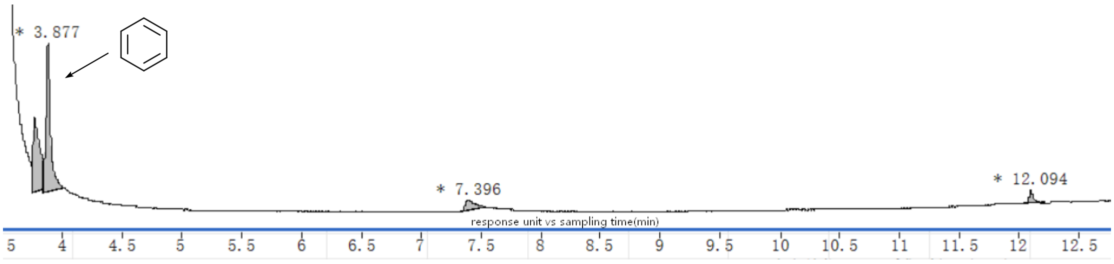

Figure S5-86 TPAdFlMe 16%, DIPEA 0.152 mmol, Cs_2CO_3 0.076 mmol, time 18 h, yield 96%.

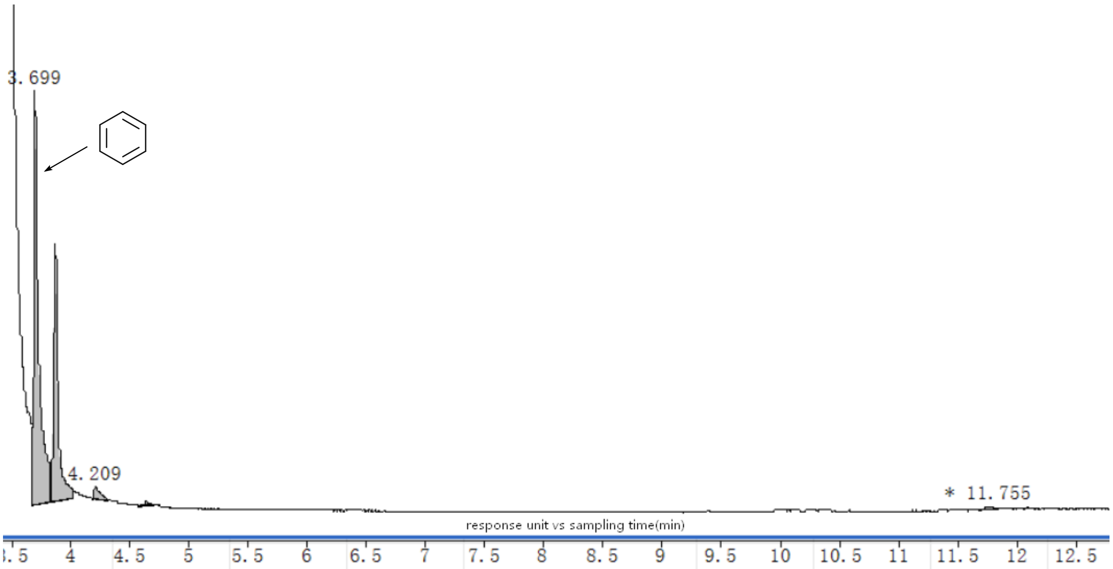

Figure S5-87 TPAdFlPh 16%, DIPEA 0.152 mmol, Cs_2CO_3 0.076 mmol, time 18 h, yield 96%.

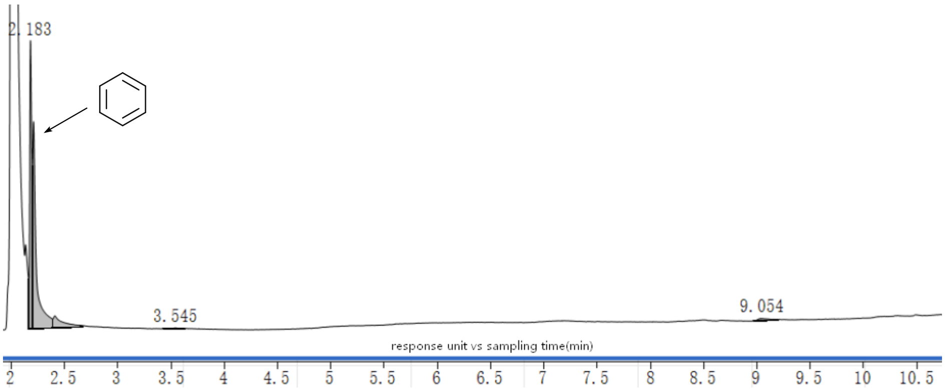

Figure S5-88 TPAdFlMe 16%, DIPEA 0.152 mmol, Cs_2CO_3 0.076 mmol, time 24 h, yield 97%.

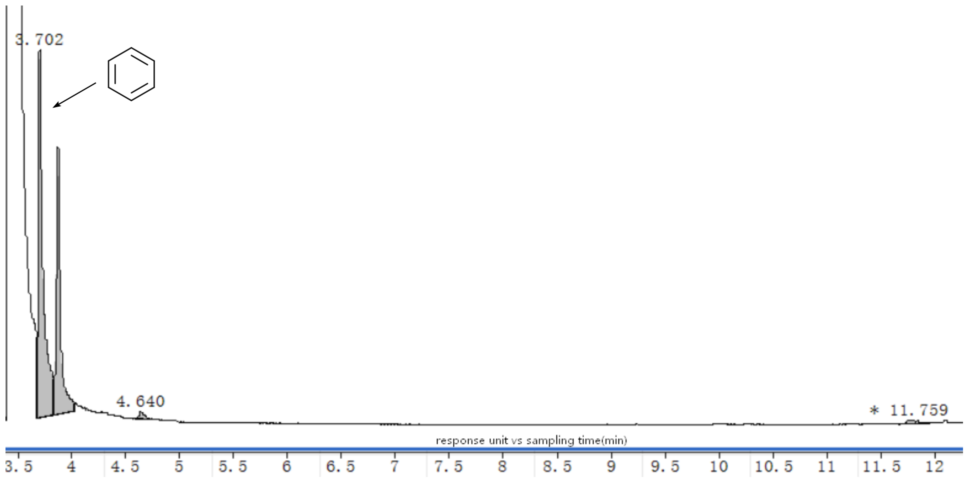

Figure S5-89 TPAdFlPh 16%, DIPEA 0.152 mmol, Cs_2CO_3 0.076 mmol, time 24 h, yield 97%.

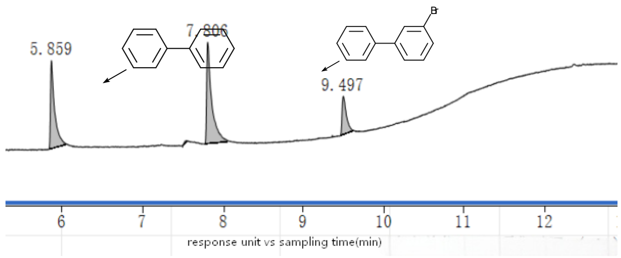

Figure S5-90 TPAdFlMe 8%, DIPEA 0.076 mmol, Cs_2CO_3 0.038 mmol, time 18 h,

yield 29% 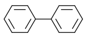 +50% 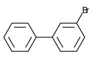 .

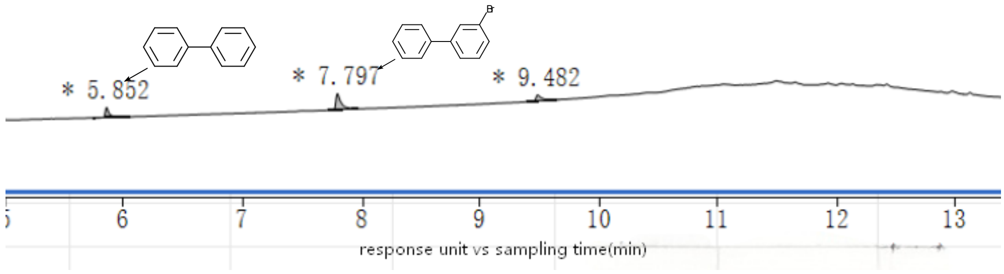

Figure S5-91 TPAdFlPh 8%, DIPEA 0.076 mmol, Cs_2CO_3 0.038 mmol, time 18 h,

yield 25%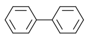+52%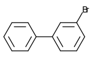 .

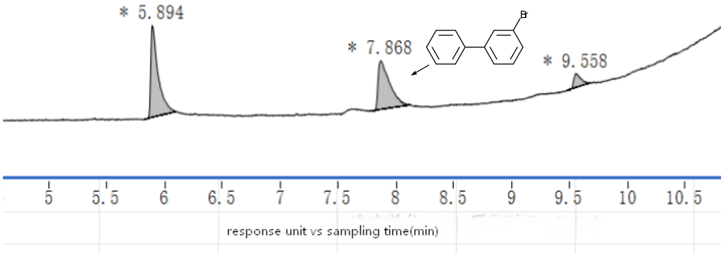

Figure S5-92 TPAdFlMe 8%, DIPEA 0.076 mmol, Cs_2CO_3 0.038 mmol, time 24 h, yield 51%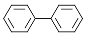+42%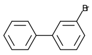.

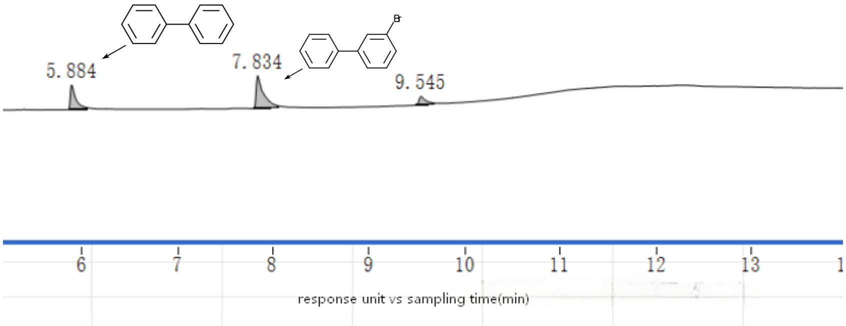

Figure S5-93 TPAdFlPh 8%, DIPEA 0.076 mmol, Cs_2CO_3 0.038 mmol, time 24 h, yield 32%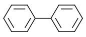+55%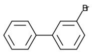.

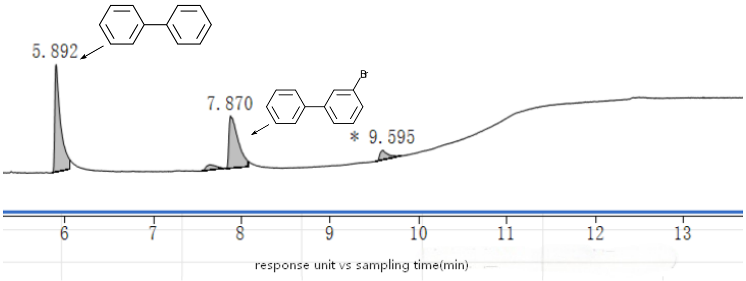

Figure S5-94 TPAdFlMe 16%, DIPEA 0.152 mmol, Cs_2CO_3 0.076 mmol, time 18 h, yield 60%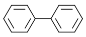+37%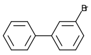.

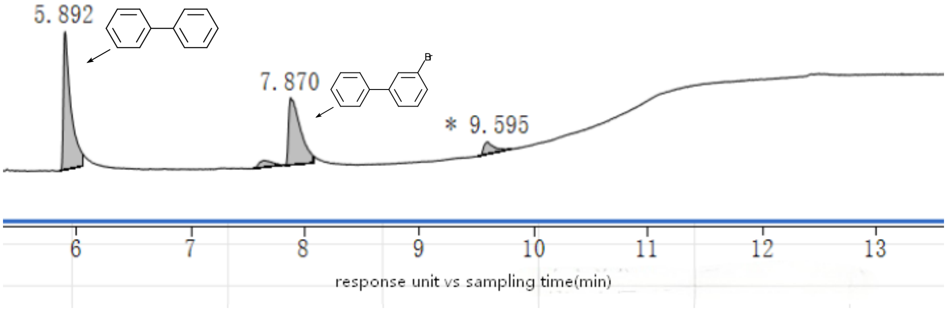

Figure S5-95 TPAdFlPh 16%, DIPEA 0.152 mmol, Cs_2CO_3 0.076 mmol, time 18 h, yield 53%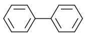+41%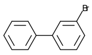.

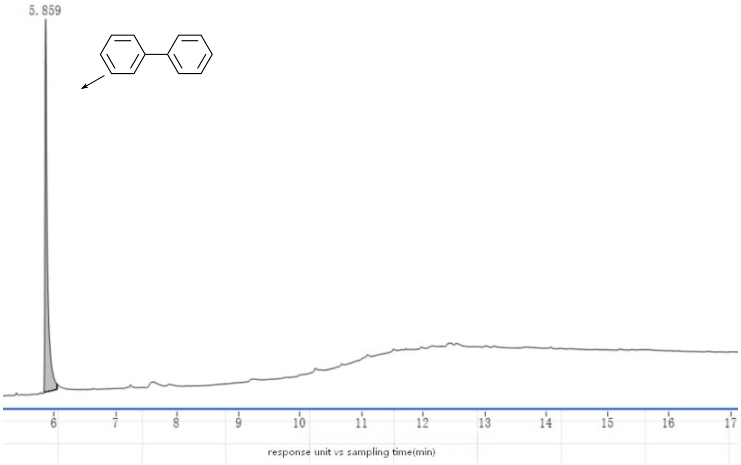

Figure S5-96 TPAdFlMe 16%, DIPEA 0.152 mmol, Cs_2CO_3 0.076 mmol, time 24 h, yield 100%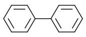.

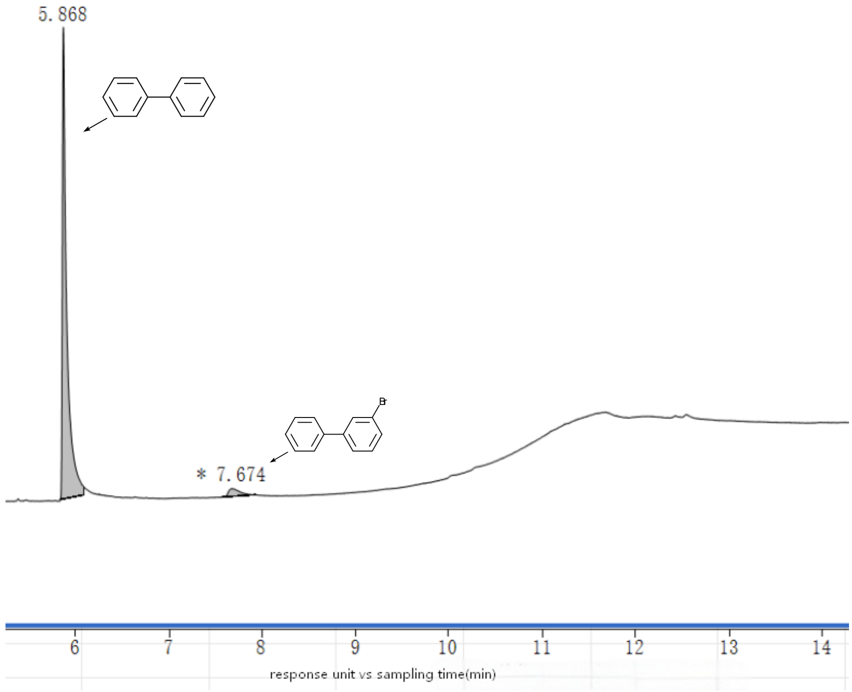

Figure S5-97 TPAdFlPh 16%, DIPEA 0.152 mmol, Cs_2CO_3 0.076 mmol, time 24 h, yield 96%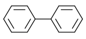+4%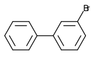.

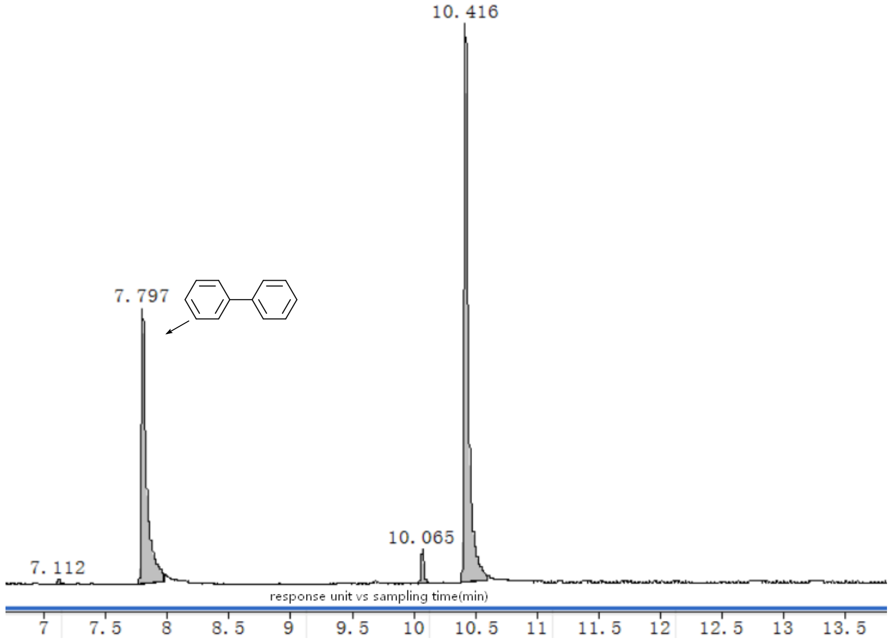

Figure S5-98 TPAdFlMe 8%, DIPEA 0.076 mmol, Cs_2CO_3 0.038 mmol, time 18 h, yield 38%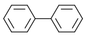+62%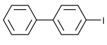.

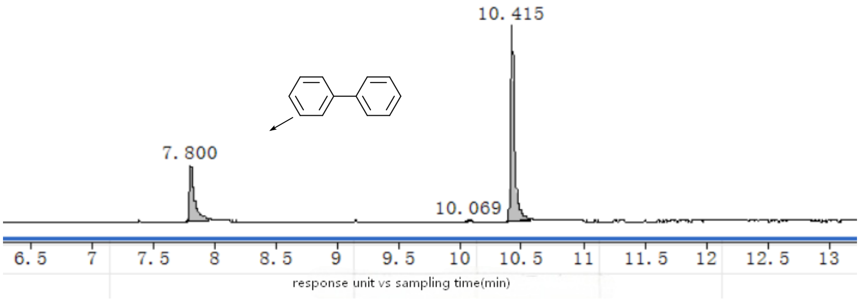

Figure S5-99 TPAdFlPh 8%, DIPEA 0.076 mmol, Cs_2CO_3 0.038 mmol, time 18 h, yield 28%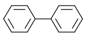+72%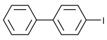.

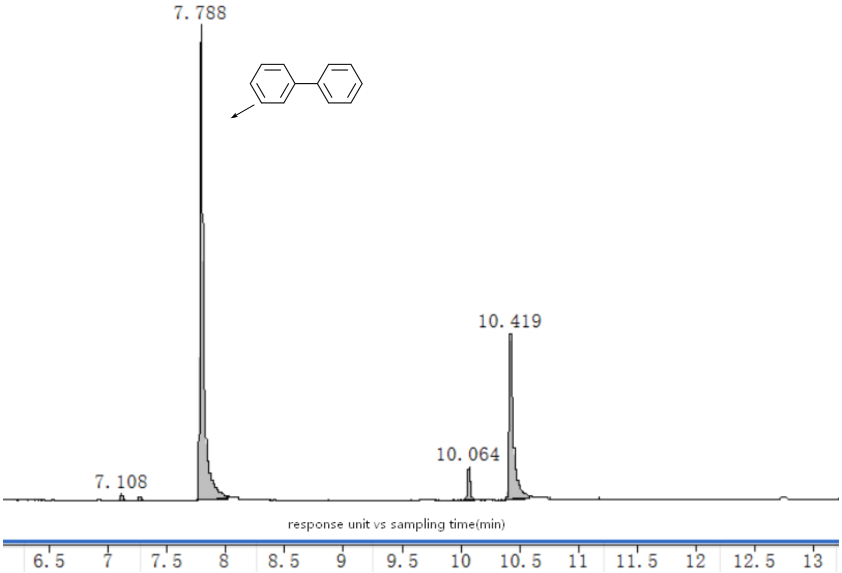

Figure S5-100 TPAdFlMe 8%, DIPEA 0.076 mmol, Cs_2CO_3 0.038 mmol, time 24 h, yield 70%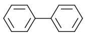+29%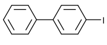.

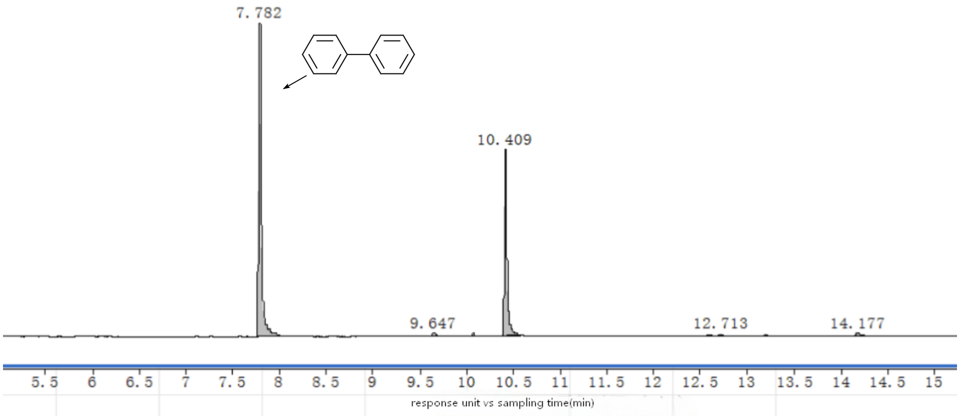

Figure S5-101 TPAdFlPh 8%, DIPEA 0.076 mmol, Cs_2CO_3 0.038 mmol, time 24 h, yield 62% 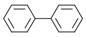 +38% 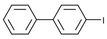.

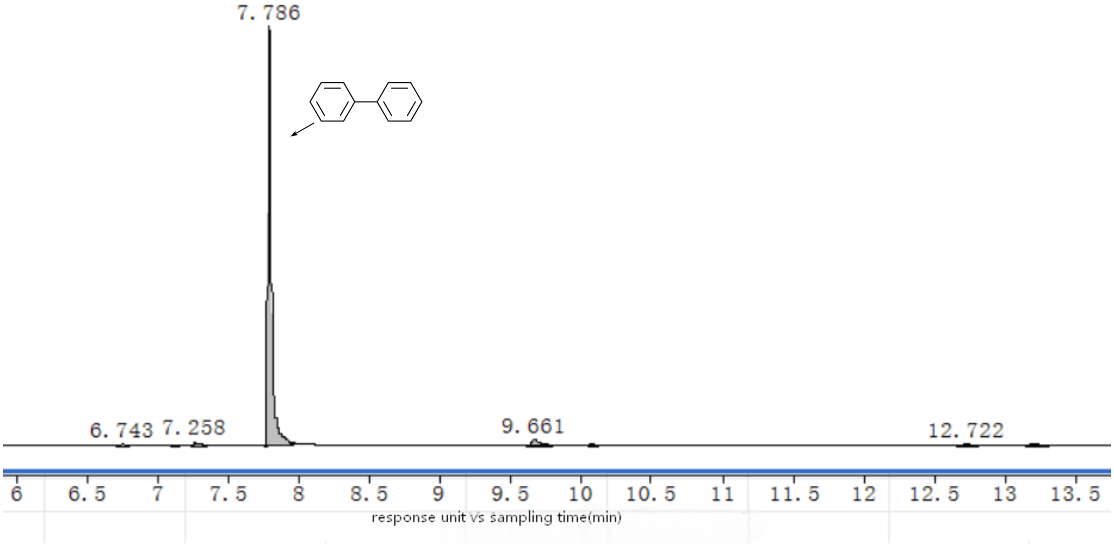

Figure S5-102 TPAdFlMe 16%, DIPEA 0.152 mmol, Cs_2CO_3 0.076 mmol, time 18 h, yield 100%.

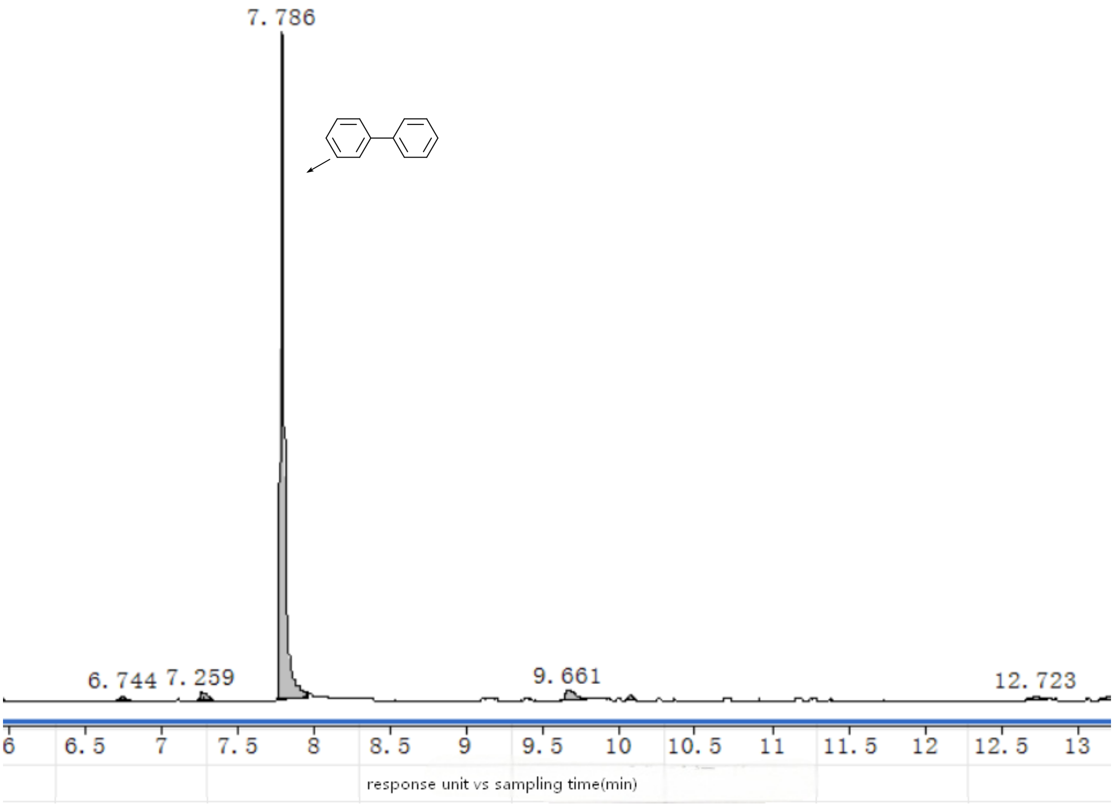

Figure S5-103 TPAdFlPh 16%, DIPEA 0.152 mmol, Cs_2CO_3 0.076 mmol, time 18 h, yield 100%.

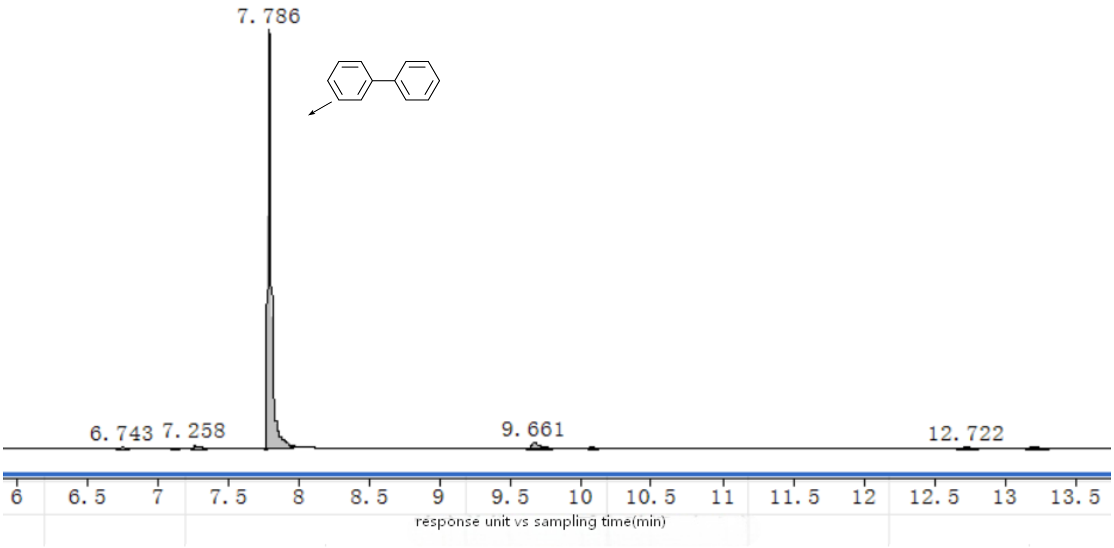

Figure S5-104 TPAdFlMe 16%, DIPEA 0.152 mmol, Cs_2CO_3 0.076 mmol, time 24 h, yield 100%.

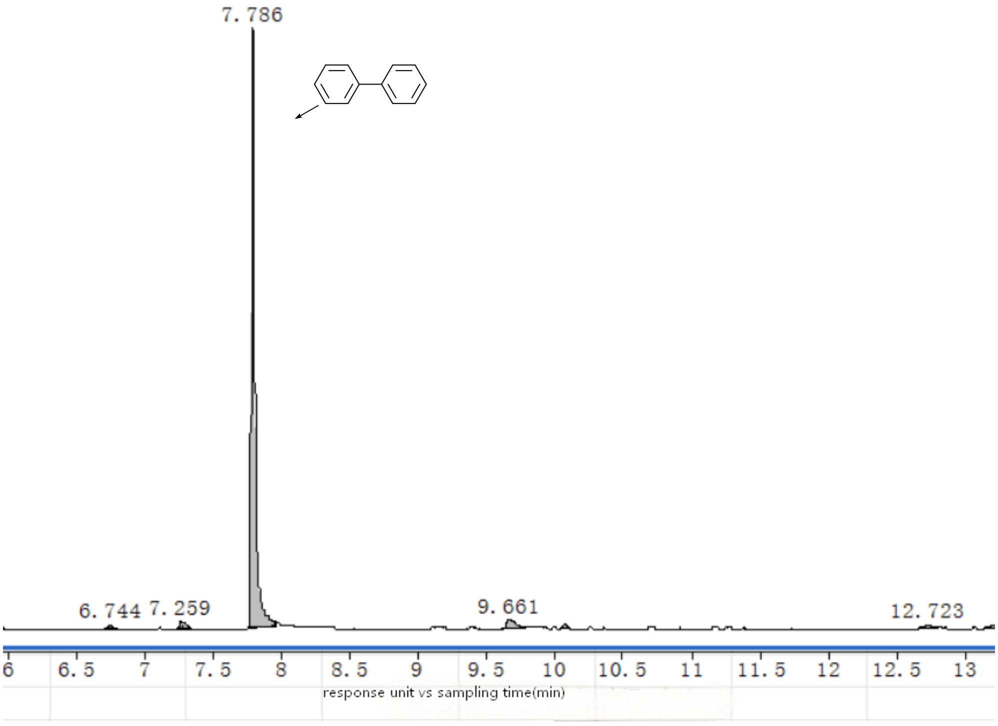

Figure S5-105 TPAdFlPh16%, DIPEA 0.152 mmol, Cs_2CO_3 0.076 mmol, time 24 h, yield 100%.

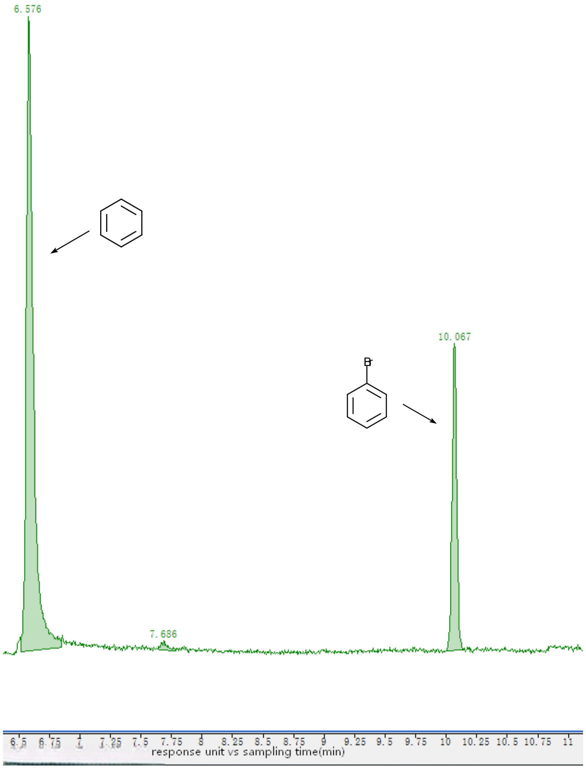

Figure S5-106 TPAdFlMe 8%, DIPEA 0.076 mmol, Cs_2CO_3 0.038 mmol, DABCQ 8%, time 18 h, yield 75%.

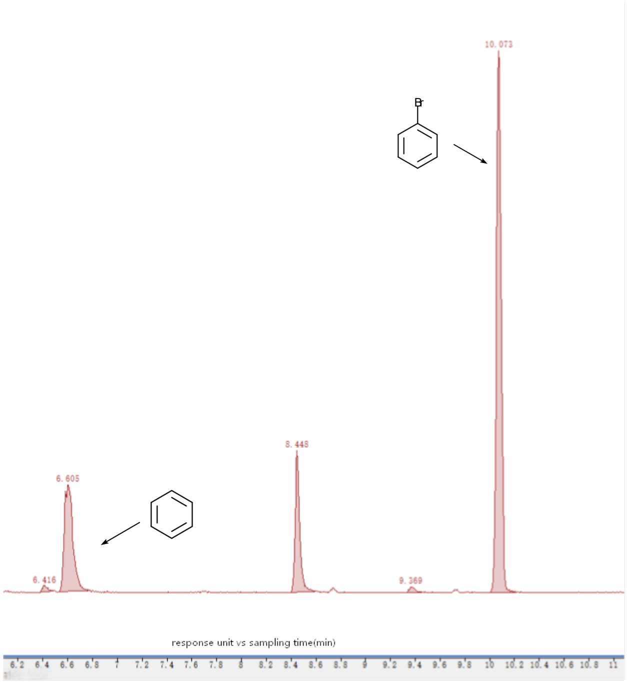

Figure S5-107 TPAdFlMe 8%, DIPEA 0.076 mmol, Cs_2CO_3 0.038 mmol, BQ 80%, time 18 h, yield 25%.

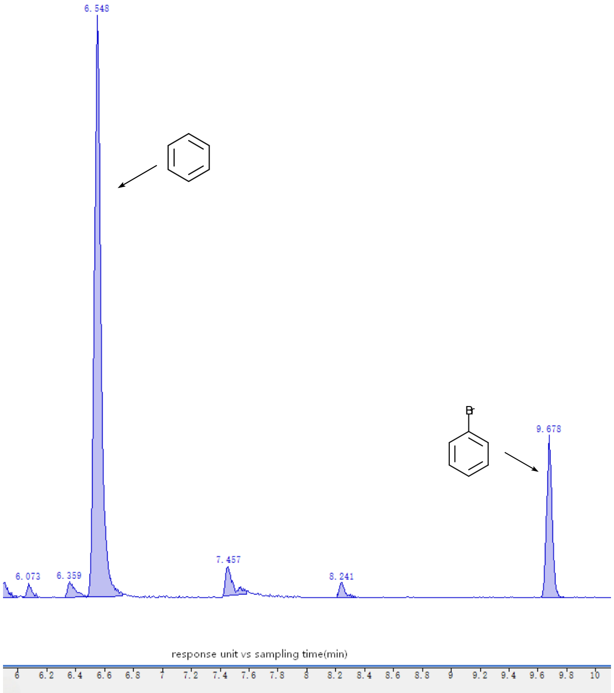

Figure S5-108 TPAdFlMe 8%, DIPEA 0.076 mmol, Cs_2CO_3 0.038 mmol, TEMPO 8%, time 18 h, yield 82%.

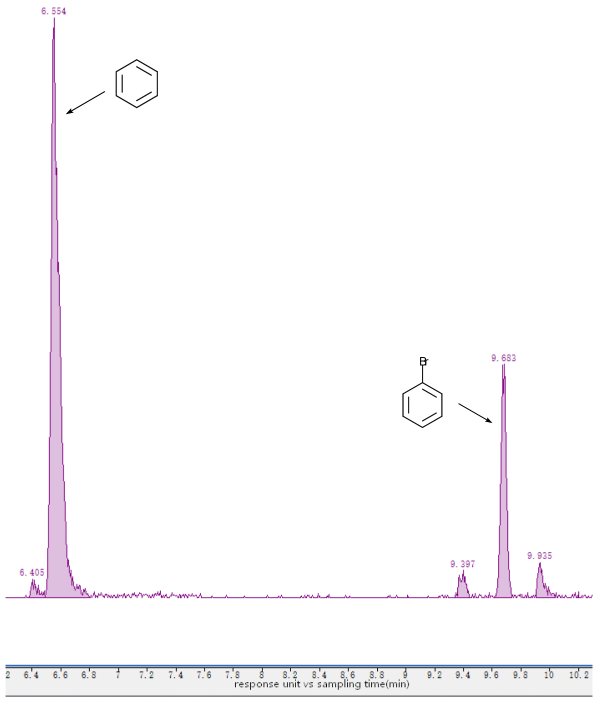

Figure S5-109 TPAdFlMe 8%, DIPEA 0.076 mmol, Cs_2CO_3 0.038 mmol, TEMPO 80%, time 18 h, yield 79%.

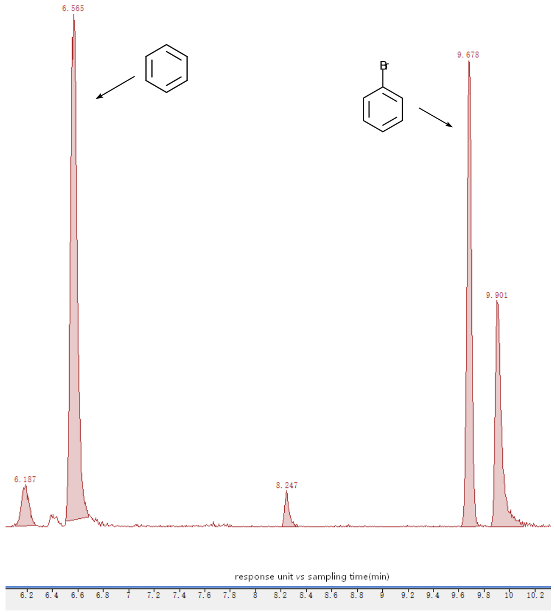

Figure S5-110 TPAdFlMe 8%, DIPEA 0.076 mmol, Cs_2CO_3 0.038 mmol, TEMPO 160%, time 18 h, yield 62%.

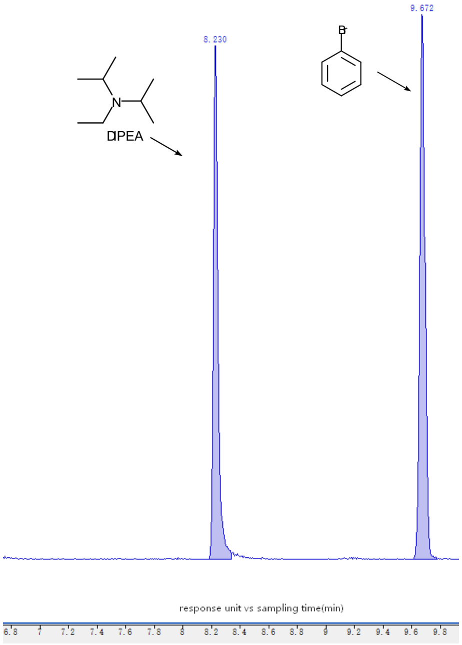

Figure S5-111 TPAdFlMe 8%, DIPEA 0.076 mmol, Cs_2CO_3 0.038 mmol, TEMPO 240%, time 18 h, yield 0%.

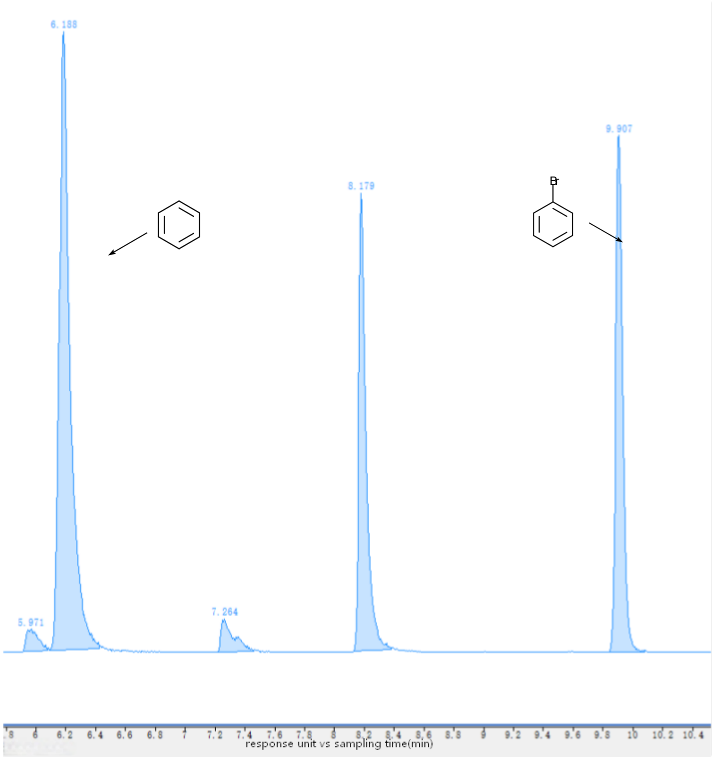

Figure S5-112 TPAdFlPh 8%, DIPEA 0.076 mmol, Cs_2CO_3 0.038 mmol, DABCQ 8%, time 18 h, yield 65%.

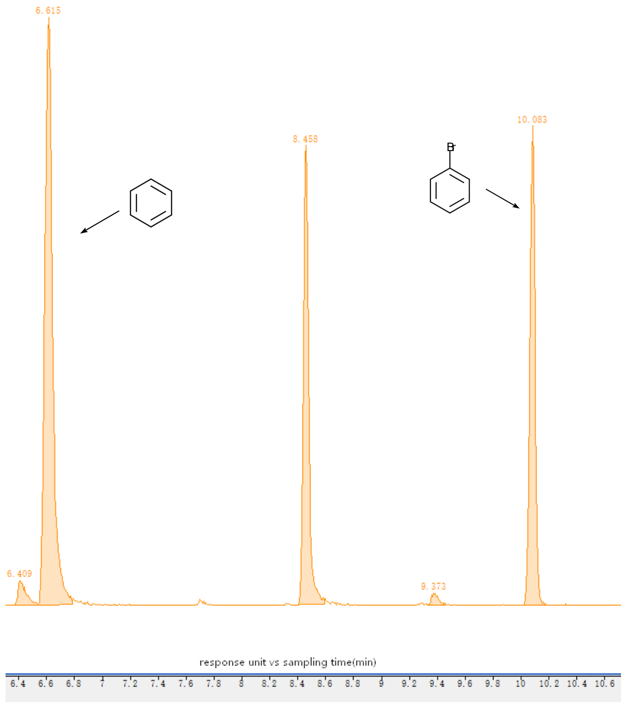

Figure S5-113 TPAdFlPh 8%, DIPEA 0.076 mmol, Cs_2CO_3 0.038 mmol, BQ 80%, time 18 h, yield 64%.

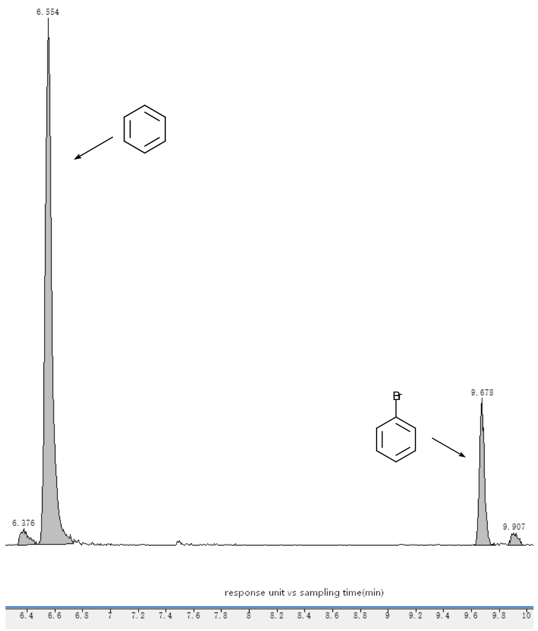

Figure S5-114 TPAdFlPh 8%, DIPEA 0.076 mmol, Cs_2CO_3 0.038 mmol, TEMPO 8%, time 18 h, yield 83%

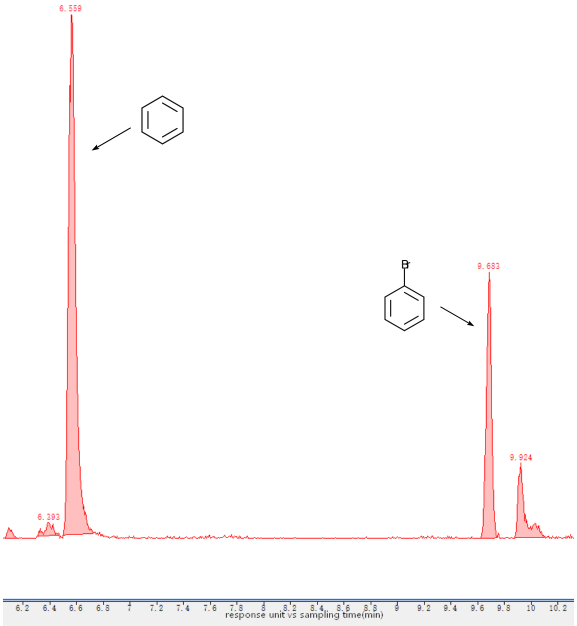

Figure S5-115 TPAdFlPh 8%, DIPEA 0.076 mmol, Cs_2CO_3 0.038 mmol, TEMPO 80%, time 18 h, yield 74%.

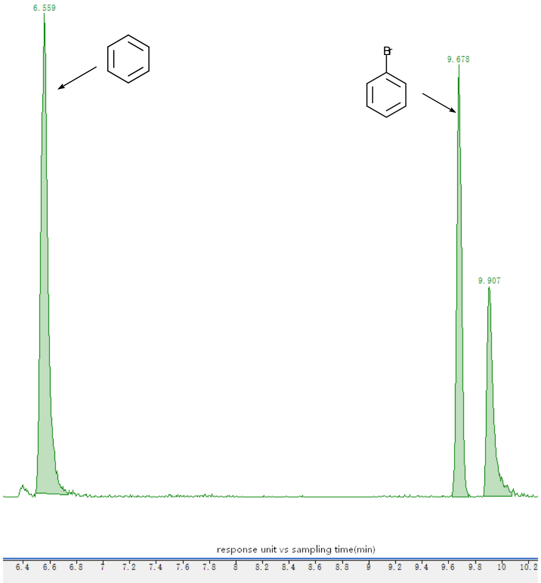

Figure S5-116 TPAdFlPh 8%, DIPEA 0.076 mmol, Cs_2CO_3 0.038 mmol, TEMPO 160%, time 18 h, yield 62%.

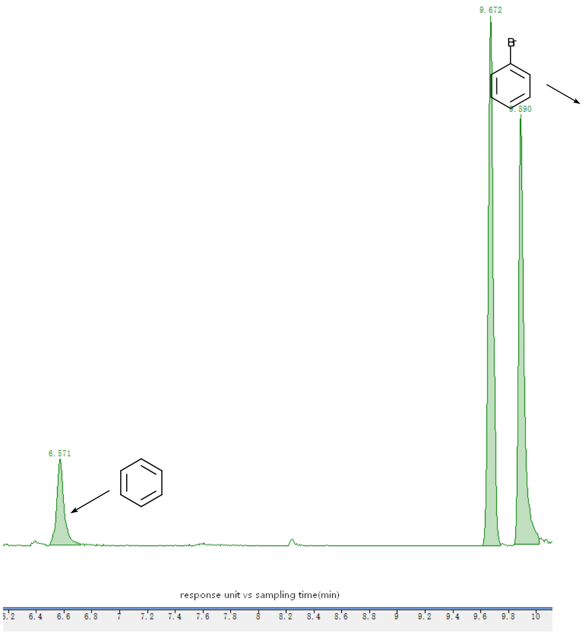

Figure S5-117 TPAdFlPh 8%, DIPEA 0.076 mmol, Cs_2CO_3 0.038 mmol, TEMPO 240%, time 18 h, yield 18%.

6. DFT/TD-DFT Results

6.1 Theoretical Methods

DFT and TD-DFT based calculations were performed to investigate the absorption properties of dFLs (Scheme 3). We used methyl was used to simplify the alkyl moieties. We fully relaxed the dFLs at B3LYP/6-311G(d) level of theory to get their ground state (S_0) structures.[1-4] In the calculations, we used Polarizable Continuum Model (PCM) to treat the acetyl nitrile (CH_3CN) solvent.[5] The absorption properties of DFLs were investigated with TD-DFT based calculations at the same level of theory. Frequency calculations were performed for all reported structures to insure their nature of minima on the potential energy surface (PES). All the DFT and TD-DFT based calculations were performed with Gaussian 16.[6]

6.2 DFT/TD-DFT Results

Table S6-1a. Electronic transitions involved in the excitation of Fl at B3LYP/6-311g(d) level of theory.

	Energy	f	Composition	Percentage [a]	Character
$S_0 \rightarrow S_1$	3.02 eV/410 nm	0.2013	59→60	96.54%	π→π*, n→π*
$S_0 \rightarrow S_2$	3.35 eV/370 nm	0.0008	56→60	54.94%	n→π*
			57→60	42.74%	n→π*
$S_0 \rightarrow S_3$	3.50 eV/354 nm	0.0000	57→60	52.69%	n→π*
			56→60	42.47%	n→π*
$S_0 \rightarrow S_4$	3.75 eV/331 nm	0.1821	58→60	92.42%	π→π*, n→π*
$S_0 \rightarrow S_5$	4.18 eV/297 nm	0.0000	54→60	92.61%	n→π*
$S_0 \rightarrow S_6$	4.24 eV/292 nm	0.0111	55→60	92.35%	π→π*, n→π*
$S_0 \rightarrow S_7$	4.69 eV/265 nm	0.0004	52→60	93.57%	n→π*
$S_0 \rightarrow S_8$	4.82 eV/257 nm	0.0158	53→60	85.50%	π→π*, n→π*
			58→61	11.10%	π→π*, n→π*
$S_0 \rightarrow S_9$	4.90 eV/259 nm	0.7373	59→61	87.04%	π→π*, n→π*
$S_0 \rightarrow S_{10}$	5.23 eV/237 nm	0.0503	56→61	62.55%	n→π*
			57→61	30.47%	n→π*
$S_0 \rightarrow T_1$	2.15 eV/576 nm	0.0000	59→60	96.79%	π→π*, n→π*
$S_0 \rightarrow T_2$	2.73 eV/454 nm	0.0000	58→60	86.92%	π→π*, n→π*
$S_0 \rightarrow T_3$	2.90 eV/428 nm	0.0000	56→60	69.71%	n→π*
			57→60	18.76%	n→π*
			52→60	8.25%	n→π*
$S_0 \rightarrow T_4$	3.28 eV/378 nm	0.0000	57→60	71.39%	n→π*
			56→60	17.20%	n→π*
$S_0 \rightarrow T_5$	3.61 eV/343 nm	0.0000	55→60	67.59%	π→π*, n→π*
			59→61	21.90%	π→π*, n→π*

[a] Orbital transition contribution during excitation.

Table S6-1b Electronic transitions involved in the excitation of Fl calculated at CAM-B3LYP/6-311G(d) level of theory.

	Energy	f	Composition	CI[c]	Character
$S_0 \rightarrow S_1$	3.4258 eV/361.91 nm	0.3385	59 → 60	0.69794	π→π*,n→π*
$S_0 \rightarrow S_2$	3.7314 eV/332.28 nm	0.0014	52 → 60	0.17627	n→π*
			56 → 60	0.49562	n→π*
			57 → 60	0.44861	n→π*
$S_0 \rightarrow S_3$	4.2272 eV/293.30 nm	0.0000	54 → 60	0.27741	n→π*
			54 → 63	0.10413	n→π*
			56 → 60	0.40692	n→π*
			57 → 60	0.44577	n→π*
			57 → 63	0.13391	n→π*
$S_0 \rightarrow S_4$	4.2905 eV/288.97 nm	0.1776	58 → 60	0.68343	π→π*,n→π*
			59 → 61	0.14822	π→π*,n→π*
$S_0 \rightarrow S_5$	5.0209 eV/246.94 nm	0.1173	53 → 60	0.23992	π→π*,n→π*
			55 → 60	0.48663	n→π*
			59 → 61	0.40244	π→π*,n→π*
$S_0 \rightarrow S_6$	5.0678 eV/244.65 nm	0.0001	52 → 60	0.29850	n→π*
			54 → 60	0.47776	n→π*
			56 → 60	0.20549	n→π*
			56 → 61	0.19740	n→π*
			56 → 65	0.10638	n→π*
			57 → 60	0.16024	n→π*
			57 → 61	0.12347	n→π*
$S_0 \rightarrow S_7$	5.2021 eV/238.33 nm	0.0001	52 → 60	0.50961	n→π*
			54 → 60	0.28077	n→π*
			56 → 61	0.19576	n→π*
			57 → 60	0.14898	n→π*
			57 → 61	0.21567	n→π*
$S_0 \rightarrow S_8$	5.3297 eV/232.63 nm	0.7328	55 → 60	0.4605	n→π*
			59 → 61	0.50447	π→π*,n→π*
$S_0 \rightarrow S_9$	5.4768 eV/226.38 nm	0.0223	53 → 60	0.44879	π→π*,n→π*
			55 → 60	0.16577	n→π*
			58 → 60	0.10048	π→π*,n→π*
			58 → 61	0.35326	π→π*,n→π*
			59 → 61	0.18744	π→π*,n→π*
			59 → 62	0.27446	π→π*,n→π*
$S_0 \rightarrow S_{10}$	5.9077 eV/209.87 nm	0.0612	53 → 60	0.46057	π→π*,n→π*
			53 → 62	0.10761	π→π*,n→π*
			58 → 61	0.26974	π→π*,n→π*
			59 → 62	0.41639	π→π*,n→π*
$S_0 \rightarrow T_1$	2.2932 eV/540.67 nm	0.0000	58 → 60	0.21460	π→π*,n→π*

			59 → 60	0.65825	π→π*,n→π*
S_0→T_2	2.8810 eV/430.35 nm	0.0000	58 → 60	0.56464	π→π*,n→π*
			58 → 62	0.21162	π→π*,n→π*
			59 → 60	0.21002	π→π*,n→π*
			59 → 61	0.24325	π→π*,n→π*
S_0→T_3	3.1258 eV/396.65 nm	0.0000	52 → 60	0.27122	n→π*
			56 → 60	0.47833	n→π*
			57 → 60	0.39553	n→π*
S_0→T_4	3.8959 eV/318.24 nm	0.0000	53 → 60	0.20230	π→π*,n→π*
			55 → 60	0.13346	n→π*
			58 → 60	0.26530	π→π*,n→π*
			59 → 61	0.57453	π→π*,n→π*
S_0→T_5	3.8988 eV/318.01 nm	0.0000	54 → 60	0.29867	n→π*
			54 → 63	0.14103	n→π*
			56 → 60	0.33699	n→π*
			57 → 60	0.45309	n→π*
			57 → 61	0.17204	n→π*

Table S6-1c Electronic transitions involved in the excitation of Fl calculated at ωB97XD/6-311G(d) level of theory.

	Energy	f	Composition	CI	Character
$S_0 \rightarrow S_1$	3.4125 eV/363.32 nm	0.3376	59 → 60	0.69666	π→π*
$S_0 \rightarrow S_2$	3.6942 eV/335.62 nm	0.0014	52 → 60	0.17244	n→π*
			56 → 60	0.53653	n→π*
			57 → 60	0.40097	n→π*
$S_0 \rightarrow S_3$	4.2027 eV/295.01 nm	0.0000	54 → 60	0.29631	n→π*
			54 → 63	0.10273	n→π*
			56 → 60	0.35871	n→π*
			57 → 60	0.47464	n→π*
			57 → 63	0.13612	n→π*
$S_0 \rightarrow S_4$	4.3031 eV/288.13 nm	0.1797	58 → 60	0.68243	π→π*,n→π*
			59 → 61	0.14651	π→π*,n→π*
$S_0 \rightarrow S_5$	5.0183 eV/247.06 nm	0.1002	53 → 60	0.25889	π→π*,n→π*
			55 → 60	0.48586	n→π*
			59 → 61	0.38532	π→π*,n→π*
			59 → 62	0.10225	π→π*,n→π*
$S_0 \rightarrow S_6$	5.0738 eV/244.36 nm	0.0001	52 → 60	0.39198	n→π*
			54 → 60	0.40620	n→π*
			56 → 60	0.20332	n→π*
			56 → 61	0.22287	n→π*
			56 → 64	0.10805	n→π*
			57 → 60	0.15454	n→π*
			57 → 61	0.10002	n→π*
$S_0 \rightarrow S_7$	5.2035 eV/238.27 nm	0.0001	52 → 60	0.44211	n→π*
			54 → 60	0.35937	n→π*
			56 → 61	0.17160	n→π*
			57 → 60	0.18192	n→π*
			57 → 61	0.22509	n→π*
			57 → 64	0.11865	n→π*
$S_0 \rightarrow S_8$	5.3407 eV/232.15 nm	0.7481	55 → 60	0.44529	n→π*
			58 → 60	0.10270	π→π*,n→π*
			59 → 61	0.51390	π→π*,n→π*
$S_0 \rightarrow S_9$	5.4715 eV/226.60 nm	0.0226	53 → 60	0.43508	π→π*,n→π*
			55 → 60	0.19103	n→π*
			58 → 60	0.10460	π→π*,n→π*
			58 → 61	0.34635	π→π*,n→π*
			59 → 61	0.19245	π→π*,n→π*
			59 → 62	0.27619	π→π*,n→π*
$S_0 \rightarrow S_{10}$	5.9508 eV/208.35 nm	0.0781	53 → 60	0.46236	π→π*,n→π*
			53 → 62	0.11869	π→π*,n→π*

			58 → 61	0.28675	π→π*,n→π*
			59 → 62	0.38889	π→π*,n→π*
S_0→T_1	2.3247 eV/533.34 nm	0.0000	58 → 60	0.18646	π→π*,n→π*
			59 → 60	0.66729	π→π*,n→π*
S_0→T_2	2.9693 eV/417.56 nm	0.0000	58 → 60	0.58469	π→π*,n→π*
			58 → 62	0.20198	π→π*,n→π*
			59 → 60	0.18038	π→π*,n→π*
			59 → 61	0.22453	π→π*,n→π*
S_0→T_3	3.1400 eV/394.86 nm	0.0000	52 → 60	0.26121	n→π*
			56 → 60	0.51552	n→π*
			57 → 60	0.35622	n→π*
S_0→T_4	3.8993 eV/317.96 nm	0.0000	54 → 60	0.31354	n→π*
			54 → 63	0.13683	n→π*
			56 → 60	0.29635	n→π*
			57 → 60	0.47511	n→π*
			57 → 63	0.17267	n→π*
S_0→T_5	3.9518 eV/313.74 nm	0.0000	53 → 60	0.20665	π→π*,n→π*
			55 → 60	0.13250	n→π*
			58 → 60	0.24516	π→π*,n→π*
			59 → 61	0.58257	π→π*,n→π*

Table S6-1d Electronic transitions involved in the excitation of Fl calculated at PBE0/6-311G(d) level of theory.

	Energy	f	Composition	CI[c]	Character
$S_0 \rightarrow S_1$	3.1338 eV/395.64 nm	0.2388	59 → 60	0.69666	π→π*,n→π*
$S_0 \rightarrow S_2$	3.4298 eV/361.49 nm	0.0010	56 → 60	0.55163	n→π*
			57 → 60	0.42332	n→π*
$S_0 \rightarrow S_3$	3.6299 eV/341.56 nm	0.0000	54 → 60	0.14944	n→π*
			56 → 60	0.41799	n→π*
			57 → 60	0.53617	n→π*
$S_0 \rightarrow S_4$	3.9069 eV/317.34 nm	0.1918	58 → 60	0.68432	π→π*,n→π*
			59 → 61	0.14127	π→π*,n→π*
$S_0 \rightarrow S_5$	4.3489 eV/285.09 nm	0.0000	54 → 60	0.66543	n→π*
			57 → 60	0.13974	n→π*
			57 → 61	0.11028	n→π*
$S_0 \rightarrow S_6$	4.4114 eV/281.05 nm	0.0124	55 → 60	0.67854	n→π*
			59 → 61	0.14983	π→π*,n→π*
$S_0 \rightarrow S_7$	4.8484 eV/255.72 nm	0.0003	52 → 60	0.66971	n→π*
			56 → 61	0.15943	n→π*
$S_0 \rightarrow S_8$	5.0209 eV/246.94 nm	0.2184	53 → 60	0.52345	π→π*,n→π*
			55 → 60	0.16085	n→π*
			58 → 61	0.20005	π→π*,n→π*
			59 → 61	0.37533	π→π*,n→π*
$S_0 \rightarrow S_9$	5.0328 eV/246.35 nm	0.5881	53 → 60	0.37536	π→π*,n→π*
			58 → 60	0.12618	π→π*,n→π*
			58 → 61	0.15638	π→π*,n→π*
			59 → 61	0.54562	π→π*,n→π*
$S_0 \rightarrow S_{10}$	5.4198 eV/228.76 nm	0.0003	52 → 60	0.15962	n→π*
			56 → 61	0.61925	n→π*
			57 → 61	0.26932	n→π*
$S_0 \rightarrow T_1$	2.1781 eV/569.23 nm	0.0000	58 → 60	0.14334	π→π*,n→π*
			59 → 60	0.68665	π→π*,n→π*
$S_0 \rightarrow T_2$	2.7278 eV/454.51 nm	0.0000	58 → 60	0.63218	π→π*,n→π*
			58 → 62	0.16973	π→π*,n→π*
			59 → 60	0.14028	π→π*,n→π*
			59 → 61	0.17408	π→π*,n→π*
$S_0 \rightarrow T_3$	2.9018 eV/427.27 nm	0.0000	52 → 60	0.21716	n→π*
			56 → 60	0.58522	n→π*
			57 → 60	0.29701	n→π*
$S_0 \rightarrow T_4$	3.3762 eV/367.24 nm	0.0000	54 → 60	0.20279	n→π*
			56 → 60	0.27453	n→π*
			57 → 60	0.58829	n→π*
			57 → 63	0.11003	n→π*

$S_0 \rightarrow T_5$	3.6745 eV/337.42 nm	0.0000	53 → 60	0.12277	π→π*,n→π*
			55 → 60	0.51342	n→π*
			55 → 63	0.12574	n→π*
			58 → 60	0.16485	π→π*,n→π*
			59 → 61	0.39960	π→π*,n→π*

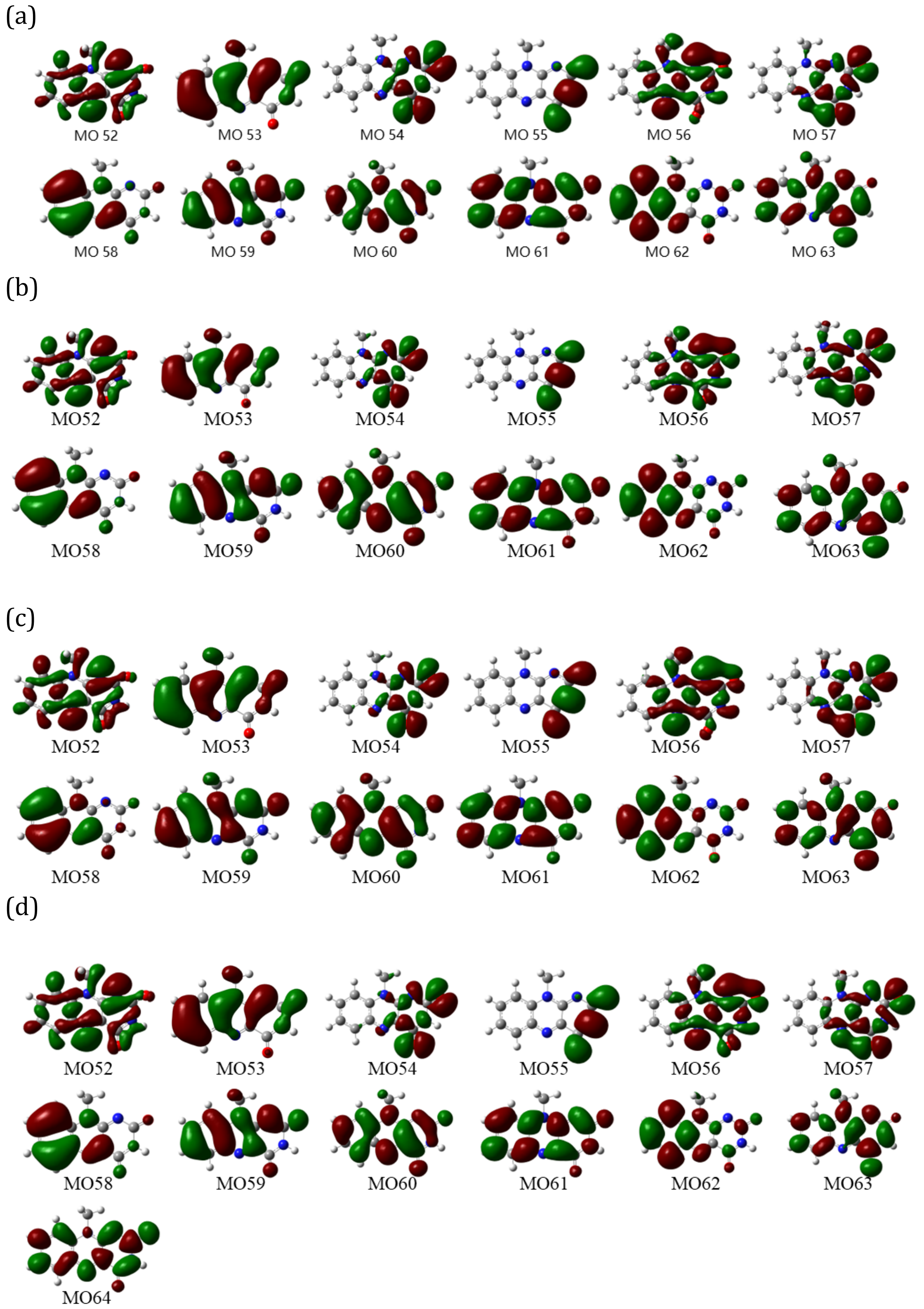

Fig. S6-1. Isosurface plots of frontier molecular orbitals of FL involved in electron transitions contribute to UV-vis absorption obtained with calculations at B3LYP/6-311g(d) (a), CAM-B3LYP/6-311g(d)(b), ωB97XD/6-311g(d)(c) and PBE0/6-311g(d) (d) level of theory. (Isovalue: ±0.02 a.u.; C: Gray; O: Red; N: Blue; H: White.)

Table S6-2. Electronic transitions involved in the excitation of dFl.

	Energy	f	Composition	Percentage	Character
$S_0 \rightarrow S_1$	3.28 eV/378 nm	0.2220	59→60	96.82%	π→π*, n→π*
$S_0 \rightarrow S_2$	3.82 eV/324 nm	0.0001	57→60	94.44%	n→π*
			54→60	3.17%	n→π*
$S_0 \rightarrow S_3$	4.03 eV/307 nm	0.1507	58→60	89.60%	π→π*, n→π*
			59→61	7.99%	π→π*, n→π*
$S_0 \rightarrow S_4$	4.26 eV/291 nm	0.0001	55→60	96.02%	n→π*
$S_0 \rightarrow S_5$	4.50 eV/276 nm	0.0000	54→60	89.27%	n→π*
$S_0 \rightarrow S_6$	4.54 eV/273 nm	0.0293	56→60	82.50%	π→π*, n→π*
			59→61	12.71%	π→π*, n→π*
$S_0 \rightarrow S_7$	4.92 eV/252 nm	0.7791	59→61	72.89%	π→π*, n→π*
			56→60	13.39%	π→π*, n→π*
$S_0 \rightarrow S_8$	5.05 eV/245 nm	0.0609	53→60	57.52%	π→π*, n→π*
			58→61	32.21%	π→π*, n→π*
$S_0 \rightarrow S_9$	5.29 eV/234 nm	0.0768	59→62	68.59%	π→π*, n→π*
			53→60	19.52%	π→π*, n→π*
$S_0 \rightarrow S_{10}$	5.31 eV/233 nm	0.0000	57→61	83.33%	n→π*
			54→60	3.49%	n→π*
$S_0 \rightarrow T_1$	2.53 eV/490 nm	0.0000	59→60	94.62%	π→π*, n→π*
$S_0 \rightarrow T_2$	2.92 eV/425 nm	0.0000	58→60	84.28%	π→π*, n→π*
			59→61	4.92%	π→π*, n→π*
$S_0 \rightarrow T_3$	3.64 eV/340 nm	0.0000	57→60	88.69%	n→π*
$S_0 \rightarrow T_4$	3.73 eV/332 nm	0.0000	59→61	78.38%	π→π*, n→π*
			58→60	6.56%	π→π*, n→π*
$S_0 \rightarrow T_5$	4.0 eV/310 nm	0.0000	56→60	78.53%	π→π*, n→π*
			56→63	4.55%	π→π*, n→π*

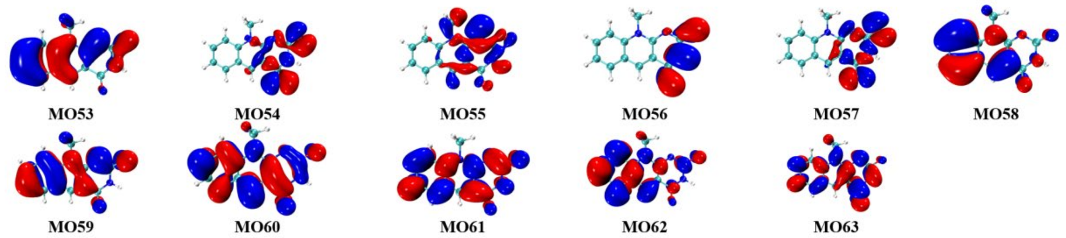

Fig. S6-2. Isosurface plots of frontier molecular orbitals of dFl involved in electron transitions contribute to UV-vis absorption. (Isovalue: ±0.02 a.u.; C: Cyan; O: Red; N: Blue; H: White.)

Table S6-3. Electronic transitions involved in the excitation of **TPAdFlMe**

	Energy	f	Composition	Percentage	Character
$S_0 \rightarrow S_1$	2.86 eV/433 nm	0.7205	107→108	98.30%	π → π *, n→ π *
$S_0 \rightarrow S_2$	3.41 eV/364 nm	0.0327	106→108	97.12%	π → π *, n→ π *
$S_0 \rightarrow S_3$	3.82 eV/324 nm	0.0000	105→108	84.96%	n→ π *
			104→108	9.36%	π → π *, n→ π *
$S_0 \rightarrow S_4$	3.93 eV/315 nm	0.1137	107→109	86.22%	π → π *, n→ π *
			104→108	5.26%	π → π *, n→ π *
$S_0 \rightarrow S_5$	4.17 eV/297 nm	0.0655	107→110	64.58%	π → π *, n→ π *
			104→108	27.20%	π → π *, n→ π *
$S_0 \rightarrow S_6$	4.23 eV/293 nm	0.1504	104→108	50.75%	π → π *, n→ π *
			107→110	27.36%	π → π *, n→ π *
$S_0 \rightarrow S_7$	4.33 eV/286 nm	0.0001	100→108	92.30%	n→ π *
$S_0 \rightarrow S_8$	4.34 eV/285 nm	0.0504	107→111	84.96%	π → π *, n→ π *
			102→108	5.60%	π → π *, n→ π *
$S_0 \rightarrow S_9$	4.37 eV/283 nm	0.0241	103→108	90.20%	π → π *, n→ π *
$S_0 \rightarrow S_{10}$	4.46 eV/278 nm	0.0385	102→108	77.14%	π → π *, n→ π *
			106→109	5.79%	π → π *, n→ π *
			107→111	5.25%	π → π *, n→ π *
$S_0 \rightarrow T_1$	2.25 eV/551 nm	0.0000	107→108	93.66%	π → π *, n→ π *
$S_0 \rightarrow T_2$	2.84 eV/437 nm	0.0000	106→108	86.38%	π → π *, n→ π *
			107→109	2.70%	π → π *, n→ π *
$S_0 \rightarrow T_3$	3.33 eV/372 nm	0.0000	104→108	30.55%	π → π *, n→ π *
			107→109	16.85%	π → π *, n→ π *
			102→108	9.22%	π → π *, n→ π *
$S_0 \rightarrow T_4$	3.39 eV/366 nm	0.0000	107→110	34.56%	π → π *, n→ π *
			107→109	14.29%	π → π *, n→ π *
			104→108	7.99%	π → π *, n→ π *
$S_0 \rightarrow T_5$	3.51 eV/353 nm	0.0000	107→109	41.94%	π → π *, n→ π *
			107→110	21.52%	π → π *, n→ π *
			104→108	5.88%	π → π *, n→ π *

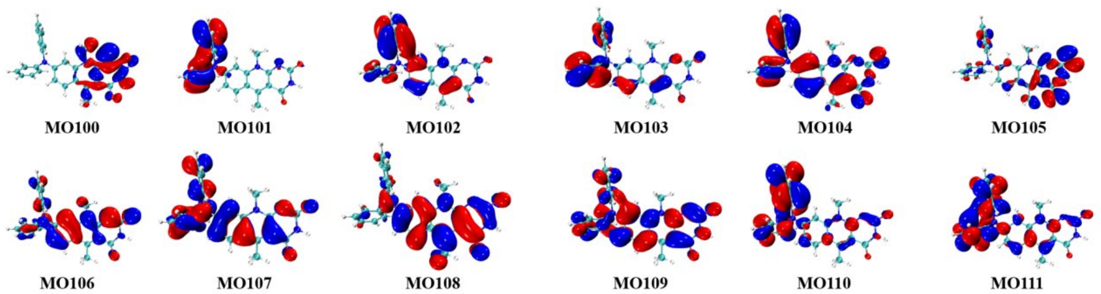

Fig. S6-3. Isosurface plots of frontier molecular orbitals of **TPAdFlMe** involved in electron transitions contribute to UV-vis absorption. (Isovalue: ±0.02 a.u.; C: Cyan; O: Red; N: Blue; H: White.)

Table S6-4. Electronic transitions involved in the excitation of **TPAdFlTF**.

	Energy	f	Composition	Percentage	Character
$S_0 \rightarrow S_1$	2.56 eV/485 nm	0.6648	119→120	98.80%	π→π*, n→π*
$S_0 \rightarrow S_2$	3.06 eV/406 nm	0.0405	118→120	97.40%	π→π*, n→π*
$S_0 \rightarrow S_3$	3.52 eV/353 nm	0.0016	116→120	78.33%	n→π*
			117→120	7.69%	π→π*, n→π*
			115→120	7.32%	π→π*, n→π*
$S_0 \rightarrow S_4$	3.78 eV/328 nm	0.0324	117→120	80.22%	π→π*, n→π*
			119→121	6.20%	π→π*, n→π*
$S_0 \rightarrow S_5$	3.92 eV/317 nm	0.0117	115→120	59.46%	π→π*, n→π*
			119→121	19.92%	π→π*, n→π*
$S_0 \rightarrow S_6$	3.96 eV/313 nm	0.0107	112→120	53.70%	n→π*
			111→120	26.54%	n→π*
			119→121	10.85%	π→π*, n→π*
$S_0 \rightarrow S_7$	4.01 eV/309 nm	0.0455	114→120	42.36%	π→π*, n→π*
			119→121	24.51%	π→π*, n→π*
			115→120	21.62%	π→π*, n→π*
$S_0 \rightarrow S_8$	4.04 eV/306 nm	0.0337	114→120	47.52%	π→π*, n→π*
			119→121	28.22%	π→π*, n→π*
$S_0 \rightarrow S_9$	4.20 eV/295 nm	0.0096	113→120	80.33%	π→π*, n→π*
			119→122	13.42%	π→π*, n→π*
$S_0 \rightarrow S_{10}$	4.22 eV/294 nm	0.0008	119→120	47.72%	π→π*, n→π*
			111→120	33.14%	n→π*
$S_0 \rightarrow T_1$	1.85 eV/671 nm	0.0000	119→120	92.77%	π→π*, n→π*
$S_0 \rightarrow T_2$	2.51 eV/493 nm	0.0000	118→120	90.23%	π→π*, n→π*
			119→120	2.32%	π→π*, n→π*
$S_0 \rightarrow T_3$	3.07 eV/404 nm	0.0000	117→120	38.24%	π→π*, n→π*
			114→120	21.20%	π→π*, n→π*
			109→120	10.32%	π→π*, n→π*

$S_0 \rightarrow T_4$	3.35 eV/370 nm	0.0000	116→120	70.97%	π→π*, n→π*
			115→120	9.09%	π→π*, n→π*
$S_0 \rightarrow T_5$	3.42 eV/362 nm	0.0000	119→121	37.13%	π→π*, n→π*
			119→122	13.24%	π→π*, n→π*
			119→123	12.48%	π→π*, n→π*

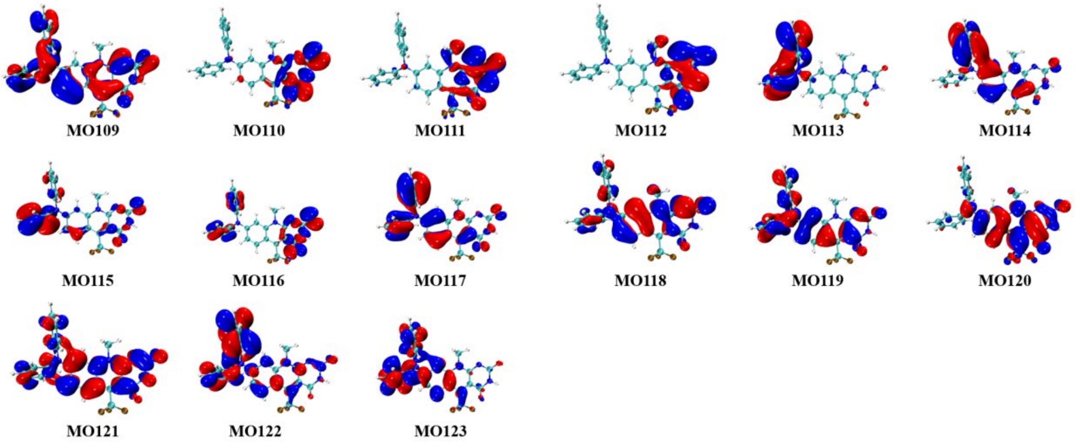

Fig. S6-4. Isosurface plots of frontier molecular orbitals of **TPAdFlTF**. involved in electron transitions contribute to UV-vis absorption. (Isovalue: ±0.02 a.u.; C: Cyan; O: Red; N: Blue; H: White.)

Table S6-5. Electronic transitions involved in the excitation of **TPAdFlPh**.

	Energy	f	Composition	Percentage	Character
$S_0 \rightarrow S_1$	2.86 eV/434 nm	0.7562	123→124	98.24%	π→π*, n→π*
$S_0 \rightarrow S_2$	3.39 eV/366 nm	0.0240	122→124	93.90%	π→π*, n→π*
			121→124	3.10%	n→π*
$S_0 \rightarrow S_3$	3.40 eV/364 nm	0.0019	121→124	94.77%	n→π*
			122→124	3.04%	π→π*, n→π*
$S_0 \rightarrow S_4$	3.91 eV/317 nm	0.0708	123→125	66.26%	π→π*, n→π*
			120→124	28.16%	π→π*, n→π*
$S_0 \rightarrow S_5$	3.96 eV/313 nm	0.0415	120→124	51.80%	π→π*, n→π*
			119→124	22.65%	π→π*, n→π*
			123→125	20.85%	π→π*, n→π*
$S_0 \rightarrow S_6$	4.09 eV/303 nm	0.0000	117→124	94.45%	n→π*
$S_0 \rightarrow S_7$	4.17 eV/297 nm	0.0270	119→124	42.84%	π→π*, n→π*
			123→126	41.93%	π→π*, n→π*
			120→124	9.03%	π→π*, n→π*
$S_0 \rightarrow S_8$	4.22 eV/294 nm	0.1952	123→126	49.99%	π→π*, n→π
			119→124	29.18%	π→π*, n→π*
			120→124	7.88%	π→π*, n→π**
$S_0 \rightarrow S_9$	4.31 eV/287 nm	0.0001	113→124	94.26%	
$S_0 \rightarrow S_{10}$	4.34 eV/285 nm	0.0326	118→124	84.12%	π→π*, n→π*
			123→127	8.06%	π→π*, n→π*
$S_0 \rightarrow T_1$	2.21 eV/561 nm	0.0000	123→124	93.62%	π→π*, n→π*
$S_0 \rightarrow T_2$	2.82 eV/440 nm	0.0000	122→124	87.17%	π→π*, n→π*
			123→125	2.59%	π→π*, n→π*
$S_0 \rightarrow T_3$	3.30 eV/376 nm	0.0000	121→124	88.79%	n→π*
$S_0 \rightarrow T_4$	3.32 eV/373 nm	0.0000	120→124	18.64%	π→π*, n→π*
			119→124	15.03%	π→π*, n→π*
			123→125	14.17%	π→π*, n→π*
			116→124	10.28%	π→π*, n→π*
$S_0 \rightarrow T_5$	3.40 eV/365 nm	0.0000	123→126	31.60%	π→π*, n→π*
			123→125	17.96%	π→π*, n→π*
			123→127	5.83%	π→π*, n→π*

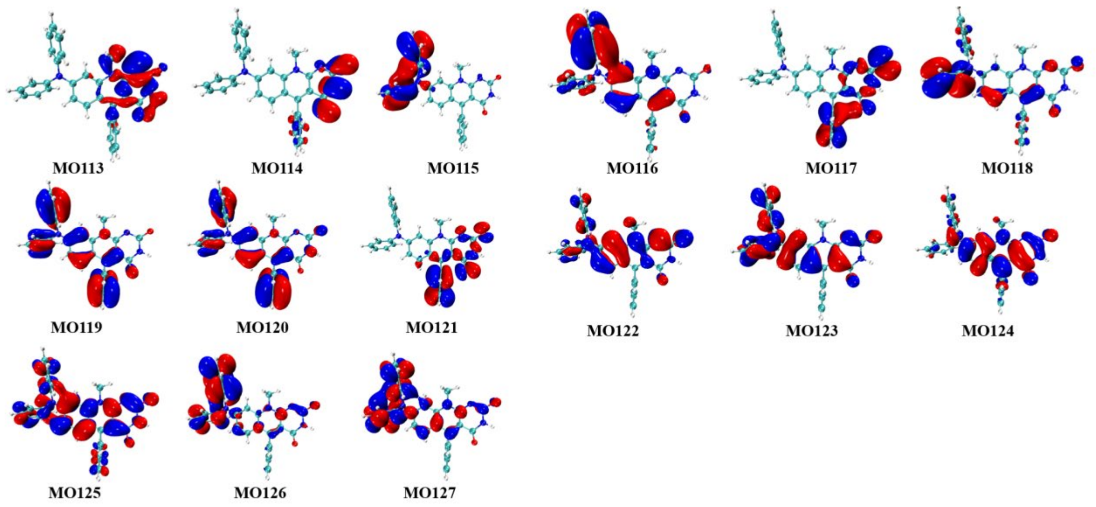

Fig. S6-5. Isosurface plots of frontier molecular orbitals of **TPAdFlPh** involved in electron transitions contribute to UV-vis absorption. (Isovalue: ±0.02 a.u.; C: Cyan; O: Red; N: Blue; H: White.)

7. References

1. Binning, R. C.; Curtiss, L. A., COMPACT CONTRACTED BASIS-SETS FOR 3RD-ROW ATOMS - GA-KR. *Journal of Computational Chemistry* **1990,** (10), 1206-1216.

2. Francl, M. M.; Pietro, W. J.; Hehre, W. J.; Binkley, J. S.; Gordon, M. S.; Defrees, D. J.; Pople, J. A., Self-consistent Molecular-orbital Methods 23. A Polarization-type Basis Set for 2nd-row Elements. *Journal of Chemical Physics* **1982,** *77* (7), 3654-3665.

3. Becke, A. D., Density-functional Thermochemistry 3. The role of exact exchange. *Journal of Chemical Physics* **1993,** *98* (7), 5648-5652.

4. Lee, C. T.; Yang, W. T.; Parr, R. G., Development of the Colle-Salvetti Correlations-energy Formula into a Functional of the Electron-density. *Physical Review B* **1988,** *37* (2), 785-789.

5. Tomasi, J.; Mennucci, B.; Cammi, R., Quantum mechanical continuum solvation models. *Chemical Reviews* **2005,** *105* (8), 2999-3093.

6. Frisch, M. J.; Trucks, G. W.; Schlegel, H. B.; Scuseria, G. E.; Robb, M. A.; Cheeseman, J. R.; Scalmani, G.; Barone, V.; Petersson, G. A.; Nakatsuji, H.; Li, X.; Caricato, M.; Marenich, A. V.; Bloino, J.; Janesko, B. G.; Gomperts, R.; Mennucci, B.; Hratchian, H. P.; Ortiz, J. V.; Izmaylov, A. F.; Sonnenberg, J. L.; Williams; Ding, F.; Lipparini, F.; Egidi, F.; Goings, J.; Peng, B.; Petrone, A.; Henderson, T.; Ranasinghe, D.; Zakrzewski, V. G.; Gao, J.; Rega, N.; Zheng, G.; Liang, W.; Hada, M.; Ehara, M.; Toyota, K.; Fukuda, R.; Hasegawa, J.; Ishida, M.; Nakajima, T.; Honda, Y.; Kitao, O.; Nakai, H.; Vreven, T.; Throssell, K.; Montgomery Jr., J. A.; Peralta, J. E.; Ogliaro, F.; Bearpark, M. J.; Heyd, J. J.; Brothers, E. N.; Kudin, K. N.; Staroverov, V. N.; Keith, T. A.; Kobayashi, R.; Normand, J.; Raghavachari, K.; Rendell, A. P.; Burant, J. C.; Iyengar, S. S.; Tomasi, J.; Cossi, M.; Millam, J. M.; Klene, M.; Adamo, C.; Cammi, R.; Ochterski, J. W.; Martin, R. L.; Morokuma, K.; Farkas, O.; Foresman, J. B.; Fox, D. J. *Gaussian 16 Rev. A.03*, Wallingford, CT, 2016.
